# Propagation of α-Synuclein Strains within Human Reconstructed Neuronal Network

**DOI:** 10.1016/j.stemcr.2018.12.007

**Published:** 2019-01-10

**Authors:** Simona Gribaudo, Philippe Tixador, Luc Bousset, Alexis Fenyi, Patricia Lino, Ronald Melki, Jean-Michel Peyrin, Anselme L. Perrier

**Affiliations:** 1 INSERM U861, I-STEM, AFM, Corbeil-Essonnes 91100, France; 2 UEVE U861, I-STEM, AFM, Corbeil-Essonnes 91100, France; 3 Sorbonne Universités, Faculté des Sciences et Ingénierie, CNRS/UMR 8256, B2A, Biological Adaptation and Ageing, Institut de Biologie Paris Seine, Paris 75005, France; 4 Laboratory of Neurodegenerative Disease, Institut François Jacob, MIRCen, CEA-CNRS, Fontenay aux Roses 92265, France

**Keywords:** human pluripotent stem cells, Parkinson's disease, microfluidic, prion-like, nucleation, synuclein, Lewy body, human cortical neuron, neuronal dysfunction

## Abstract

Reappraisal of neuropathological studies suggests that pathological hallmarks of Alzheimer’s disease and Parkinson’s disease (PD) spread progressively along predictable neuronal pathways in the human brain through unknown mechanisms. Although there is much evidence supporting the prion-like propagation and amplification of α-synuclein (α-Syn) *in vitro* and in rodent models, whether this scenario occurs in the human brain remains to be substantiated. Here we reconstructed in microfluidic devices corticocortical neuronal networks using human induced pluripotent stem cells derived from a healthy donor. We provide unique experimental evidence that different strains of human α-Syn disseminate in “wild-type” human neuronal networks in a prion-like manner. We show that two distinct α-Syn strains we named fibrils and ribbons are transported, traffic between neurons, and trigger to different extents, in a dose- and structure-dependent manner, the progressive accumulation of PD-like pathological hallmarks. We further demonstrate that seeded aggregation of endogenous soluble α-Syn affects synaptic integrity and mitochondria morphology.

## Highlights

• Different α-Syn strains propagate within WT human iPSC-derived cortical neuronal networks
• α-Syn strains differentially seed endogenous WT α-Syn forming LB/LN-like structures
• Phospho-α-Syn endogenous aggregates resist degradation and accumulate in cytoplasm
• Accumulation of phospho-α-Syn induces early neuronal dysfunctions

## Graphical Abstract

**Figure:**
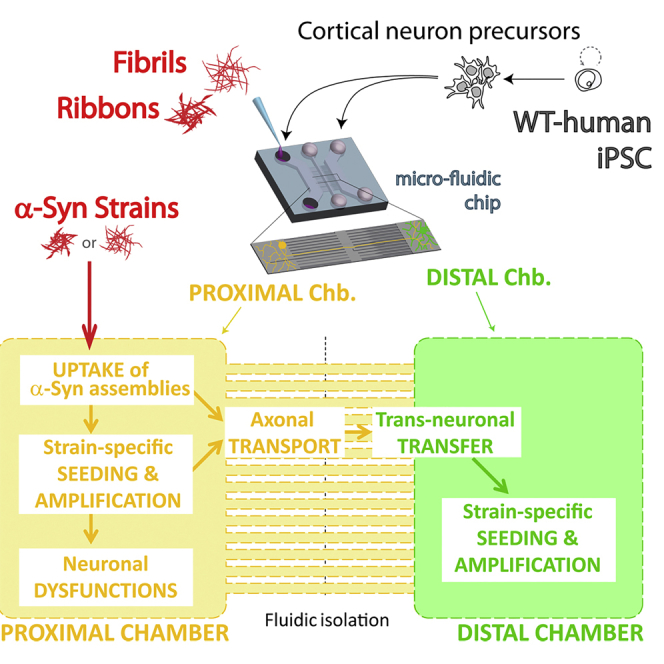

## Teaser

In this article, Gribaudo and colleagues report the internalization and trafficking of exogenous α-synuclein fibrils and ribbons through fluidically isolated cortical networks derived from wild-type human iPSCs. These assemblies trigger via seeding mechanisms the accumulation of endogenous phosphorylated α-synuclein, in a structure-, dose-, and time-dependent manner. Phosphorylated α-Syn structures resist degradation and accumulate in the cytoplasm of neurons, affecting Ca^2+^ homeostasis and mitochondria morphology.

## Introduction

Chronic neurodegenerative diseases are tightly linked to the progressive accumulation of abnormally folded protein conformers inside or outside neurons. These abnormal conformers are proposed to mediate neuronal dysfunctions. In synucleinopathies such as Parkinson's disease (PD), dementia with Lewy bodies (DLB), and multiple system atrophy (MSA), α-synuclein (α-Syn), a presynaptic protein involved in the regulation of synaptic vesicle release, accumulates in a phosphorylated and insoluble form in neurons. Aggregated α-Syn is the principal component of Lewy bodies and Lewy neurites, which are considered pathognomonic hallmarks of synucleinopathies (Goedert et al., 2013, Spillantini et al., 1997). As in Alzheimer’s disease (Braak et al., 2006), PD/DLB/MSA pathological hallmarks progress spatially and temporally in the brains of affected patients (Braak et al., 2003b). This, together with the observation that Lewy bodies are detected in neurons grafted in PD patients (Kordower et al., 2008, Li et al., 2008), suggests that neuropathological hallmarks spread from diseased to naive neurons through unknown mechanisms.

Mounting studies show that α-Syn assemblies trigger the aggregation of soluble α-Syn both *in vitro* and *in vivo*, suggesting that aggregated α-Syn holds prion-like properties in rodents (Desplats et al., 2009, George et al., 2013, Guo et al., 2013, Hansen et al., 2011, Luk et al., 2012a, Peelaerts et al., 2015, Sacino et al., 2014a, Volpicelli-Daley et al., 2011). Indeed, intra-cerebral inoculation of brain homogenates from aged transgenic mice overexpressing mutant α-Syn to cognate young transgenic animals susceptible to spontaneous α-Syn aggregation or to wild-type (WT) mice leads to early motor deficits and α-Syn deposition in the rodent central nervous system (Betemps et al., 2014, Luk et al., 2012b, Masuda-Suzukake et al., 2013). Yet whether PD-associated disease hallmarks spread through a prion-like process in human brain remains to be substantiated. The extent to which fibrillar α-Syn assemblies bind to human mature neurons, are taken up, seed the aggregation of their soluble counterparts, trigger neuronal dysfunctions, and propagate from one neuron to another remains to be determined. Human pluripotent stem cell (hPSC) technologies have become a key asset for deciphering pathological mechanisms (Grskovic et al., 2011). Several proofs of concept have shown that hPSC differentiation into neurons is paramount to study neurodegenerative syndromes as it provides direct access to cultures enriched in human post-mitotic neurons with region/subtype-specific identities (Gaspard et al., 2009). Previously, we (1) demonstrated the reconstruction of fully functional and oriented rodent neuronal networks in microfluidic chips (Deleglise et al., 2014, Dinh et al., 2013, Peyrin et al., 2011), (2) gained unlimited access to standardized human telencephalic neurons from hPSCs (Nicoleau et al., 2013), and (3) demonstrated uptake and active anterograde and retrograde transport of fibrillar α-Syn in mouse neurons (Brahic et al., 2016, Freundt et al., 2012). Here, taking advantage of these studies we reconstructed human induced pluripotent stem cell (hiPSC)-derived neuronal networks in microfluidic cell culture chambers and demonstrated that two structurally and functionally distinct α-Syn strains we named ribbons and fibrils (Bousset et al., 2013, Peelaerts et al., 2015) can efficiently propagate through such human WT neuronal networks. Both strains are able to reach the cytoplasmic compartment after direct internalization or upon neuron-to-neuron-mediated transfer (Abounit et al., 2016, Flavin et al., 2017) and elicit endogenous α-Syn aggregation in human cortical neurons. This process is progressive, and dose and structure dependent, with ribbons bearing a more potent seeding activity over a few weeks. Accumulation of α-Syn somatic inclusions correlates with neuronal activity impairment and mitochondrial dysfunctions. This model combining human connected WT neurons and a trigger input is representative of the sporadic form of synucleinopathies and indicates that α-Syn prion-like spreading occurs in human neuronal networks where α-Syn expression is physiological and may account for the progression of age-related neurodegeneration in humans. This model represents a significant improvement over previous models where WT or mutant α-Syn is overexpressed in rodents (Luk et al., 2012b, Peelaerts et al., 2015).

## Results

### Reconstruction of Oriented Human Corticocortical Network

**Figure 1:**
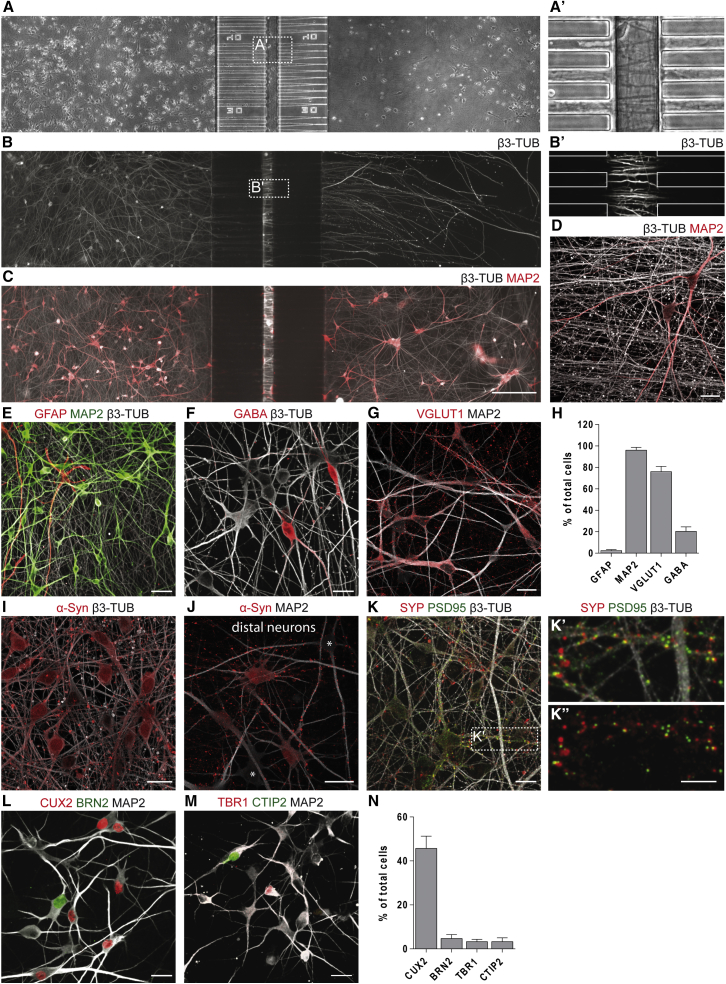
Characterization of hiPSC-Derived Corticocortical Neuronal Networks (A) Phase-contrast stitched image of hiPSC-derived neuronal networks 30 days post-plating. (A') Inset of central chamber invaded by axons. (B–D) (B and C) Fluorescence images (white, β3-tubulin [β3-TUB]; red, MAP2) of hiPSC-derived neurons in the proximal chamber projecting their axons into the distal chamber (B) in the absence or (C) in the presence of target neurons. (B′ and D) Higher-magnification images showing (B′) axons crossing the central chamber of the microchannel compartment and (D) axon bundles exiting the microchannel in the distal chamber. (E–H) Representative images and quantification of astrocytic (GFAP) and neuronal (MAP2, β3-TUB, GABA, VGLUT1) marker expression. (E) GFAP, MAP2, β3-TUB; (F) GABA, β3-TUB; (G) VGLUT1, MAP2; (H) percentage of cells expressing each marker. (I and J) (I) Expression of α-Syn in nuclei and perikarya of neurons (asterisks point out negative cells) and enrichment in synaptic boutons (J, distal chamber). (K–K″) (K) Identification of putative excitatory synapses by juxtaposition of pre- (red, SYP) and post- (green, PSD95) protein complexes along neurites (white, β3-TUB). (K′) Magnification of box indicated in (K). (L–N) Representative images and quantification of a repertoire of distinct cortical neuronal markers. (L) CUX2, BRN2, MAP2; (M) TBR1, CTIP2, MAP2; (N) percentage of cells expressing each marker. Data are shown as mean ± SEM; n = 3. Scale bars: 200 μm in (A–C), 20 μm in (A′, B′, and D), 40 μm in (E), 20 μm in (F, G, and I–M), 10 μm in (K′ and K″).

To assess α-Syn assembly propagative properties in a human neuronal network we sought to combine neurons generated from hiPSCs derived from a healthy donor with microfluidic technologies allowing the reconstruction of an oriented neuronal network. We generated dorsal telencephalic neuronal progenitors from hPSCs (iPSC lines i90cl17 and i90cl16) as previously described (Nicoleau et al., 2013) and differentiated them into post-mitotic neurons (Feyeux et al., 2012). Our microfluidic devices are composed of three independent cell culture chambers separated by asymmetric microchannels that allow unidirectional axonal growth from the emitting to the receiving chamber (Figures 1A and 1A′) (Deleglise et al., 2013, Peyrin et al., 2011). hiPSC-derived neurons extended neurites through the microchannel that surrounded neurons in the distal chambers within 4 weeks of plating (Figures 1A'–1D). Phenotype and cortical regional identity of human neurons were assessed by immunofluorescence after 30 days of differentiation in chips. These hiPSC-derived cultures were highly enriched in neurons (>90%, MAP2^+^), with a small percentage differentiating into astrocytes (<3%, GFAP^+^) (Figures 1E and 1H). The neurons were composed of about 20% inhibitory GABA^+^ neurons (Figures 1F and 1H) and about 75% glutamatergic VGlut1^+^ neurons (Figures 1G and 1H). We detected α-Syn expression in nuclei and perikarya of the majority of neurons (Figures 1I and 1J), with enrichment in synaptic boutons particularly visible in the distal chamber, where axons from proximal neurons converged on the resident cells (Figure 1J). Juxtaposition of pre-synaptic (synaptophysin, SYP) and post-synaptic density protein-95 (PSD95) staining along dendrites was detected as well, indicating the establishment of excitatory synapses (Figures 1K–1K″). Those neurons expressed a variety of cortical markers, including upper- (BRN2, CUX2; Figures 1L and 1N) and, to a lesser extent, deep-layer cortical markers (TBR1, CTIP2; Figures 1M and 1N). Altogether, our results indicate that mature oriented human corticocortical networks reminiscent of neuroanatomical pathways within human brain can be reliably obtained in the microfluidic devices we used.

### Axonal Transport and Inter-neuronal Spreading of α-Syn Assemblies in Reconstructed Human Corticocortical Networks

We used our setup to investigate in a human context the properties of two distinct α-Syn strains, coined as fibrils and ribbons (Bousset et al., 2013), that have been previously shown to trigger distinct synucleinopathies in rodents (Peelaerts et al., 2015). To assess their prion-like behavior in hiPSC-derived cortical neurons, we specifically measured the uptake, intra-cellular transport, inter-neuronal transfer, and seeding propensity of each strain.

**Figure 2:**
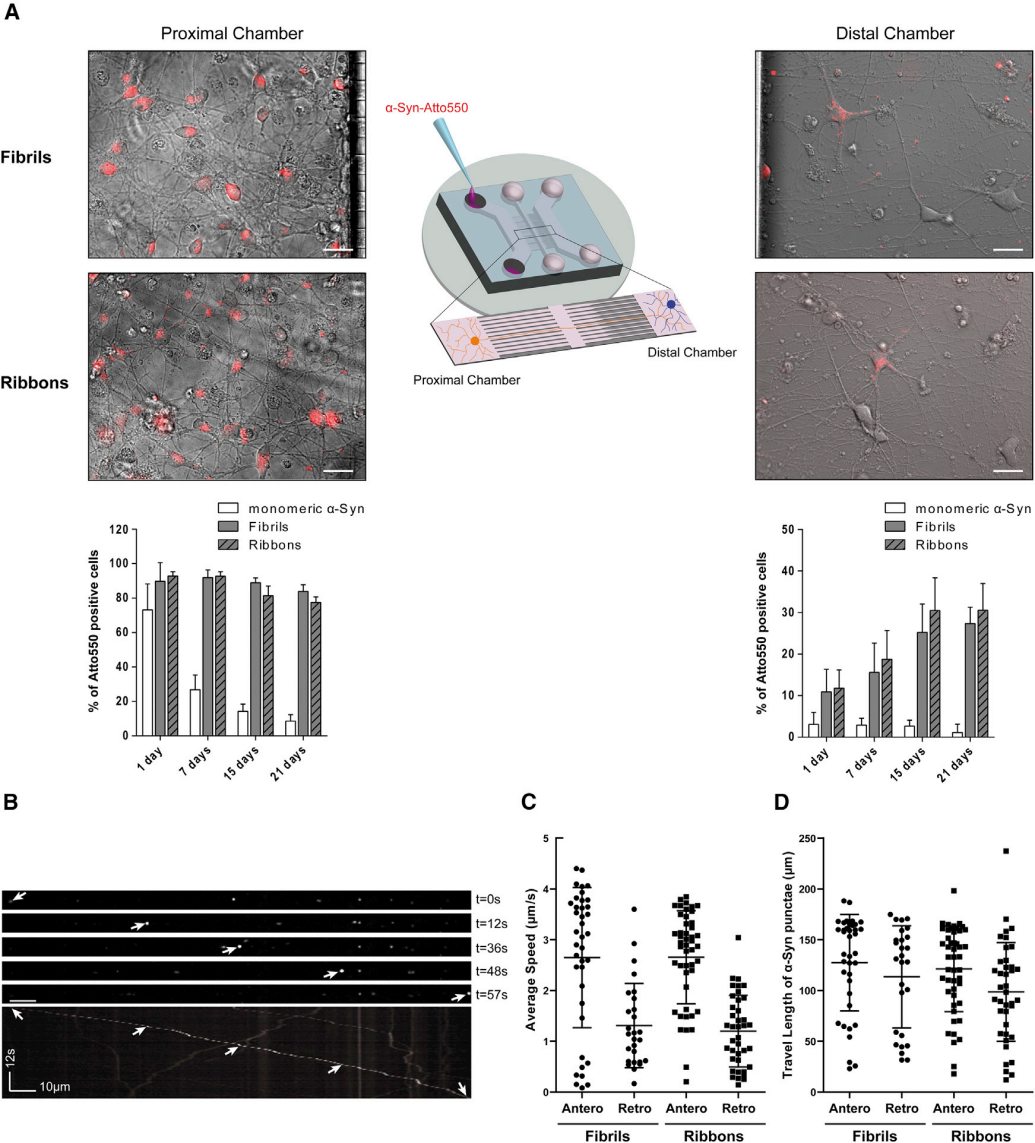
Intra-neuronal and Inter-neuronal Spreading of α-Syn Assemblies (A) Fluorescence and phase-contrast images of 30 day old corticocortical neuronal networks exposed for 24 h in the proximal chamber (left) to 500 nM Atto-550-labeled fibrils (top) or ribbons (bottom). The bar graphs show quantitative assessment of Atto-550-labeled α-Syn monomers, fibrils, and ribbons taken up by loaded neurons (left) and transferred to receiving neurons (right) at 1, 7, 15, and 21 dpe. Significant differences were observed between strains and between days post-exposure in both proximal and distal chambers (for statistical analysis see Supplemental Information). Data are shown as ± SD. (B) Kymograph analysis of α-Syn fibril movement along axons extending from the proximal chamber in a typical recording performed at 24 h post-loading. (C and D) Analysis of α-Syn assemblies' anterograde and retrograde movement in axonal tracts. (C) Average speed. (D) Travel length. Data are shown as mean ± SD. Scale bars: 25 μm.

To determine whether α-Syn assemblies could be taken up and transported along the axons, neurons within the proximal chamber of 30 day old reconstructed human corticocortical networks were exposed to Atto-550-labeled α-Syn monomers, fibrils, or ribbons. The fluorescent signal was longitudinally analyzed by live imaging microscopy in both proximal and distal microfluidic chambers 1, 7, 15, and 21 days post-exposure (dpe). Starting from 1 dpe about 90% of the neurons in the proximal chamber exposed to both ribbons and fibrils clearly presented Atto-550 dots in their cytoplasm that persisted over time. In contrast, cells exposed to monomeric α-Syn exhibited a diffuse Atto-550 signal with very few puncta that were almost completely lost over time (Figure 2A, left), suggesting that monomeric and fibrillar α-Syn have different clearance dynamics. To gain molecular insight into fibrillar α-Syn uptake, we exposed neurons to monocadaverin, an inhibitor of receptor-mediated endocytosis, or to dynasore, an inhibitor of dynamin, prior to addition of the exogenous α-Syn fibrillar strains. Uptake of Atto-550 α-Syn strains (fibrils and ribbons) was strongly reduced by monocadaverin (70 μM), while dynasore (80 μM) only partially reduced uptake (Figure S2A). Ordered movements of small fluorescent exogenous α-Syn puncta were evidenced in the axonal extension crossing the microchannel barrier. We distinguished between anterograde and retrograde axonal transport of fluorescent α-Syn strains, performing time-lapse imaging at the exit of microchannels in the distal chamber. Both strains were transported anterogradely and retrogradely in human neurons (Figures 2B and 2C and Videos S1 and S2). The mean anterograde velocities were 2.6 ± 1.4 and 2.7 ± 0.9 μm/s for fibrils and ribbons, respectively, consistent with the fast component of axonal anterograde transport. Assemblies transported retrogradely moved at 1.3 ± 0.8 μm/s for the fibrils and 1.2 ± 0.7 μm/s for ribbons (Figure 2C), thus creating a net axonal anterograde flux upon somatodendritic loading. During the time of recording, the two α-Syn strains displayed similar average run lengths (98–128 μm) (Figure 2D).

Video S1. Axonal Transport of α-Syn Ribbons, Related to Figure 2 Axonal transport of α-Syn ribbons (Video S1) or fibrils (Video S2) visualized at the exit of the microgrooves in the distal chamber.

Video S2. Axonal Transport of α-Syn Fibrils, Related to Figure 2 Axonal transport of α-Syn ribbons (Video S1) or fibrils (Video S2) visualized at the exit of the microgrooves in the distal chamber.

We next quantified the spread of α-Syn strains from neurons in the proximal chamber to connected neurons in the distal chamber (Figure 2A, right). Again, the proportion of neurons with detectable signal from Atto-550-labeled monomeric α-Syn was very low and almost disappeared over time, whereas that of neurons presenting Atto-550 dots in their soma after exposing afferent neurons in the proximal chamber to fibrils or ribbons significantly increased up to 15 dpe, and then plateaued (Figure 2A, right). Similar inter-neuronal transfer capacities were observed for the two α-Syn strains. Monocadaverin and dynasore affected inter-neuronal transfer (from the proximal to the distal chambers of the microfluidic device) of Atto-550 α-Syn strains (Figures S2B and S2C). Thus, endocytosis appears to contribute significantly to α-Syn assembly spreading between human cortical neurons. Overall, these data demonstrate that α-Syn strain spread between human neurons is a fast and efficient process involving internalization followed by anterograde axonal transport to the axonal ends and spread to secondary neurons. Interestingly, the efficiency of uptake, transport, and inter-neuronal transfer over time was similar for fibril and ribbon strains (Figure 2A).

### Seeding of Endogenous α-Syn in Human Cortical Wild-Type Neurons

Seeding and accumulation of phosphorylated host-encoded cognate α-Syn following trans-neuronal propagation is key for the concept of prion-like spreading along nerve pathways in PD. We therefore assessed whether exogenous human α-Syn strains provide a template for the assembly of endogenous WT human α-Syn into aggregates characteristic of pathological α-Syn. Mature (30 day old) hiPSC-derived cortical neurons were exposed to monomeric α-Syn, ribbons, or fibrils for 24 h and incubated for 30 days. Additional control conditions included human cortical neurons exposed to Atto-550-labeled fibrillar human huntingtin exon 1 with 48 glutamine residues (HTTExon1Q48). We used both immunofluorescence and biochemical analysis to assess the formation of abnormal α-Syn, monitoring in particular its detergent insolubility and its phosphorylation at position serine 129 (S129).

**Figure 3:**
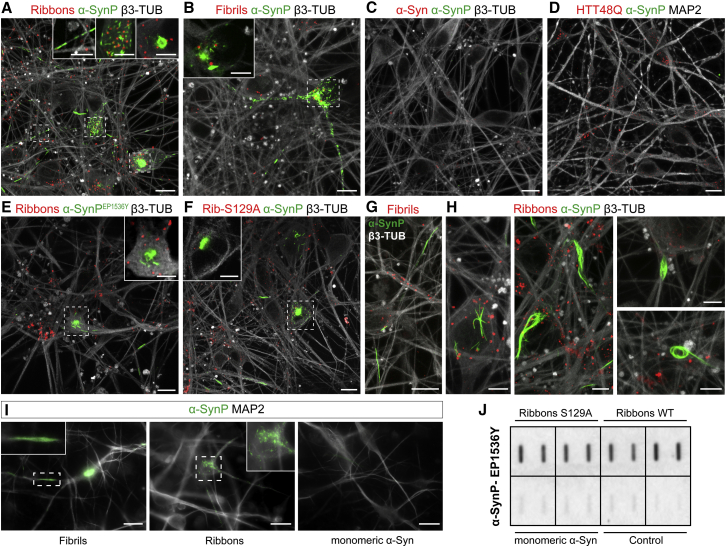
Immunofluorescence Analysis of Seeded Endogenous α-Syn Aggregation in hiPSC-Derived Cortical Neurons (A–F) Fluorescence confocal imaging of endogenous α-SynP (green) in human cortical neurons (β3-TUB or MAP2, gray) at 30 dpe to 500 nM Atto-550 (red) (A and E) α-Syn ribbons, (B) α-Syn fibrils, (C) monomeric α-Syn, (D) HTTExon1Q48, and (F) S129A ribbons using two different α-SynP antibodies (α-SynP, ab59264 in A–D and F–H, and α-SynP^EP1536Y^, ab51253 in E). The regions boxed with dashed lines are enlarged and represented as single plane in the insets. Scale bars: 10 μm, 5 μm in insets. (G and H) Representative images showing that α-SynP inclusions triggered by either (G) fibrils or (H) ribbons exhibit different classes of shapes (green, α-SynP; white, β3-TUB; red, exogenous assemblies); scale bar: 10 μm in (G), 5 μm in (H). (I) Resistance of endogenous α-SynP signal to paraformaldehyde/Triton X-100 fixation/extraction immunofluorescence protocol (green, α-SynP; white, MAP2); scale bar: 20 μm. (J) Filter trap assay for α-SynP (α-SynP^EP1536Y^ antibody) in cortical neuron lysates at 30 dpe to ribbons made of wild-type or S129A α-Syn or monomeric α-Syn or in the absence of treatment. Each square represents duplicates of the sample.

Thirty days after exposure of hiPSC-derived cortical neurons to α-Syn fibrils and ribbons, phosphorylated α-Syn (α-SynP) clumps were detected (Figures 3A and 3B). No α-SynP signal was detected upon neuronal exposure to either monomeric α-Syn or HTTExon1Q48 fibrillar assemblies (Figures 3C and 3D). These results were confirmed using a second α-SynP antibody (α-SynP EP1536Y), which was previously demonstrated not to cross-react with phosphorylated neurofilament (Rutherford et al., 2016, Sacino et al., 2014b) (Figure 3E).

We then challenged human cortical neurons with a version of α-Syn strain that retains its deleterious activity while being non-phosphorylatable at amino acid residue S129 because this residue was changed into an alanine (Febbraro et al., 2013, Gorbatyuk et al., 2008). After exposure to fibrillar S129A α-Syn exogenous assemblies, human mature neurons exhibited aberrant phosphorylation and clustering of the endogenous α-Syn (Figure 3F, and data not shown for α-SynP EP1536Y antibody). Altogether, these results show that exogenous α-Syn ribbons and fibrils trigger the specific formation of inclusions made of endogenous α-SynP in hiPSC-derived cortical neurons. Astroglial cells in our cultures readily took up the exogenous assemblies that strongly associate with lysosomal marker (Figure S3A) but, in contrast to neurons, none of those cells ever contained α-SynP structures (Figure S3B), thus evidencing that human astrocytes do not contribute actively to the amplification of α-Syn strains. Both strains promoted the formation of α-SynP inclusions in neurons bearing different aspects, including compact agglomerated, annular, and filiform/serpentine structures in both the somatodendritic compartment and the axonal endings (Figures 3A, 3B, and 3E–3H). Interestingly, α-SynP inclusions present in the somatic compartment were always found juxtaposed to exogenous Atto-550-labeled α-Syn or α-Syn S129A ribbons or fibrils (single-plane insets in Figures 3A, 3B, 3E, and 3F), suggesting a physical interaction between the exogenous input and the α-SynP somatic inclusions. The α-SynP inclusions formed upon fibril and ribbon exposure are resistant to a paraformaldehyde/Triton X-100 fixation/extraction and immunofluorescence protocol (Figure 3I), indicating that α-SynP morphotypes have an insoluble nature (Volpicelli-Daley et al., 2016).

These results were confirmed using a filter trap assay (Figure 3J), in which comparable amounts of α-SynP signal were reproducibly detected in samples originating from neurons exposed to WT or mutant S129A α-Syn ribbons. This further proves that the α-SynP we detect corresponds to seeded endogenous cortical neurons' α-Syn, not the exogenous seeds, as they are not recognized by the α-SynP S129 antibodies. We did not observe α-SynP in neurons exposed to monomeric α-Syn.

We next labeled oligodendrocytes and oligodendrocyte progenitors with the O4 antibodies in our human cortical cultures exposed to α-Syn assemblies (21 dpe). We observed that exogenous α-Syn assemblies are readily taken up by the few O4^+^ cells present in our cultures (Figure S4). In line with our *in vivo* observation in Peelaerts et al. (2015), no α-SynP inclusions could be detected in those O4^+^ cells (Luk et al., 2012b, Peelaerts et al., 2015).

### Characterization of α-SynP Inclusions in Human Wild-Type Neurons

**Figure 4:**
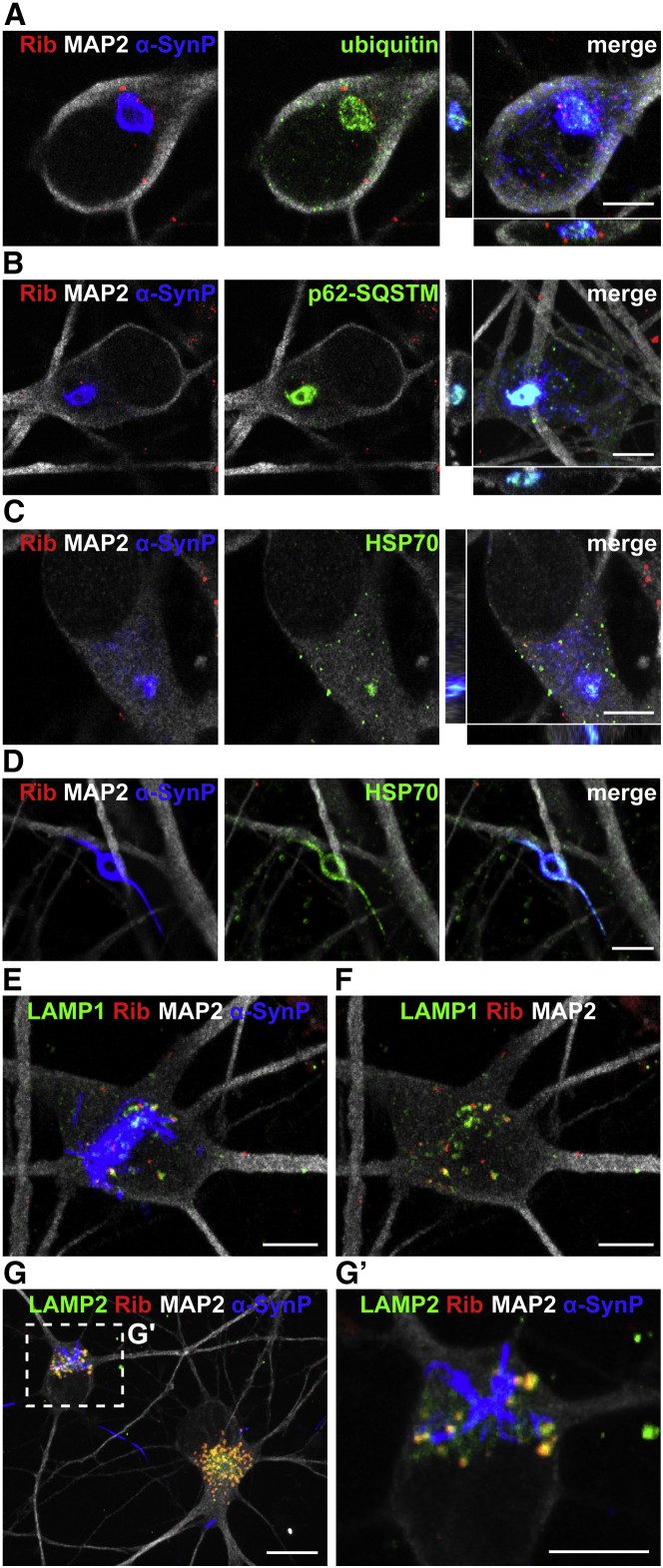
Characterization of α-SynP Somatodendritic Inclusions (A–D) (A) Anti-ubiquitin, (B) anti-p62-sequestosome1, and (C and D) anti-HSP-70 immunostaining (green) and colocalization with endogenous α-SynP aggregates (blue) in neurons (MAP2, white) treated with ribbons (abbreviated here Rib, red) at 21 dpe. HSP-70 labeled the dense core of (C) somatic or (D) neuritic α-SynP inclusions. For each line the first two images represent single plane, whereas merge panels are composed of z stack projection. (E–G) (E and F) LAMP1 and (G and G′) LAMP2 immunostaining (green) largely colocalized with exogenous assemblies (ribbons, red) but did not show appreciable association with α-SynP structures (blue). Scale bars: 5 μm in (A–F and G′); 10 μm in (G).

The cores of the large somatodendritic α-SynP inclusions were positive for ubiquitin (Figure 4A) and strongly colocalized with SQSTM1-p62 (Figure 4B), an adaptor protein that delivers ubiquitinated cargoes to autophagy. In addition, the dense core of somatic inclusion as well as the neuritic filamentous inclusions was partially labeled by HSP-70 (Figures 4C and 4D), further indicating that endogenous aggregated α-Syn is targeted to degradation. We next assessed the association of exogenous assemblies and endogenous α-SynP inclusions with the lysosomal compartment in neurons. Exogenous α-Syn fibrils and ribbons largely colocalized (80% colocalization) with the lysosomal markers LAMP1 and LAMP2 at 21 dpe, indicating that the assemblies persist within the lysosomal compartment over several weeks. In contrast, α-SynP aggregates, including large filamentous, annular, or fragmented structures, were not found associated with LAMP1 and LAMP2 staining, which suggests that they are not within lysosomes/autophagosomes but within the cytosol. These structures resist degradation and accumulate in the cytoplasm of neurons. Altogether the variety of intra-neuronal inclusions we observed following exposure of human neurons to α-Syn fibrils and ribbons strikingly resembles, based on morphology, distribution, and colocalization with components of the protein degradation machinery, Lewy bodies and Lewy neurites found in the brain of PD patients (Goedert et al., 2013).

### Differential Seeding Properties of Ribbons and Fibrils: Dose- and Time-Dependence Analyses in Human Cortical Neurons

**Figure 5:**
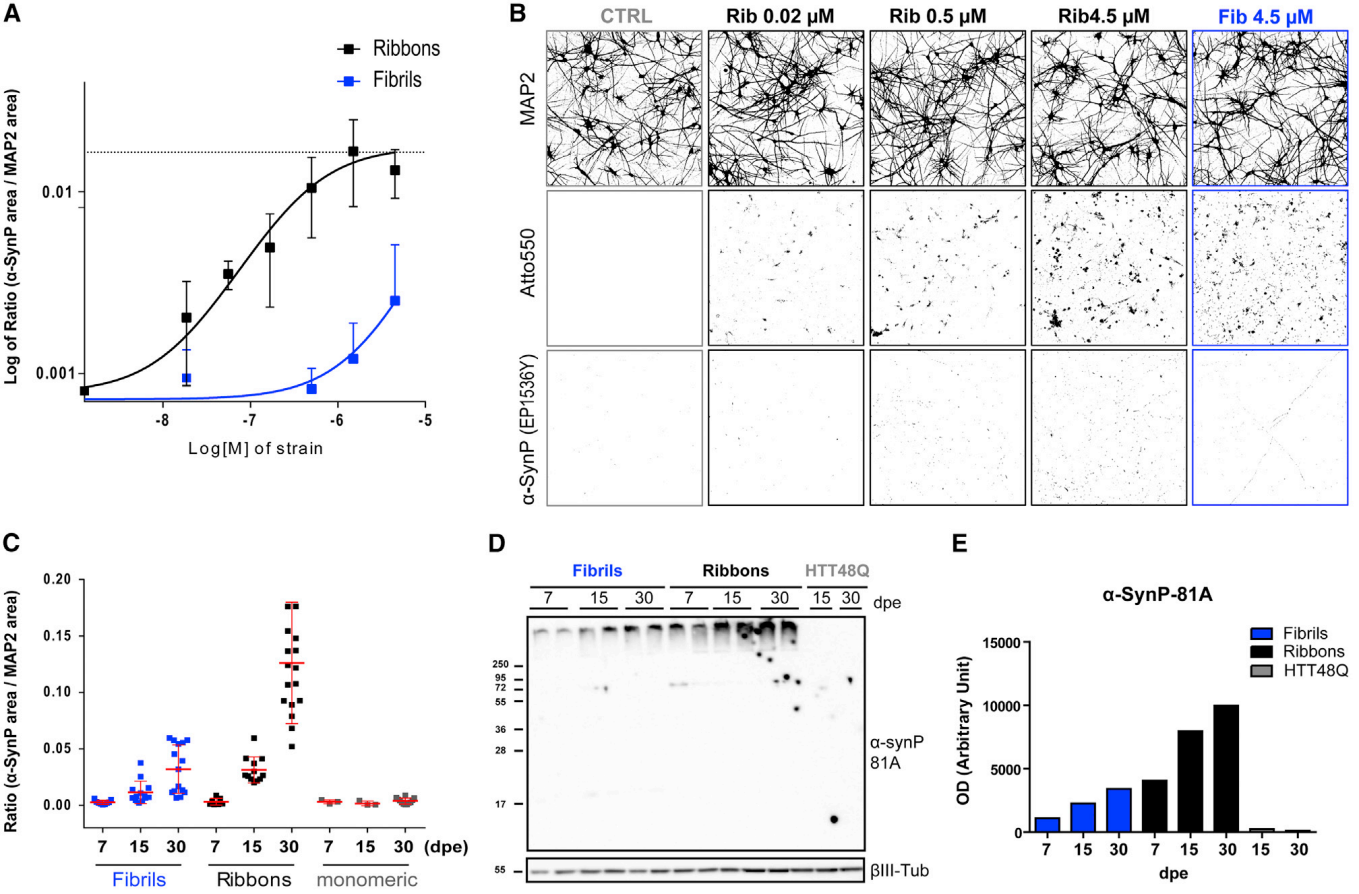
Dose- and Time-Dependent Effect of α-Syn Strain Exposure (A) Ratio (logarithmic scale) between the area occupied by α-SynP and MAP2 at increasing concentration of fibrils and ribbons (0.02 < x < 4.5 μM, 15 dpe; mean ± SD; n = 2). (B) Binary threshold masks of MAP2, Atto-550 assemblies, and α-SynP structures of control neurons (untreated, gray boxed) or neurons exposed at different concentrations of ribbons (black boxed) or fibrils (blue boxed). (C) Ratio between the area occupied by α-SynP and MAP2 over time (7, 15, and 30 dpe; [strains] = 500 nM). Data are shown as mean ± SD, n = 3 (for statistical analysis see Supplemental Information). (D and E) (D) Western blot analysis and (E) quantification showing the progressive accumulation of α-SynP in the insoluble fractions of neuron lysates at 7, 15, and 30 dpe to fibrils, ribbons, or fibrillar HTTExon1Q48 (α-SynP-81A antibody).

Using the ratio between the area occupied by α-SynP and MAP2 in neurons exposed to α-Syn strains as a readout of strain nucleation capacities, we performed dose-dependence analyses treating cells with up to 4.5 μM ribbons or fibrils. The data we obtained with ribbons at 15 dpe fitted a sigmoidal curve (Figures 5A and 5B) and allowed us to estimate a half-maximal effective concentration (EC_50_) (355 nM). For fibrils, the data fitted only partially a sigmoid curve and indicated an EC_50_ likely 2 log larger than that of ribbons. Interestingly, no significant changes in cortical neuron viability (pyknotic/normal DAPI-positive nuclei) or overall morphology (MAP2 total area per cell) were observed at any concentration of ribbons or fibrils we used (Figure 5B).

Next, we assessed the progressive accumulation of α-SynP in human cortical neurons exposed for 24 h to exogenous α-Syn fibrils and ribbons (0.5 μM) from 7 to 30 dpe (Figure 5C). The α-SynP/MAP2 ratio significantly increased with time, while it remained constant in neurons exposed to monomeric α-Syn. A higher α-SynP signal was measured upon exposure of neurons to ribbons compared with identical amounts of fibrils, in particular at the longest time point we analyzed (30 dpe, p < 0.001). These results were confirmed by biochemical analysis, where the amount of sarkosyl-insoluble α-SynP increased from 7 to 30 dpe for both strains (Figure 5D) and was 3-fold higher in cells exposed to ribbons compared with fibrils (Figure 5E). Altogether these experiments show that the exposure of WT human cortical neurons to α-Syn strains triggers the progressive conversion of homotypic neuronal α-Syn into insoluble and α-SynP aggregates and that this phenomenon depends on the strain conformation, with ribbons being more efficient than fibrils.

### Neuronal Dysfunctions Associated with Exposure to α-Syn Strains and α-SynP Accumulation

**Figure 6:**
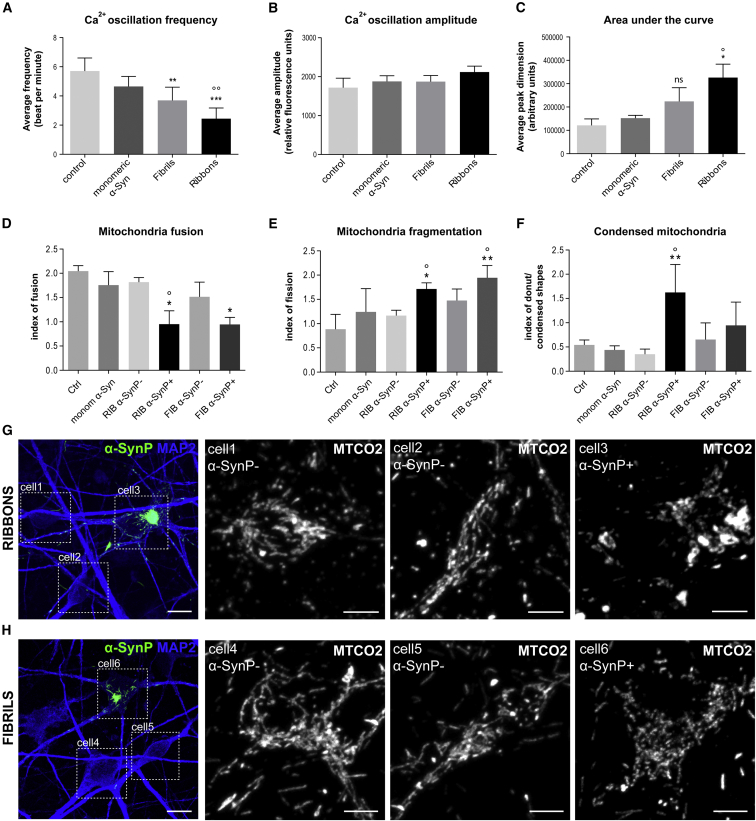
Alteration in Ca^2+^ Homeostasis and Morphological Impairment of Mitochondrial Networks in Human Neurons Following Exposure to α-Syn Strains and α-SynP Accumulation (A–F) (A–C) Whole-well Ca^2+^ imaging analyses on mature human cortical neurons 19 dpe to fibrils, ribbons, monomeric α-Syn, and untreated neurons. The (A) average frequency, (B) average amplitude, and (C) average area under the curve of the Ca^2+^ waves were analyzed for each well. Asterisks indicate significant difference of α-Syn strain-treated neurons versus control; circles indicate significant difference of α-Syn strain-treated neurons versus monomeric α-Syn. (D–F) Analysis of the index of (D) fusion, (E) fragmentation, and (F) donut-shaped/condensed mitochondria in untreated neurons or neurons at 21 dpe to monomeric α-Syn, ribbons, or fibrils. Asterisks indicate significant differences of treated neurons versus control; circles indicate significant differences of α-SynP^+^ versus α-SynP^−^ cells in the same culture. ^∗^ and °p < 0.05, ^∗∗^ and °°p < 0.01, ^∗∗∗^p < 0.001. Data are shown as mean ± SEM, n = 3 (for statistical analysis see Supplemental Information). (G and H) Representative confocal images of neurons exposed to (G) ribbons and (H) fibrils. On the left the cells are stained for MAP2 (blue) and α-SynP (green). Highlighted cells (dotted lines) are enlarged and stained with MTCO2 (white). Cells 1, 2, 4, and 5 are α-SynP^−^. Cell 3 and cell 6 are α-SynP^+^. Scale bars: 10 μm in left image of (G) and (H); 5 μm in cell insets.

We next investigated whether human neurons treated with α-Syn assemblies featured any type of early signs of neuronal dysfunction weeks after exposure to the strains despite the absence of evident morphological demise. We first performed Ca^2+^ imaging analysis on mature human cortical neurons exposed to ribbons, fibrils, and monomeric α-Syn. Synchronized spontaneous Ca^2+^ oscillations were recorded at 1 and 19 dpe using whole-well Fluo-4 calcium imaging (Figures 6A–6C and S5). All the parameters of these oscillations were comparable for untreated and monomeric α-Syn-treated neuronal cultures at 1 and 19 dpe. We detected minor alterations of average frequency and dimension (area under the curve, AUC) of Ca^2+^ waves at 1 dpe in α-Syn strain-treated neuronal cultures (Figures S5B and S5C). The extent of these alterations increases with time. At 19 dpe the frequency (Figures 6A and S5A) and the AUC (Figures 6C and S5A) were significantly altered for cultures exposed to ribbons (compared with untreated or monomeric α-Syn-treated cultures at 19 dpe), while the amplitude remained unchanged (Figure 6B); 19 dpe to fibrils triggered only a significant decrease in frequency (Figures 6A and S5A). The α-SynP/MAP2 ratio measured by immunofluorescence on cultures at 19 dpe and after Ca^2+^ analysis (same well analysis) showed nucleation levels similar to those presented in Figure 5C. Our observations indicate that neuronal cultures exposed to α-Syn strains present alterations in Ca^2+^ homeostasis that worsen over time in parallel with the progression in α-SynP accumulation (Figure S5B).

Several lines of evidence link mitochondrial dysfunction and α-Syn aggregation (Di Maio et al., 2016, Grassi et al., 2018, Prots et al., 2018, Reeve et al., 2015). We thus compared the morphology of mitochondria networks in control neurons and neurons exposed to ribbons, fibrils, and monomeric α-Syn (21 dpe). Neurons bearing somatic α-SynP inclusions induced by exposure to either ribbons or fibrils presented altered mitochondria morphologies. In particular, we observed significant reduction of tubular fused networks (Figures 6D, 6G, and 6H), increase in the proportion of fragmented mitochondria (Figures 6E, 6G, and 6H), and increase in the donut-shaped/condensed mitochondria (Figures 6F–6H). Control cells (unexposed or exposed to monomeric α-Syn) or cells lacking α-SynP inclusions from cultures exposed to exogenous α-Syn strains did not exhibit such alterations (Figures 6D–6F). These results demonstrate co-occurrence in human neurons of seeding events of endogenous α-Syn triggered by ribbons or fibrils and mitochondrial impairment.

### Phosphorylated α-Syn Propagation through Fluidically Isolated Human Cortical Neuronal Networks

**Figure 7:**
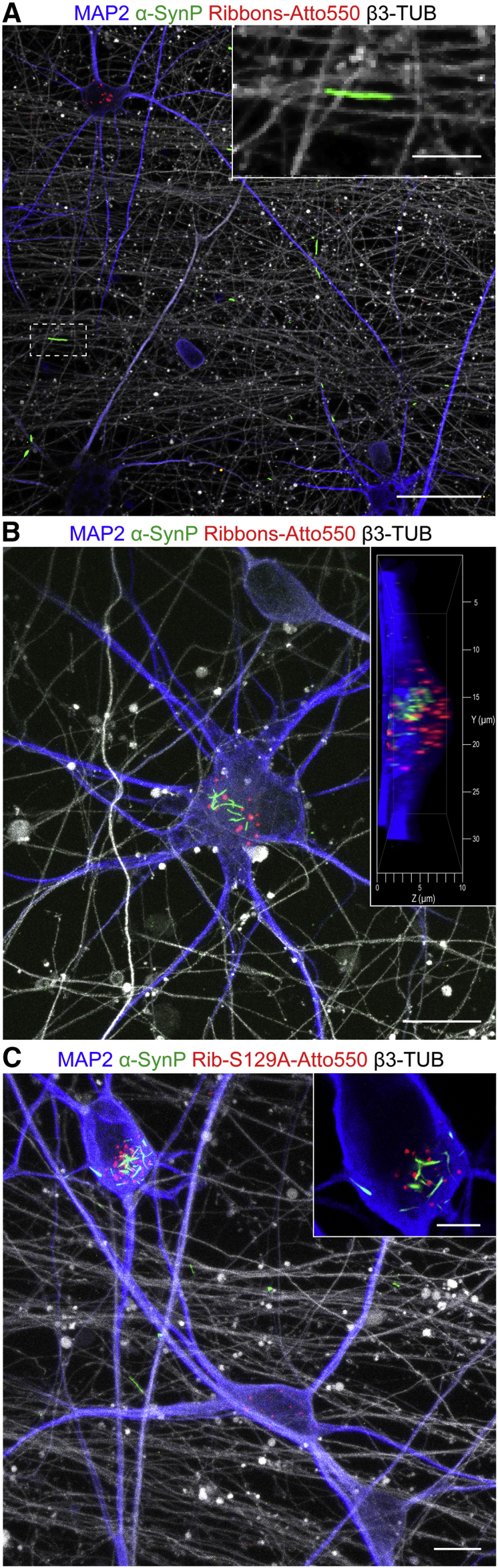
Induction of Trans-neuronal Nucleation Fluorescence confocal imaging illustrating endogenous α-SynP (green) in the distal chamber upon seeding of proximal neurons with exogenous α-Syn ribbons (made of WT in A and B, S129A α-Syn in C, red). Axons deriving from proximal neurons are identified using β3-tubulin (β3-TUB, gray), while neurons residing in the distal chamber are identified by MAP2 staining (blue). (A) α-SynP filiform structures are present in proximal neurons axons at 15 dpe. (B and C) At 21 dpe, α-SynP-positive clusters are detected as well in the cytoplasm of sparse distal neurons. Insets in (A) and (C) are enlargements of specific regions (highlighted with dotted lines if needed). Inset in (B) represents 3D reconstruction and orthogonal view of the intra-cellular α-SynP structures (Airyscan super resolution). Inset in (C) represents a single plane of the α-SynP cell. Scale bars: 40 μm in (A), 10 μm in (B) and inset in (A), 20 μm in (C), 5 μm in inset in (C).

Last we investigated whether exogenous α-Syn assemblies loaded only in the proximal chamber triggered, after spreading between neurons, the seeding of endogenous α-Syn in second-order neurons (i.e., located in the distal chamber). For this, neurons grown in the proximal chamber of corticocortical networks were challenged with α-Syn assemblies, either WT or S129A α-Syn ribbons, and the time course of α-SynP inclusions was monitored in the distal chamber. Starting from 15 dpe, α-SynP structures, mainly filiform shaped, were clearly detected in axons expanding from neurons residing in the emitting chamber (Figure 7A) but not in the unchallenged resident neurons. Three weeks after the exposure of proximal cells to ribbons made of either WT or S129A-α-Syn, α-SynP-positive clusters could be detected in the cytoplasm of a few second-order neurons in the distal chamber. The α-SynP structures ranged from small dots to more complex filiform architectures (Figures 7B and 7C), mainly detectable in the somatic compartment of second-order neurons of the distal chamber. Almost all the cells presenting α-SynP signal in the distal chamber also contained detectable exogenous aggregates, with very rare exception, thus suggesting that trans-neuronal nucleation was mainly due to initial seeds taken up by neurons in the proximal chamber that were subsequently transferred to second-order acceptor neurons.

## Discussion

Prion-like propagation of α-Syn aggregates (Brundin and Melki, 2017, Melki, 2015) is a scenario that could identify the sources of exogenous, non-genetic initiation of PD in patients and account for the sequential appearance of pathological hallmarks in different brain areas (Braak et al., 2003a, Jellinger, 2012). Here, we provide results that validate the prion-like propagation hypothesis of α-Syn aggregates at the human level. This is done in human neuronal cultures, made of cortical neurons and glial cells (>90% and <3% of cells, respectively), expressing genome-encoded WT α-Syn, as opposed to models where transgenic WT or mutant α-Syn is overexpressed (Luk et al., 2012b, Peelaerts et al., 2015).

We demonstrate that two different strains of human α-Syn (fibrils and ribbons) are transported, traffic between human neurons, and trigger the progressive accumulation of PD-like pathological hallmarks by recruiting the endogenous soluble α-Syn. We show that these assemblies act in a dose- and structure-dependent manner. The PD hallmarks they trigger in WT human cortical neurons, which include Lewy body- and Lewy neurite-like structures consisting of insoluble deposits of α-SynP, SQSTM1-p62, and HSP-70, correlate with early signs of neuronal dysfunctions such as alterations in calcium homeostasis and mitochondrial morphology.

We aimed at modeling α-Syn prion-like propagation in human corticocortical neuronal networks because it is affected at different stages of sporadic PD, DLB, and MSA (Braak et al., 2003a, Salvesen et al., 2017, Sugiura et al., 1995, Yau et al., 2018) and the conversion of endogenous WT α-Syn into pathological α-SynP aggregates may contribute to neuronal dysfunction in excitatory glutamatergic cortical neurons. Moreover, we chose to use hiPSCs from normal subjects specifically to model pathological processes involving α-Syn protein in idiopathic PD patients, by far the most numerous PD patients.

Our observation that α-SynP aggregates accumulate progressively over 30 days in neuronal axons and somatodendritic compartments suggests that α-Syn strains trigger sustained nucleation of host-encoded neuronal α-Syn. The fact that fluorescently labeled exogenous α-Syn strains do not overlap with α-SynP foci also shows that exogenous α-Syn assemblies not only are not phosphorylated, in agreement with previous findings (Pieri et al., 2016), but trigger endogenous α-Syn aggregation, with subsequent massive phosphorylation of the latter. To further strengthen this set of converging evidence, we used not only different anti-α-SynP antibodies (Rutherford et al., 2016, Sacino et al., 2016, Uchihara and Giasson, 2016) but also strains made of variant α-SynP (α-Syn S129A) that cannot be phosphorylated on residue S129, and thus are undetectable with anti-phospho-S129 α-Syn antibodies. Our data unambiguously show that (1) exogenous α-Syn strains trigger the progressive accumulation of PD-related hallmarks in human WT neurons by progressive corruption of the endogenous α-Syn and (2) this phenomenon is not prevented by mutations that compromise phosphorylation of exogenous seeds on this specific site (Azeredo da Silveira et al., 2009, Tenreiro et al., 2014). Furthermore, we showed that large cytosolic α-SynP somatic inclusions bear marks of cellular recognition as aggregated proteins and are targeted to autophagy as demonstrated by colocalization with ubiquitin, SQSTM1-p62, and HSP-70. Interestingly, this was observed only in neurons under our experimental conditions. Indeed, while rare astrocytes present in the cell culture chambers displayed internalized fluorescent α-Syn assemblies, we were never able to detect α-SynP inclusion in these cells. This is due to either lower seeding efficiency of exogenous α-Syn strains or higher clearance efficiency of aggregated α-Syn in astrocytes (Loria et al., 2017). Exogenous assemblies were rapidly internalized by most of the directly exposed neurons within the proximal chamber and transported along their axons to cells residing in the distal chamber. We confirmed that the direct uptake of extracellular aggregates from the medium as well as the inter-neuronal transfer from acceptor to donor neurons mainly relies on endocytic processes (Apetri et al., 2016, Flavin et al., 2017, Lee et al., 2008, Sacino et al., 2016) (Brahic et al., 2016, Freundt et al., 2012).

Both α-Syn strain assemblies were transported with similar axonal velocities. They also propagated between neurons at similar rates. This is interesting and may suggest that α-Syn strains are transported by a given vehicle/machinery as opposed to other pathogenic protein assemblies that are transported and exported at different rates (Brahic et al., 2016). We report that both α-Syn strains promoted the formation of α-SynP structures resembling Lewy bodies and Lewy neurites. Nonetheless, ribbons were consistently more potent in inducing the accumulation of pathological α-SynP in human WT neurons. This is in agreement with observations we and others made in mouse (Peelaerts et al., 2015, Peng et al., 2018) and likely reflects a higher seeding propensity. Phenotypically distinct PrP-derived prion strains (scrapie, Creutzfeldt-Jakob disease) are discriminated using several parameters, among which is the seeding propensity. While slow versus fast propagation phenotypic traits are associated with cellular PrP expression level, they are also linked to differences in biochemical properties of PrP aggregates or in their cell targeting properties (Le Dur et al., 2017). In that regard, we consider that ribbons and fibrils do harbor a distinct strain phenotype that is reminiscent of fast versus slow propagation properties.

In the absence of major morphological alteration or neuronal loss upon exposure to exogenous α-Syn strains we assessed early signs of neuronal dysfunctions. We observed alterations in Ca^2+^ homeostasis in human neuronal networks exposed to α-Syn strains reminiscent of synaptic failure. These alterations paralleled the progression in α-SynP accumulation. These results are coherent with recent observations reporting functional defect in mouse hippocampal (Froula et al., 2018) and pyramidal (Kaufmann et al., 2016) neurons exposed to the fibrillar form of α-Syn. Moreover, focusing on neurons with large somatic inclusions, we observed a strong correlation between α-Syn seeding and mitochondria morphological impairment. A relationship between mitochondrial dysfunction and α-Syn aggregation was recently and independently evidenced (Di Maio et al., 2016, Grassi et al., 2018, Prots et al., 2018, Reeve et al., 2015). Our findings, in a relevant human cellular model, further highlight a role for α-Syn seeded aggregation in the development of functional defects likely initiating neurotoxicity associated with PD.

Finally, taking advantage of our microfluidic setup, we were able to evidence seeding of endogenous α-Syn in naive acceptor neurons after transfer of assemblies from donor neurons exposed to exogenous α-Syn seeds. This shows that prion-like propagation in neuronal networks comprises at minimum two distinct components, trans-neuronal spreading and intra-cellular seeding, the latter being the rate-limiting factor for amplification. Most importantly our results indicate that pathological forms of α-Syn actively disseminate and amplify in WT human neuronal networks by recruiting soluble α-Syn, a key step in the transmission of α-Syn pathogenic assemblies between human neurons in PD and related synucleinopathies.

The “Human Brain-on-Chips” functional platform we set up is uniquely suited to further assessing the prion-like spreading of aggregated α-Syn and its functional impact on neurons in distinct synucleinopathies. Because it is based on human neurons, expressing physiological levels of WT α-Syn, and preserves some degree of anatomical relevance, our *in vitro* model of a human neuronal network greatly extends the throughput at which many, if not all, aspects of prion-like spreading can be studied. A key advantage of our model is, in addition, that it bypasses the inter-species intrinsic differences in age-related neurodegenerative phenomena between rodents and humans. We are confident that such system will prove important as a predictive preclinical model to decipher the different components of the prion-like propagation (internalization, transport, nucleation) of α-Syn and of other protein aggregates in distinct neuropathological disorders and most importantly to later assess the efficiency of disease-modifying therapeutic approaches targeting key components of such pathways.

## Experimental Procedures

### Generation of Neural Progenitors from Human Induced Pluripotent Stem Cells

hiPSC (i90c17 and 16, passage 30–50; Nicoleau et al., 2013) amplification and neural progenitor cell generation, differentiation, and immunostaining were performed as described in Supplemental Experimental Procedures.

### α-Syn and HTTExon1Q48 Purification and Aggregation into Assemblies

Purification and quality control of human recombinant monomeric WT or S129A α-Syn and assembly into fibrils and ribbons and purification and assembly into fibrils of human recombinant HTTExon1Q48 were carried out as previously reported (Bousset et al., 2013) and described in Supplemental Experimental Procedures.

### Biochemical Analysis

Cells cultured in classical 24-well plates were scraped into lysis buffer (20 mM Tris-HCl, pH 7.5; 0.8 M NaCl; 1 mM EGTA; 10% [w/v] sucrose; 1% sarkosyl) supplemented with protease (Roche) and phosphatase (Sigma) inhibitor cocktails and solubilized at 37°C for 30 min. Filter trap and insolubility assay were performed as previously reported (Bousset et al., 2013) and described in Supplemental Experimental Procedures.

## Author Contributions

S.G. completed hiPSC neuronal differentiation, microfluidic network reconstructions, α-Syn propagation assays, confocal analysis, and Ca^2+^ imaging analysis. P.T. performed microfluidic network reconstructions, α-Syn propagation assays, and biochemical analysis. L.B. produced and characterized α-Syn assemblies and performed biochemical analysis. A.F. performed quality control of assemblies. P.L. contributed to sample preparation. R.M. supervised the biochemical works. A.P. supervised the iPSC assay development. J-M.P. supervised the network reconstruction assay and α-Syn propagation experiments. S.G., P.T., R.M., J-M.P., and A.P, wrote the manuscript.

## Supplemental Information

Document S1. Supplemental Experimental Procedures, Figures S1–S5, and Table S1

Document S2. Article plus Supplemental Information
