# Supplementary material for: Propagation of α-Synuclein Strains within Human Reconstructed Neuronal Network
Source: Stem Cell Reports. 2019 Jan 10;12(2):230–44. doi: 10.1016/j.stemcr.2018.12.007 (PMC6372945; doi:10.1016/j.stemcr.2018.12.007)
Supplement: Document S1. Supplemental Experimental Procedures, Figures S1–S5, and Table S1 [file mmc1.pdf]

Stem Cell Reports, Volume *12*

Supplemental Information

Propagation of α-Synuclein Strains within Human Reconstructed Neuronal Network

Simona Gribaudo, Philippe Tixador, Luc Bousset, Alexis Fenyi, Patricia Lino, Ronald Melki, Jean-Michel Peyrin, and Anselme L. Perrier

Supplemental information

SUPPLEMENTAL ITEMS

Supplemental video 1 and 2. *Axonal Transport of α-Syn ribbons (video1) or fibrils (video2) visualized at the exit of the microgrooves in the distal chamber*, Related to Figure 2. Thirty days in vitro reconstructed human cortical neuronal networks were loaded in the proximal chamber with 500 nM Atto-550 labelled ribbons or fibrils. The movements of the fluorescent assemblies were then imaged in the distal chamber, at the exit of the microgrooves, 24h after exposure. Fluorescent images were acquired every 600 ms for 5 min and were superimposed on a phase-contrast image acquired at the beginning of the acquisition (scale bar: 50 µm; timestamp: sec).

SUPPLEMENTAL FIGURES

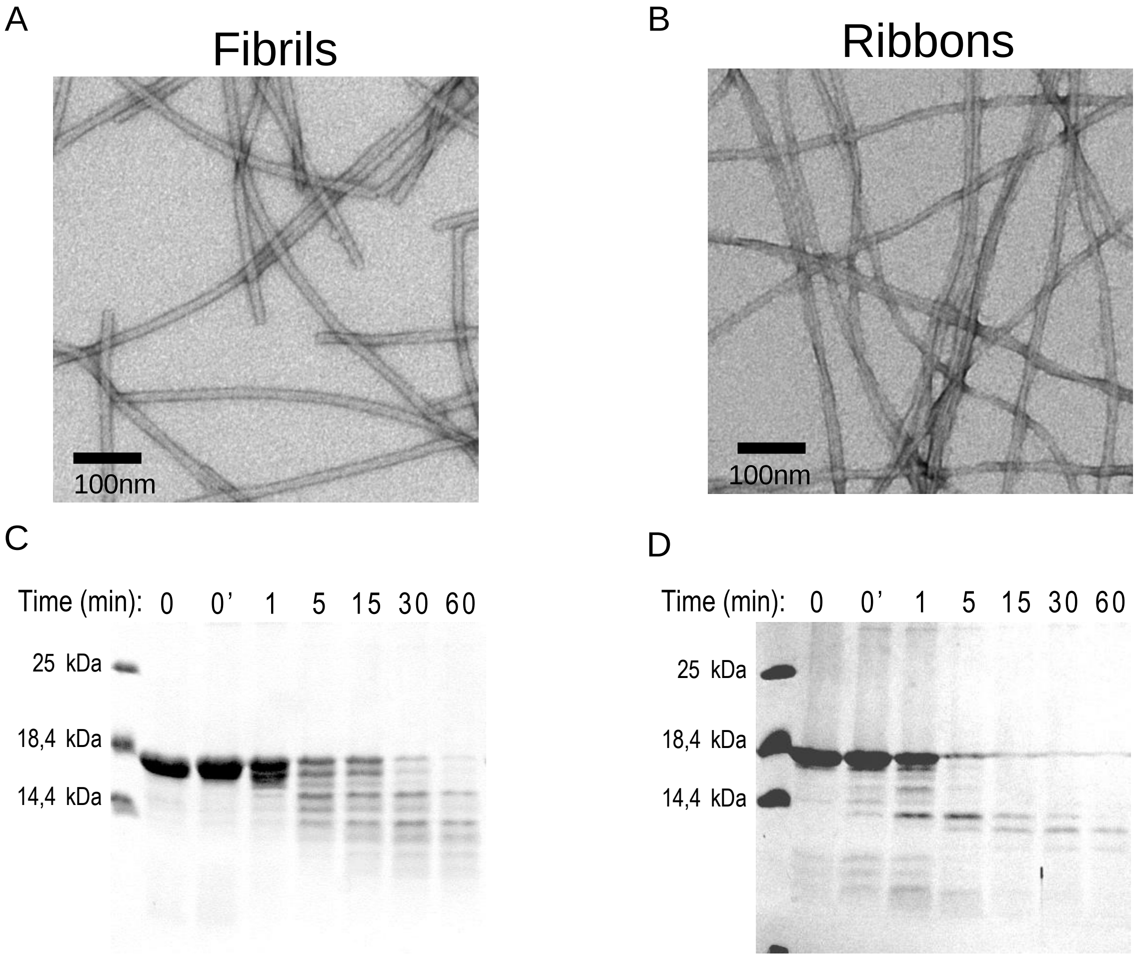

Figure S1. *Quality control and characterization of fibrils and ribbons*, Related to Figure 2-7. Fibrils and ribbons were generated exactly as described in Bousset et al., 2013 and as described in the materials and methods section. The resulting assemblies were adsorbed on 200 mesh carbon coated electron microscopy grids and imaged by transmission electron microscopy after negative staining (A and B). Besides differing through their shapes, the polymorphs/strains fibrils (cylindrical) and ribbons (flat) differ through their limited proteolytic patterns. Thus, the resulting fibrils (C) and ribbons (D) (mg/ml) were subjected to degradation by proteinase K. Aliquots were removed before (0), immediately (0') or at the indicated time (in minutes) after addition of proteinase K, denatured in boiling Laemmli buffer for 5 minutes at 90°C and subjected to SDS-PAGE on 12% polyacrylamide gels. The Coomassie stains of the SDS-PAGE are shown (C and D). The patterns in C and D are

characteristic of the polymorphs/strains fibrils and ribbons, respectively (Bousset et al., 2013). The pre-stained molecular weight markers are shown on the left.

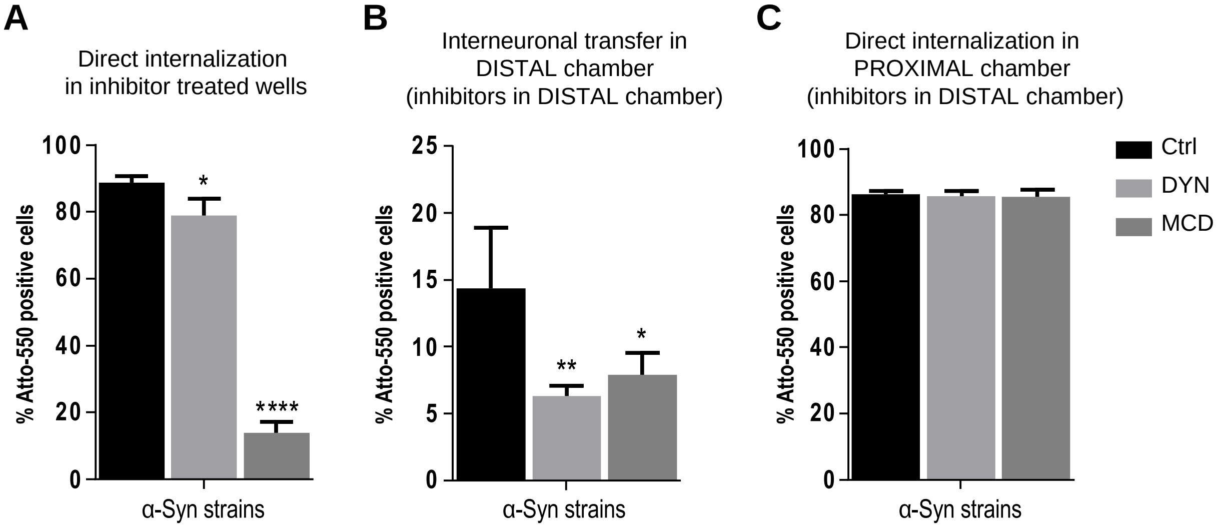

Figure S2. *Inhibition of receptor/clathrin-dependent endocytosis or fission of endocytic vesicles reduces the direct and indirect internalization of α-Syn strains,* Related to Figure 2. A-C: Before strains addition, neurons were pre-treated for 1 hour with Monocadaverin (MCD, 70 µM) or Dynasore (DYN, 80 µM) and analysis were performed 24 hours later. A: The direct uptake of Atto-550 α-Syn strains (fibrils and ribbons) from the medium was strongly reduced by application of MCD while application of DYN only partially reduced the uptake of the assemblies. B-C: taking advantage of the microfluidic system, the effect of MCD and DYN was tested on the capacity of secondary neurons located in the distal chamber to uptake Atto-550 strains from axon of “donor” neurons. B: Both MCD and DYN application reduced the proportion of neurons in the distal chamber with internalized exogenous assemblies. C: the proportion of Atto-positive neurons in the proximal chamber did not change during inhibitors application in the distal chamber (F=473.6 in A, F=8.10 in B, ANOVA with Dunnett’s post-hoc tests). * represents $p < 0.05$, ** and °° $p<0.01$, **** $p < 0.0001$. Data are mean ± SEM, n=3 independent experiments.

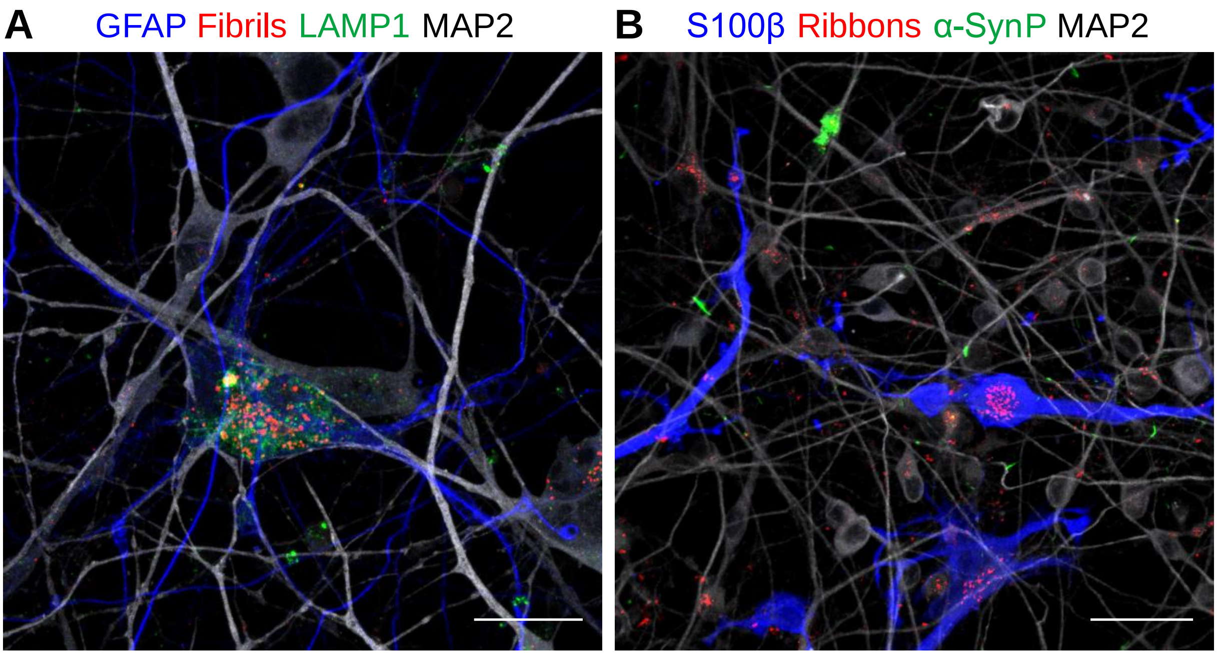

Figure S3. *Immunofluorescence analysis of human glial cells exposed to α-Syn assemblies*, Related to Figure 3. A: Fluorescence confocal imaging of LAMP1 (green) in human cortical culture (MAP2, grey) 30 days after exposure to 500 nM Atto-550 labelled (red) α-Syn strains. Representative images showing that Atto-550 assemblies in lysosome (LAMP1+) of GFAP+ (blue) glial cells. B: Fluorescence confocal imaging of endogenous α-SynP (green) in human cortical culture (MAP2, grey) 30 days after exposure to 500 nM Atto-550 labelled (red) α-Syn ribbons. Representative images showing that α-SynP inclusions are not found in S100β-positive (blue) glial cells. Scale bar 20 µm in A, 40 µm in B.

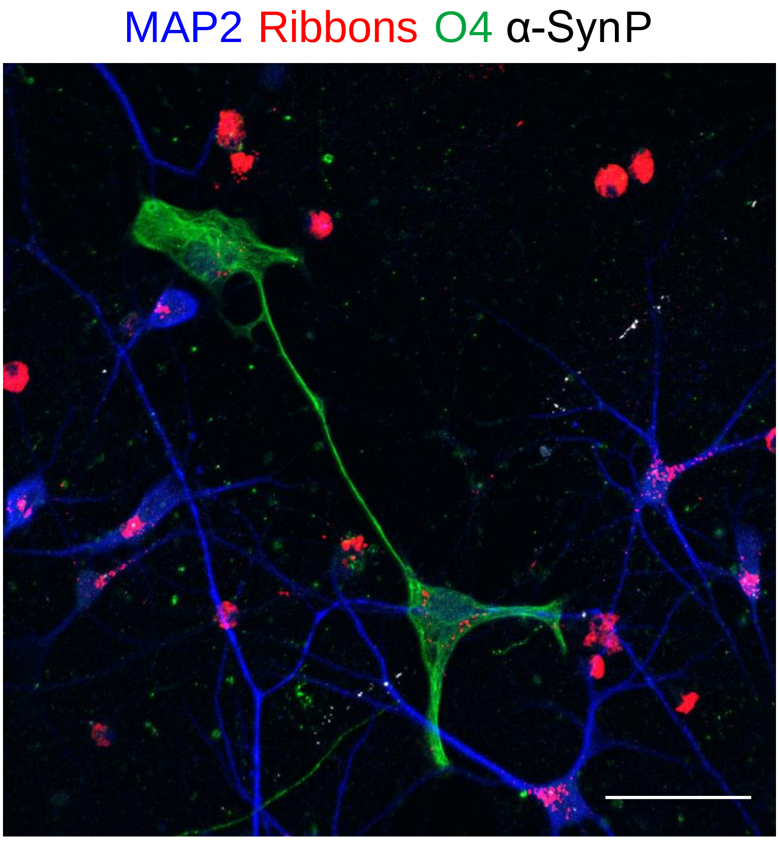

Figure S4. *Immunofluorescence analysis of human neuronal cultures containing O4-positive oligodendrocytes precursor cells exposed to α-Syn assemblies,* Related to Figure 3. Fluorescence confocal imaging of O4 (green) in human cortical culture (MAP2, blue) 21 days after exposure to 500 nM Atto-550 labelled (red) α-Syn fibrillar assemblies. Representative images showing the presence of Atto-550 assemblies in O4 positive cells; α-SynP (grey) inclusions are never found in O4-positive (green) cells. Scale bar 40 µm

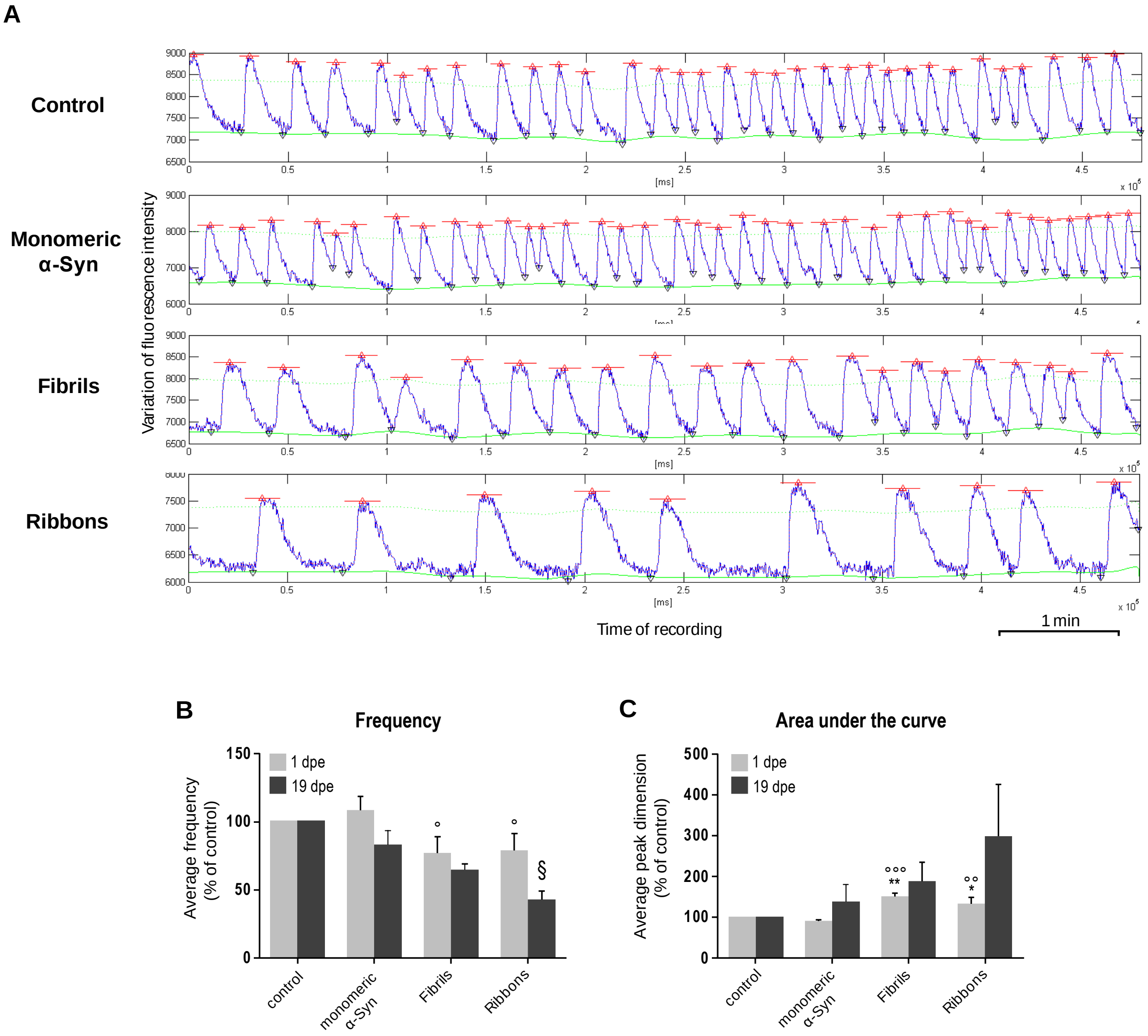

Figure S5. *Alterations of* Ca^{2+} *oscillations in human neuronal cultures treated with exogenous α-Syn assemblies*, Related to Figure 6. A: Representatives traces of spontaneous Ca^{2+} oscillations obtained by whole well recording followed by FDSS waveform analysis of Fluo4 loaded cortical human neuronal cultures. Cells were treated at 5 weeks and Ca^{2+} recording was performed 19 days post exposure (dpe) to monomeric α-Syn, fibrils, and ribbons. Control cells were unexposed. B-C: Average frequency (B) and average peak dimension (C, area under the curve) in mature neuronal networks (recording performed at 8 weeks) at 1 or 19 dpe. Already at 1 dpe, ribbons and fibrils exposure triggered decrease in frequency (B) and increase in the AUC (C) of Ca^{2+} oscillation in human neuronal cultures (F=8.36 in B, F=28.65 in C, ANOVA with Tukey's post-hoc tests). Stars indicate significant difference of 1 dpe treated neurons vs control; empty dots indicate significant difference of 1 dpe treated neurons vs monomeric α-Syn. * and ° represent $p< 0.05$, ** and °° $p<0.01$, °°° $p < 0.001$. These variations tend to amplify with time. In particular decrease in peak frequency between 1 and 19 dpe (B) is significant in ribbons-treated neuronal cultures (F=8.113, p=0.0015, ANOVA with Sidak's post-hoc test, indicated by the paragraph sign). Data are presented as mean percentage compared to control ± SEM, n=3 independent experiments for each exposure time.

SUPPLEMENTAL EXPERIMENTAL PROCEDURES

Microfluidic chips fabrication

Three compartmented microfluidic chambers were designed and produced as previously described (Deleglise et al., 2013). After bonding PDMS chips to glass coverslip with a plasma generator, the chips were coated with a solution of poly-Ornithine overnight (0.002% final concentration, P4957 Sigma; St. Louis, MO, USA). Chips were then washed in PBS and further coated with Laminin (4µg/ml final concentration, 23017015, Invitrogen) for at least 4 hours.

Neural induction and differentiation of hiPSCs

The i90c17 and 16 human iPS cells line (passage 30-50 (Nicoleau et al., 2013)) were maintained on L7 (Lonza) matrix and grown in STEMPRO medium (Invitrogen) in presence of FGF2 (10 ng/ml, Peprotech). Cultures were fed daily and manually passaged every 4–5 days. For neural differentiation, hiPSC colonies were treated as previously described (Nicoleau et al., 2013). N2B27 medium was supplemented with 0.1 µM LDN-193189 (Sigma), 20 µM SB-431542 (Tocris), 1 µM XAV-939 (Tocris) for the first 10 days and with 1 µM XAV-939 only starting from day 10. At day 18, cells were enzymatically dissociated using Accutase (Invitrogen) and further expanded in N2B27 supplemented with FGF2 (20ng/ml), EGF (10 ng/ml, R&D systems) and BDNF (10 ng/ml Peprotech). Cells were passaged up to 6 times every 3-4 days. For banking, neuronal precursor (NSC) were resuspended at $8x10^6$ cells/ml in "freezing medium" (90% fetal calf serum 10% DMSO), frozen and stored in liquid nitrogen vapor at -150°C.

Neuronal differentiation of hiPSC-derived NSCs

NSC were suspended in N2B27 medium to a final density of $80x10^6$ cells/mL for the proximal compartment and $20x10^6$ cells/ml for the distal compartment into poly-L-ornithine/laminin coated microfluidic chips or at 10^6 cells/ml for culture in regular culture plate. For the final differentiation cells were cultivated in presence of BDNF (20 ng/ml), cAMP (100 µM, Sigma) and valproic acid (0.5 mM, Sigma). DAPT (5 µM, Tocris) was added to the culture for the first 2 weeks. Medium was change every 5 days.

Immunofluorescence

Cells were fixed with 4% PFA/4% sucrose and further permeabilized with 0.1% Triton X-100 and 2% BSA in PBS. To assess α-Syn detergent insolubility by immunofluorescence, samples were alternatively fixed with 4% PFA in presence of 1% Triton X-100 as previously described (Volpicelli-Daley et al., 2016). Primary antibodies (listed in Table S1) were then added and the samples incubated at 4°C overnight in PBS. Species specific secondary antibodies coupled to Alexa 350, 488, 555 and 647 (1/1000, Invitrogen) and DAPI counterstain were applied for 1 hour at room temperature.

Image acquisition and analysis

Confocal analyses were performed using a ZEISS LSM 880 Confocal Laser Scanning Microscope with Airyscan module (Zen software). Quantitative and live imaging analyses were acquired with an Axio-observer Z1 (Zeiss) fitted with a cooled CCD camera (ORCA-Flash4, Hamamatsu, Coolsnap HQ2, Roper Scientific, Metamorph software (Molecular Imaging)) for widefield fluorescence acquisition or with an Evolve EMCCD camera (Zeiss), coupled to a spinning disk system (Nipkow, CSU-X1M 5000, Zeiss). Quantitative immunofluorescence analysis was performed on randomly selected visual fields from three independent microfluidic devices using ImageJ. On average, 3 visual fields were acquired per each microfluidic chamber and a total of 50 cells were counted per field. Data are presented as mean ± SEM. The quantification of α-SynP in neurons was estimated by the ratio between the area occupied by α-SynP and MAP2 in the somatic and proximal dendrites compartments of neurons exposed to either fibrils or ribbons. Data are presented as mean ± SD.

α-Syn and HTTExon 1Q48 purification and aggregation into assemblies

A variant human α-Syn where Serine 129 residue was changed to Alanine (S129A α-Syn) was generated by site directed mutagenesis. This variant cannot be phosphorylated in neurons on S129, the main phosphorylation site for α-Syn. Purification and quality control of human recombinant monomeric WT or S129A α-Syn and assembly into fibrils and ribbons were carried out as previously described (Bousset et al., 2013). The purification and

assembly into fibrils of human recombinant HTTExon1Q48 was performed as described (Monsellier et al., 2015). WT and S129A α-Syn fibrils, ribbons and HTTExon1Q48, preformed fibrils were pelleted and washed with PBS buffer, pH 7.4 and further labelled with a. two molar excess of NHS-ester ATTO 550 (AttoTec) according to manufacturer's recommendations. Preformed assemblies were fragmented by sonication for 20 min in 2-ml Eppendorf tubes in a Vial Tweeter powered by an ultrasonic processor UIS250v (250 W, 2.4 kHz; Hielscher Ultrasonic, Teltow, Germany). Following labelling, the unreacted dye was removed using a desalting column (PD10, GE Healthcare) equilibrated in PBS buffer, pH 7,4.
For transmission electron microscopy, the assemblies were adsorbed on 200 mesh carbon coated electron microscopy grids and imaged after negative staining. For degradation by proteinase K, aliquots of fibrils and ribbons (mg/ml) were removed before (0), immediately (0') or at the indicated time (in minutes) after addition of proteinase K, denatured in boiling Laemmli buffer for 5 minutes at 90°C, subjected to SDS-PAGE on 12% polyacrylamide gels and stained by Comassie coloration.

Exposure of human neuronal cells to monomeric α-Syn, α-Syn strains and fibrillar HTTExon1Q48

Thirty days after seeding, neuronal cultures in plates or microfluidics were exposed to 500 nM of Atto550 labelled α-Syn fibrils, ribbons and monomers and to fibrillar HTTExon1Q48 with a 24 hours pulse, and then washed. The occurrence of de novo formation of pathological α-Syn was then assessed by biochemical and immunofluorescence approaches at 7, 15 and 30 days post-exposure to the different samples. For longitudinal analysis of exogenous assemblies transport/transfer, proximal chambers were loaded with 500 nM of Atto550 labelled α-Syn fibrils, ribbons and monomers with a 24 hours pulse. To prevent passive diffusion of assemblies, hydrostatic pressure was applied by adding medium excess in the reservoirs of recipient chambers. Loaded networks were imaged 1, 7, 15 and 21 days after addition of assemblies. To analyze axonal transport, time lapse imaging (5 minutes films, with time interval of 600 ms) was performed 24 hours post loading with an inverted Zeiss spinning disk system. Enhanced projections of the video sequences to identify and track axonal/fluorescent punctae paths were performed. For each path/axon segment, a kymograph representation was extracted with ImageJ software plug-In "Multiple Kymograph" and average velocity pattern as well as average path lengths of fluorescent punctae were then determined.

Analysis of endocytosis inhibitors

The contribution of receptor/clathrin-dependent endocytosis (Schlegel et al., 1982) or fission of endocytic vesicles (Macia et al., 2006) to the direct uptake of α-Syn strains by human cortical neurons was assessed. To this end, cells in classic p24 vessel were pretreated for 1 hour with 70 µM monodansylcadaverine (Sigma-Aldrich) or 80 µM dynasore (Tocris Bioscience Bristol, UK) and imaged 24 h after their exposure to Atto-550 Ribbons or Fibrils, at 500 nM, after washing. To analyze the effect of those two pharmacological inhibitors on the interneuronal transfer of fibrils and ribbons, our microfluidic setup was used. The neurons resident in the distal chamber (acceptor neurons) were pretreated with monodansylcadaverine or dynasore for 1 hour before exposing the cells in the proximal chamber (donor neurons) to 0.8 µM of fibrils or ribbon, in order to maximize the % of spreading between the neuronal networks. The cells in the 2 chamber were imaged 24 hours later.

Biochemical Analysis

Cells cultured in classic 24 well plates were scraped into lysis buffer (LB; 20mM Tris-HCl pH 7.5; 0,8M NaCl; 1mM EGTA; 10% (w/v) sucrose; 1% sarkosyl) supplemented with protease (Roche) and phosphatase (Sigma) inhibitor cocktails and solubilized at 37°C for 30 min. Lysates were then precleared by centrifugation at 1000 g for 10 min and the supernatants were retained for further analyses. Filter trap and insolubility assay were performed as previously described (Bousset et al., 2013). The primary antibodies used are listed in Table 1. After 4 washes with TBS-T, the membranes were further incubated for 1h with appropriate HRP conjugated secondary antibody diluted (1: 2000) in blocking buffer and then washed 4 times with TBS-T. Immunoblots were revealed with West Femto ECL (Pierce). Chemiluminescence was recorded on LAS-3000 imaging system (FUJIFILM) or Chemidoc (Bio-Rad) and quantified with image J.

Ca2+ imaging analysis

Human cortical neurons were cultured in 384 well plates at a density of 80.000 cells per cm^2. For Ca^{2+} imaging experiments, cells were loaded for 15 minutes with Fluo4 probe (1 µM, Invitrogen) in HBSS physiological solution then washed 3 times. For both 1 and 19 dpe studies, Ca^{2+} imaging recording were performed on human neuronal cultures at 8 weeks of final differentiation (5 weeks + 19 days, or 8 weeks + 1 day). Spontaneous Ca^{2+} oscillations were recorded using the FDSS 600 Hamamatsu imaging-based plate reader, for duration of 8

minutes at a time interval of 0.4 seconds. Analyses of the frequency, amplitude and area under the curve of the Ca^{2+} oscillations for each well were performed using the FDSS Waveform analysis software.

Analysis of mitochondria morphologies

For mitochondria morphological analysis, neuronal culture in µfluidic devices were treated at 4 weeks of differentiation and analyzed 21 days after exposure to fibrils, ribbons or monomeric α-Syn. Images were acquired by confocal microscope. For ribbons and fibrils treated neurons, cells were selected based on the presence of α-SynP somatic inclusion (α-SynP+ cells). The α-SynP– cells belonging to the same culture and surrounding the α-SynP+ neurons (e.g. not presenting α-SynP somatic inclusion but loaded with Atto-550 fibrils or ribbons) were analyzed separately. For unexposed neurons or neurons exposed to monomeric α-Syn, cells were acquired randomly. For the morphological analysis, for each cell (96 for RIB, 84 for FIB, 90 for monomeric and 65 for ctrl, from n=3 independent experiments) a score was assigned based on the MTCO2 staining, MAP2 used to identify the neurons. The score range from 0 to 3 for each category that include i) fusion, ii) fragmented/fission and iii) donut-shaped/condensed mitochondria. Classified cells were subsequently evaluated on α-SynP staining, and further distinguished in α-SynP+ or α-SynP-.

Statistical analyses

Figure 2A and 2B, *Interneuronal spreading of α-Syn assemblies*:

Two-way ANOVA: for proximal chamber (Fig 2A), F=40.50 (interaction), F=489.7 (treatment); for distal chamber (Fig 2B), F=6.237 (interaction), F=97.74 (treatment). Data are presented as mean ± SD, n=4 independent experiments. Multiple pairs of conditions show significantly different % of Atto-550 positive cells in proximal and distal chamber (Tukey's post-hoc tests):

Distal chamber (2B):

- Time effect: fibrils 1 vs 15 and 21 dpe ($p<0.0001$), 7 vs 15 and 21 ($p<0.001$); ribbons 1 vs 7 ($p<0.01$), 1 vs 15 and 21 ($p<0.0001$), 7 vs 15 and 21 ($p<0.0001$).
- Strains effect: fibrils and ribbons vs monomeric α-Syn at 1 dpe ($p<0.01$), 7, 15 and 21 dpe ($p<0.0001$); fibrils vs ribbons never significant.

Proximal chamber (2A):

- Time effect: monomeric α-Syn 1 vs 7, 15, 21 ($p<0.0001$), 7 vs 15 ($p<0.5$) and 21 ($p<0.01$); fibrils never significant; ribbons 1 vs 15 and 21 ($p<0.01$), 7 vs 15 and 21 ($p<0.01$).
- Strains effect: fibrils and ribbons vs monomeric α-Syn at 1, 7, 15, 21 dpe ($p<0.001$); fibrils vs ribbons never significant.

Figure 5C, *Time dependent effect of α-Syn strains exposure*:

Two-way ANOVA, F=16.55 (interaction), F=24.98 (treatment). Data are presented as mean ± SD, n=3 independent experiments. Multiple pairs of conditions show significant increase of the ratio between α-SynP area/MAP2 area over time for the 2 strains (Tukey's post-hoc tests):

- Time effect: fibrils 7 dpe vs 30 dpe ($p<0.05$); ribbons 7 or 15 dpe vs 30 dpe ($p<0.001$).
- Strain's effect (at 30 dpe): fibrils vs control ($p<0.05$), ribbons vs control ($p<0.001$), ribbons vs fibrils ($p<0.001$).

Figure 6A-C, *Alteration in calcium homeostasis:*

ANOVA, F=23.83 in A, F=7.14 in C, with Tukey's post-hoc tests. Stars indicate significant difference of α-Syn strains treated neurons vs control; empty dots indicate significant difference of α-Syn strains treated neurons vs monomeric α-Syn. Data are presented as mean ± SEM, 4 wells per experiment, n=3 independent experiments.

Figure 6D-E, *Alteration in mitochondria morphologies:*

For each cell (96 for RIB, 84 for FIB, 90 for monomeric and 65 for ctrl, from n=3 independent experiments, data are presented as mean ± SEM), a score was assigned (from 0 to 3) to each category and the cells were subsequently classified as α-SynP+ or α-SynP-.

- Comparison of treated neurons vs control: ANOVA, F=4.47 in D, F= 5.64 in E, F=5.60 in F, with Dunnett's post-hoc tests; differences are indicated by stars.
- Comparison of α-SynP+ vs α-SynP- cells present in the same culture: ANOVA, F=3.80 in D, F= 8.86 in E, F=5.11 in F, with Sidak's post-hoc tests; differences are indicated by empty dots.

Table S1. *List of primary antibodies*, Related to Figure 1-7.

Primary antibodies	Dilution IF	Dilution WB	Species	Provider	Reference number
**BIII-TUB**	1/1000		ms monoclonal	BioLegend	MMS-435P
**BIII-TUB**	1/1000		chick polyclonal	Aves Labs	---
**BRN2**	1/800		goat monoclonal	Santa Cruz Biotechnology	sc-6029
**CTIP2**	1/500		rat monoclonal	abcam	ab18465
**CUX2**	1/2000		rb polyclonal	abcam	ab130395
**GABA**	1/1000		rb polyclonal	Sigma	A2052
**GFAP**	1/5000		rb polyclonal	DakoCytomatic	Z0334
**HSP70**	1/200		ms monoclonal	ThermoFisher Scientific	Clone 4G4, MA3-009
**LAMP1**	1/300		ms monoclonal	Abcam	ab25630
**LAMP2**	1/300		ms monoclonal	Abcam	ab25631
**MAP2**	1/500		ms monoclonal	Sigma	M1406
**MAP2**	1/500		chick polyclonal	BioLegend	822501
**MTCO2**	1/400		ms monoclonal	Abcam	ab3298
**phospho S129 α-Syn**	1/500		rb polyclonal	Abcam	ab59264
**phospho S129 α-Syn**	1/500	1/5000	rb monoclonal	Abcam	EP1536Y, ab51253
**phospho S129 α-Syn**		1/5000	ms monoclonal	Abcam	Clone 81A, ab184674
**PSD95**	1/500		ms monoclonal	Abcam	ab2723
**S100-β**	1/500		ms monoclonal	Abcam	ab 11178
**SQSTM1-p62**	1/400		ms monoclonal	Abcam	ab56416
**Synaptophysin**	1/1000		rb polyclonal	Millipore	AB16659
**TBR1**	1/500		rb polyclonal	abcam	ab31940
**α-Synuclein**	1/500		ms monoclonal	ThermoFisher Scientific	Clone syn211, MS-1572
**α-Synuclein**	1/500	1/5000	ms monoclonal	BD Bioscience	Clone 42, 610787
**Ubiquitin**	1/500		ms monoclonal	Abcam	ab7254
**VGLUT1**	1/1000		rb polyclonal	Synaptic System	135 303

SUPPLEMENTAL REFERENCES

Bousset, L., Pieri, L., Ruiz-Arlandis, G., Gath, J., Jensen, P.H., Habenstein, B., Madiona, K., Olieric, V., Bockmann, A., Meier, B.H., *et al.* (2013). Structural and functional characterization of two alpha-synuclein strains. Nat Commun *4*, 2575.

Deleglise, B., Lassus, B., Soubeyre, V., Alleaume-Butaux, A., Hjorth, J.J., Vignes, M., Schneider, B., Brugg, B., Viovy, J.L., and Peyrin, J.M. (2013). Synapto-protective drugs evaluation in reconstructed neuronal network. PLoS One *8*, e71103.

Macia, E., Ehrlich, M., Massol, R., Boucrot, E., Brunner, C., and Kirchhausen, T. (2006). Dynasore, a cell-permeable inhibitor of dynamin. Dev Cell *10*, 839-850.

Monsellier, E., Redeker, V., Ruiz-Arlandis, G., Bousset, L., and Melki, R. (2015). Molecular interaction between the chaperone Hsc70 and the N-terminal flank of huntingtin exon 1 modulates aggregation. J Biol Chem *290*, 2560-2576.

Nicoleau, C., Varela, C., Bonnefond, C., Maury, Y., Bugi, A., Aubry, L., Viegas, P., Bourgois-Rocha, F., Peschanski, M., and Perrier, A.L. (2013). Embryonic Stem Cells Neural Differentiation Qualifies the Role of Wnt/beta-Catenin Signals in Human Telencephalic Specification and Regionalization. Stem Cells *31*, 1763-1774.

Schlegel, R., Dickson, R.B., Willingham, M.C., and Pastan, I.H. (1982). Amantadine and dansylcadaverine inhibit vesicular stomatitis virus uptake and receptor-mediated endocytosis of alpha 2-macroglobulin. Proc Natl Acad Sci U S A *79*, 2291-2295.
Volpicelli-Daley, L.A., Luk, K.C., and Lee, V.M. (2016). Addition of exogenous alpha-synuclein preformed fibrils to primary neuronal cultures to seed recruitment of endogenous alpha-synuclein to Lewy body and Lewy neurite-like aggregates. Nat Protoc *9*, 2135-2146.
