# Supplementary material for: Propagation of α-Synuclein Strains within Human Reconstructed Neuronal Network
Source: Stem Cell Reports. 2019 Jan 10;12(2):230–44. doi: 10.1016/j.stemcr.2018.12.007 (PMC6372945; doi:10.1016/j.stemcr.2018.12.007)
Supplement: Document S2. Article plus Supplemental Information [file mmc4.pdf]

Stem Cell Reports
Article

OPEN ACCESS

Propagation of α-Synuclein Strains within Human Reconstructed Neuronal Network

Simona Gribaudo,[1,2,6] Philippe Tixador,[3,6] Luc Bousset,[4,6] Alexis Fenyi,[4] Patricia Lino,[1,2] Ronald Melki,[4,7,*] Jean-Michel Peyrin,[3,5,7,*] and Anselme L. Perrier[1,2,7,*]
[1]INSERM U861, I-STEM, AFM, Corbeil-Essonnes 91100, France
[2]UEVE U861, I-STEM, AFM, Corbeil-Essonnes 91100, France
[3]Sorbonne Universités, Faculté des Sciences et Ingénierie, CNRS/UMR 8256, B2A, Biological Adaptation and Ageing, Institut de Biologie Paris Seine, Paris 75005, France
[4]Laboratory of Neurodegenerative Disease, Institut François Jacob, MIRCen, CEA-CNRS, Fontenay aux Roses 92265, France
[5]Sorbonne Universités, Faculté des Sciences et Ingénierie, CNRS UMR 8246, INSERM U1130, Neurosciences Paris Seine, Institut de Biologie Paris Seine, Paris, 75005, France
[6]Co-first author
[7]Co-senior author
*Correspondence: ronald.melki@cnrs.fr (R.M.), jean-michel.peyrin@upmc.fr (J.-M.P.), anselme.perrier@inserm.fr (A.L.P.)
https://doi.org/10.1016/j.stemcr.2018.12.007

SUMMARY

Reappraisal of neuropathological studies suggests that pathological hallmarks of Alzheimer's disease and Parkinson's disease (PD) spread progressively along predictable neuronal pathways in the human brain through unknown mechanisms. Although there is much evidence supporting the prion-like propagation and amplification of α-synuclein (α-Syn) *in vitro* and in rodent models, whether this scenario occurs in the human brain remains to be substantiated. Here we reconstructed in microfluidic devices corticocortical neuronal networks using human induced pluripotent stem cells derived from a healthy donor. We provide unique experimental evidence that different strains of human α-Syn disseminate in "wild-type" human neuronal networks in a prion-like manner. We show that two distinct α-Syn strains we named fibrils and ribbons are transported, traffic between neurons, and trigger to different extents, in a dose- and structure-dependent manner, the progressive accumulation of PD-like pathological hallmarks. We further demonstrate that seeded aggregation of endogenous soluble α-Syn affects synaptic integrity and mitochondria morphology.

INTRODUCTION

Chronic neurodegenerative diseases are tightly linked to the progressive accumulation of abnormally folded protein conformers inside or outside neurons. These abnormal conformers are proposed to mediate neuronal dysfunctions. In synucleinopathies such as Parkinson's disease (PD), dementia with Lewy bodies (DLB), and multiple system atrophy (MSA), α-synuclein (α-Syn), a presynaptic protein involved in the regulation of synaptic vesicle release, accumulates in a phosphorylated and insoluble form in neurons. Aggregated α-Syn is the principal component of Lewy bodies and Lewy neurites, which are considered pathognomonic hallmarks of synucleinopathies (Goedert et al., 2013; Spillantini et al., 1997). As in Alzheimer's disease (Braak et al., 2006), PD/DLB/MSA pathological hallmarks progress spatially and temporally in the brains of affected patients (Braak et al., 2003b). This, together with the observation that Lewy bodies are detected in neurons grafted in PD patients (Kordower et al., 2008; Li et al., 2008), suggests that neuropathological hallmarks spread from diseased to naive neurons through unknown mechanisms.

Mounting studies show that α-Syn assemblies trigger the aggregation of soluble α-Syn both *in vitro* and *in vivo*, suggesting that aggregated α-Syn holds prion-like properties in rodents (Desplats et al., 2009; George et al., 2013; Guo et al., 2013; Hansen et al., 2011; Luk et al., 2012a; Peelaerts et al., 2015; Sacino et al., 2014a; Volpicelli-Daley et al., 2011). Indeed, intra-cerebral inoculation of brain homogenates from aged transgenic mice overexpressing mutant α-Syn to cognate young transgenic animals susceptible to spontaneous α-Syn aggregation or to wild-type (WT) mice leads to early motor deficits and α-Syn deposition in the rodent central nervous system (Betemps et al., 2014; Luk et al., 2012b; Masuda-Suzukake et al., 2013). Yet whether PD-associated disease hallmarks spread through a prion-like process in human brain remains to be substantiated. The extent to which fibrillar α-Syn assemblies bind to human mature neurons, are taken up, seed the aggregation of their soluble counterparts, trigger neuronal dysfunctions, and propagate from one neuron to another remains to be determined. Human pluripotent stem cell (hPSC) technologies have become a key asset for deciphering pathological mechanisms (Grskovic et al., 2011). Several proofs of concept have shown that hPSC differentiation into neurons is paramount to study neurodegenerative syndromes as it provides direct access to cultures enriched in human post-mitotic neurons with region/subtype-specific identities (Gaspard et al., 2009). Previously, we (1) demonstrated the reconstruction of fully functional and oriented rodent neuronal networks in microfluidic chips (Deleglise et al., 2014; Dinh et al., 2013;

This is an open access article under the CC BY license (http://creativecommons.org/licenses/by/4.0/).

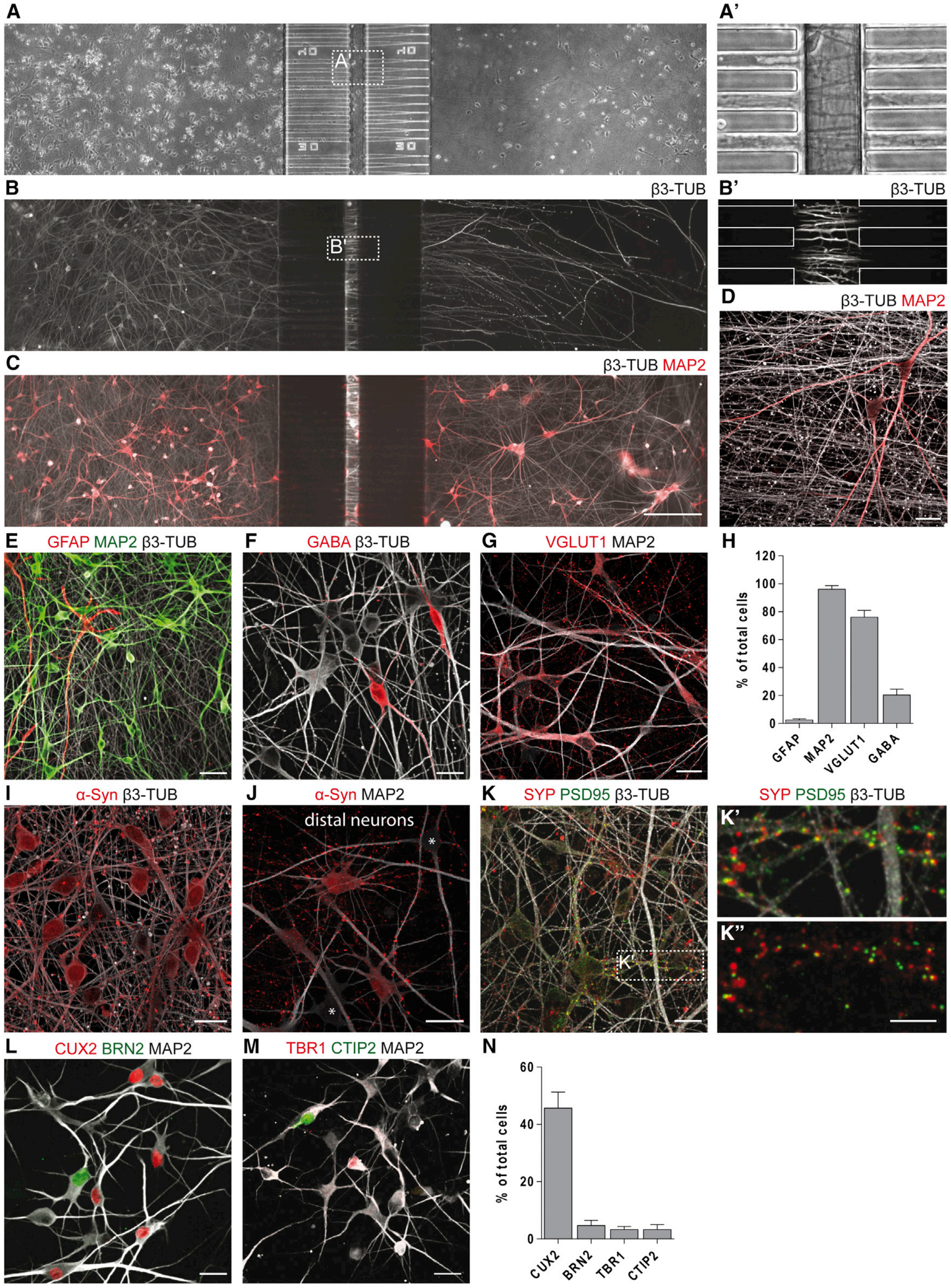

Figure 1. Characterization of hiPSC-Derived Corticocortical Neuronal Networks

(A) Phase-contrast stitched image of hiPSC-derived neuronal networks 30 days post-plating. (A′) Inset of central chamber invaded by axons.

(legend continued on next page)

Peyrin et al., 2011), (2) gained unlimited access to standardized human telencephalic neurons from hPSCs (Nicoleau et al., 2013), and (3) demonstrated uptake and active anterograde and retrograde transport of fibrillar α-Syn in mouse neurons (Brahic et al., 2016; Freundt et al., 2012). Here, taking advantage of these studies we reconstructed human induced pluripotent stem cell (hiPSC)-derived neuronal networks in microfluidic cell culture chambers and demonstrated that two structurally and functionally distinct α-Syn strains we named ribbons and fibrils (Bousset et al., 2013; Peelaerts et al., 2015) can efficiently propagate through such human WT neuronal networks. Both strains are able to reach the cytoplasmic compartment after direct internalization or upon neuron-to-neuron-mediated transfer (Abounit et al., 2016; Flavin et al., 2017) and elicit endogenous α-Syn aggregation in human cortical neurons. This process is progressive, and dose and structure dependent, with ribbons bearing a more potent seeding activity over a few weeks. Accumulation of α-Syn somatic inclusions correlates with neuronal activity impairment and mitochondrial dysfunctions. This model combining human connected WT neurons and a trigger input is representative of the sporadic form of synucleinopathies and indicates that α-Syn prion-like spreading occurs in human neuronal networks where α-Syn expression is physiological and may account for the progression of age-related neurodegeneration in humans. This model represents a significant improvement over previous models where WT or mutant α-Syn is overexpressed in rodents (Luk et al., 2012b; Peelaerts et al., 2015).

RESULTS

Reconstruction of Oriented Human Corticocortical Network

To assess α-Syn assembly propagative properties in a human neuronal network we sought to combine neurons generated from hiPSCs derived from a healthy donor with microfluidic technologies allowing the reconstruction of an oriented neuronal network. We generated dorsal telencephalic neuronal progenitors from hPSCs (iPSC lines i90cl17 and i90cl16) as previously described (Nicoleau et al., 2013) and differentiated them into post-mitotic neurons (Feyeux et al., 2012). Our microfluidic devices are composed of three independent cell culture chambers separated by asymmetric microchannels that allow unidirectional axonal growth from the emitting to the receiving chamber (Figures 1A and 1A′) (Deleglise et al., 2013; Peyrin et al., 2011). hiPSC-derived neurons extended neurites through the microchannel that surrounded neurons in the distal chambers within 4 weeks of plating (Figures 1A′–1D). Phenotype and cortical regional identity of human neurons were assessed by immunofluorescence after 30 days of differentiation in chips. These hiPSC-derived cultures were highly enriched in neurons (>90%, MAP2$^+$), with a small percentage differentiating into astrocytes (<3%, GFAP$^+$) (Figures 1E and 1H). The neurons were composed of about 20% inhibitory GABA$^+$ neurons (Figures 1F and 1H) and about 75% glutamatergic VGlut1$^+$ neurons (Figures 1G and 1H). We detected α-Syn expression in nuclei and perikarya of the majority of neurons (Figures 1I and 1J), with enrichment in synaptic boutons particularly visible in the distal chamber, where axons from proximal neurons converged on the resident cells (Figure 1J). Juxtaposition of pre-synaptic (synaptophysin, SYP) and post-synaptic density protein-95 (PSD95) staining along dendrites was detected as well, indicating the establishment of excitatory synapses (Figures 1K–1K″). Those neurons expressed a variety of cortical markers, including upper- (BRN2, CUX2; Figures 1L and 1N) and, to a lesser extent, deep-layer cortical markers (TBR1, CTIP2; Figures 1M and 1N). Altogether, our results indicate that mature oriented human corticocortical networks reminiscent of neuroanatomical pathways within human brain can be reliably obtained in the microfluidic devices we used.

(B–D) (B and C) Fluorescence images (white, β3-tubulin [β3-TUB]; red, MAP2) of hiPSC-derived neurons in the proximal chamber projecting their axons into the distal chamber (B) in the absence or (C) in the presence of target neurons. (B′ and D) Higher-magnification images showing (B′) axons crossing the central chamber of the microchannel compartment and (D) axon bundles exiting the microchannel in the distal chamber.

(E–H) Representative images and quantification of astrocytic (GFAP) and neuronal (MAP2, β3-TUB, GABA, VGLUT1) marker expression. (E) GFAP, MAP2, β3-TUB; (F) GABA, β3-TUB; (G) VGLUT1, MAP2; (H) percentage of cells expressing each marker.

(I and J) (I) Expression of α-Syn in nuclei and perikarya of neurons (asterisks point out negative cells) and enrichment in synaptic boutons (J, distal chamber).

(K–K″) (K) Identification of putative excitatory synapses by juxtaposition of pre- (red, SYP) and post- (green, PSD95) protein complexes along neurites (white, β3-TUB). (K′) Magnification of box indicated in (K).

(L–N) Representative images and quantification of a repertoire of distinct cortical neuronal markers. (L) CUX2, BRN2, MAP2; (M) TBR1, CTIP2, MAP2; (N) percentage of cells expressing each marker.

Data are shown as mean ± SEM; n = 3. Scale bars: 200 μm in (A–C), 20 μm in (A′, B′, and D), 40 μm in (E), 20 μm in (F, G, and I–M), 10 μm in (K′ and K″).

Axonal Transport and Inter-neuronal Spreading of α-Syn Assemblies in Reconstructed Human Corticocortical Networks

We used our setup to investigate in a human context the properties of two distinct α-Syn strains, coined as fibrils and ribbons (Bousset et al., 2013), that have been previously shown to trigger distinct synucleinopathies in rodents (Peelaerts et al., 2015). To assess their prion-like behavior in hiPSC-derived cortical neurons, we specifically measured the uptake, intra-cellular transport, inter-neuronal transfer, and seeding propensity of each strain.

To determine whether α-Syn assemblies could be taken up and transported along the axons, neurons within the proximal chamber of 30 day old reconstructed human corticocortical networks were exposed to Atto-550-labeled α-Syn monomers, fibrils, or ribbons. The fluorescent signal was longitudinally analyzed by live imaging microscopy in both proximal and distal microfluidic chambers 1, 7, 15, and 21 days post-exposure (dpe). Starting from 1 dpe about 90% of the neurons in the proximal chamber exposed to both ribbons and fibrils clearly presented Atto-550 dots in their cytoplasm that persisted over time. In contrast, cells exposed to monomeric α-Syn exhibited a diffuse Atto-550 signal with very few puncta that were almost completely lost over time (Figure 2A, left), suggesting that monomeric and fibrillar α-Syn have different clearance dynamics. To gain molecular insight into fibrillar α-Syn uptake, we exposed neurons to monocadaverin, an inhibitor of receptor-mediated endocytosis, or to dynasore, an inhibitor of dynamin, prior to addition of the exogenous α-Syn fibrillar strains. Uptake of Atto-550 α-Syn strains (fibrils and ribbons) was strongly reduced by monocadaverin (70 μM), while dynasore (80 μM) only partially reduced uptake (Figure S2A). Ordered movements of small fluorescent exogenous α-Syn puncta were evidenced in the axonal extension crossing the microchannel barrier. We distinguished between anterograde and retrograde axonal transport of fluorescent α-Syn strains, performing time-lapse imaging at the exit of microchannels in the distal chamber. Both strains were transported anterogradely and retrogradely in human neurons (Figures 2B and 2C and Videos S1 and S2). The mean anterograde velocities were 2.6 ± 1.4 and 2.7 ± 0.9 μm/s for fibrils and ribbons, respectively, consistent with the fast component of axonal anterograde transport. Assemblies transported retrogradely moved at 1.3 ± 0.8 μm/s for the fibrils and 1.2 ± 0.7 μm/s for ribbons (Figure 2C), thus creating a net axonal anterograde flux upon somatodendritic loading. During the time of recording, the two α-Syn strains displayed similar average run lengths (98–128 μm) (Figure 2D).

We next quantified the spread of α-Syn strains from neurons in the proximal chamber to connected neurons in the distal chamber (Figure 2A, right). Again, the proportion of neurons with detectable signal from Atto-550-labeled monomeric α-Syn was very low and almost disappeared over time, whereas that of neurons presenting Atto-550 dots in their soma after exposing afferent neurons in the proximal chamber to fibrils or ribbons significantly increased up to 15 dpe, and then plateaued (Figure 2A, right). Similar inter-neuronal transfer capacities were observed for the two α-Syn strains. Monocadaverin and dynasore affected inter-neuronal transfer (from the proximal to the distal chambers of the microfluidic device) of Atto-550 α-Syn strains (Figures S2B and S2C). Thus, endocytosis appears to contribute significantly to α-Syn assembly spreading between human cortical neurons. Overall, these data demonstrate that α-Syn strain spread between human neurons is a fast and efficient process involving internalization followed by anterograde axonal transport to the axonal ends and spread to secondary neurons. Interestingly, the efficiency of uptake, transport, and inter-neuronal transfer over time was similar for fibril and ribbon strains (Figure 2A).

Seeding of Endogenous α-Syn in Human Cortical Wild-Type Neurons

Seeding and accumulation of phosphorylated host-encoded cognate α-Syn following trans-neuronal propagation is key for the concept of prion-like spreading along nerve pathways in PD. We therefore assessed whether exogenous human α-Syn strains provide a template for the assembly of endogenous WT human α-Syn into aggregates characteristic of pathological α-Syn. Mature (30 day old) hiPSC-derived cortical neurons were exposed to monomeric α-Syn, ribbons, or fibrils for 24 h and incubated for 30 days. Additional control conditions included human cortical neurons exposed to Atto-550-labeled fibrillar human huntingtin exon 1 with 48 glutamine residues (HTTExon1Q48). We used both immunofluorescence and biochemical analysis to assess the formation of abnormal α-Syn, monitoring in particular its detergent insolubility and its phosphorylation at position serine 129 (S129).

Thirty days after exposure of hiPSC-derived cortical neurons to α-Syn fibrils and ribbons, phosphorylated α-Syn (α-SynP) clumps were detected (Figures 3A and 3B). No α-SynP signal was detected upon neuronal exposure to either monomeric α-Syn or HTTExon1Q48 fibrillar assemblies (Figures 3C and 3D). These results were confirmed using a second α-SynP antibody (α-SynP EP1536Y), which was previously demonstrated not to cross-react with phosphorylated neurofilament (Rutherford et al., 2016; Sacino et al., 2014b) (Figure 3E).

We then challenged human cortical neurons with a version of α-Syn strain that retains its deleterious activity while being non-phosphorylatable at amino acid residue S129 because this residue was changed into an alanine

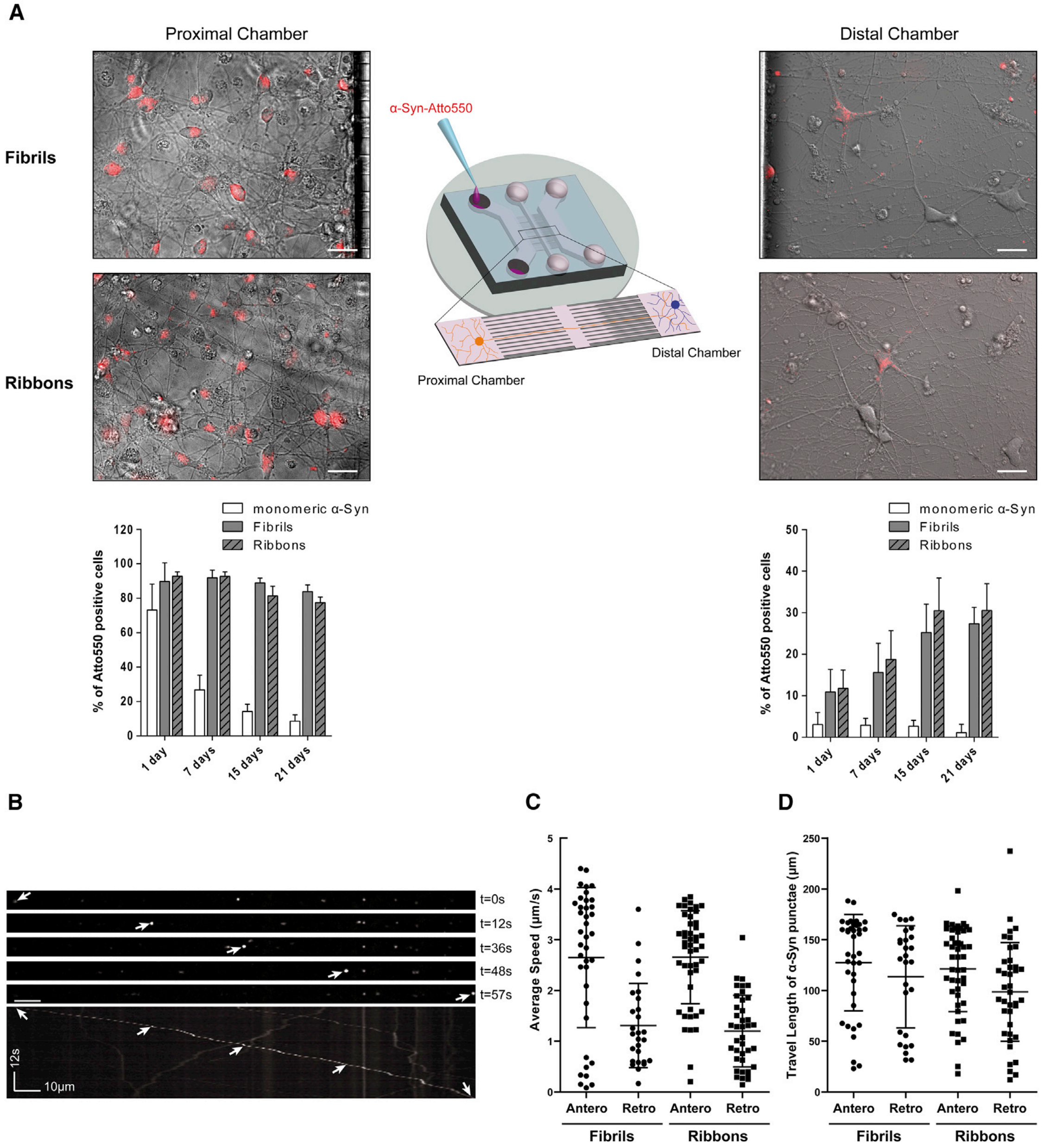

Figure 2. Intra-neuronal and Inter-neuronal Spreading of α-Syn Assemblies

(A) Fluorescence and phase-contrast images of 30 day old corticocortical neuronal networks exposed for 24 h in the proximal chamber (left) to 500 nM Atto-550-labeled fibrils (top) or ribbons (bottom). The bar graphs show quantitative assessment of Atto-550-labeled α-Syn monomers, fibrils, and ribbons taken up by loaded neurons (left) and transferred to receiving neurons (right) at 1, 7, 15, and 21 dpe. Significant differences were observed between strains and between days post-exposure in both proximal and distal chambers (for statistical analysis see Supplemental Information). Data are shown as ± SD.

(B) Kymograph analysis of α-Syn fibril movement along axons extending from the proximal chamber in a typical recording performed at 24 h post-loading.

(C and D) Analysis of α-Syn assemblies' anterograde and retrograde movement in axonal tracts. (C) Average speed. (D) Travel length. Data are shown as mean ± SD. Scale bars: 25 μm.

(Febbraro et al., 2013; Gorbatyuk et al., 2008). After exposure to fibrillar S129A α-Syn exogenous assemblies, human mature neurons exhibited aberrant phosphorylation and clustering of the endogenous α-Syn (Figure 3F, and data not shown for α-SynP EP1536Y antibody). Altogether, these results show that exogenous α-Syn ribbons and fibrils

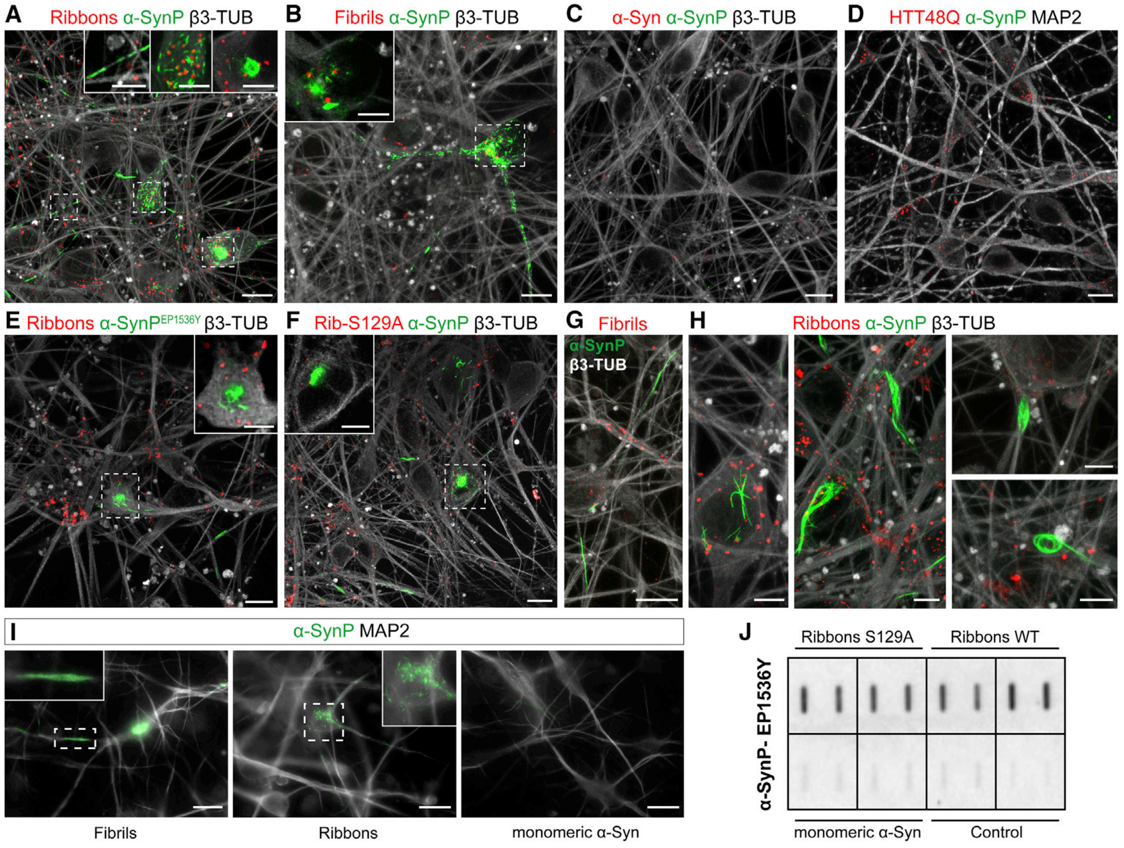

Figure 3. Immunofluorescence Analysis of Seeded Endogenous α-Syn Aggregation in hiPSC-Derived Cortical Neurons

(A–F) Fluorescence confocal imaging of endogenous α-SynP (green) in human cortical neurons (β3-TUB or MAP2, gray) at 30 dpe to 500 nM Atto-550 (red) (A and E) α-Syn ribbons, (B) α-Syn fibrils, (C) monomeric α-Syn, (D) HTTExon1Q48, and (F) S129A ribbons using two different α-SynP antibodies (α-SynP, ab59264 in A–D and F–H, and α-SynP^{EP1536Y}, ab51253 in E). The regions boxed with dashed lines are enlarged and represented as single plane in the insets. Scale bars: 10 μm, 5 μm in insets.

(G and H) Representative images showing that α-SynP inclusions triggered by either (G) fibrils or (H) ribbons exhibit different classes of shapes (green, α-SynP; white, β3-TUB; red, exogenous assemblies); scale bar: 10 μm in (G), 5 μm in (H).

(I) Resistance of endogenous α-SynP signal to paraformaldehyde/Triton X-100 fixation/extraction immunofluorescence protocol (green, α-SynP; white, MAP2); scale bar: 20 μm.

(J) Filter trap assay for α-SynP (α-SynP^{EP1536Y} antibody) in cortical neuron lysates at 30 dpe to ribbons made of wild-type or S129A α-Syn or monomeric α-Syn or in the absence of treatment. Each square represents duplicates of the sample.

trigger the specific formation of inclusions made of endogenous α-SynP in hiPSC-derived cortical neurons. Astroglial cells in our cultures readily took up the exogenous assemblies that strongly associate with lysosomal marker (Figure S3A) but, in contrast to neurons, none of those cells ever contained α-SynP structures (Figure S3B), thus evidencing that human astrocytes do not contribute actively to the amplification of α-Syn strains. Both strains promoted the formation of α-SynP inclusions in neurons bearing different aspects, including compact agglomerated, annular, and filiform/serpentine structures in both the somatodendritic compartment and the axonal endings (Figures 3A, 3B, and 3E–3H). Interestingly, α-SynP inclusions present in the somatic compartment were always found juxtaposed to exogenous Atto-550-labeled α-Syn or α-Syn S129A ribbons or fibrils (single-plane insets in Figures 3A, 3B, 3E, and 3F), suggesting a physical interaction between the exogenous input and the α-SynP somatic inclusions. The α-SynP inclusions formed upon fibril and ribbon exposure are resistant to a paraformaldehyde/Triton X-100 fixation/extraction and immunofluorescence protocol (Figure 3I), indicating that α-SynP morphotypes have an insoluble nature (Volpicelli-Daley et al., 2016).

These results were confirmed using a filter trap assay (Figure 3J), in which comparable amounts of α-SynP signal were reproducibly detected in samples originating from neurons exposed to WT or mutant S129A α-Syn ribbons. This further proves that the α-SynP we detect corresponds

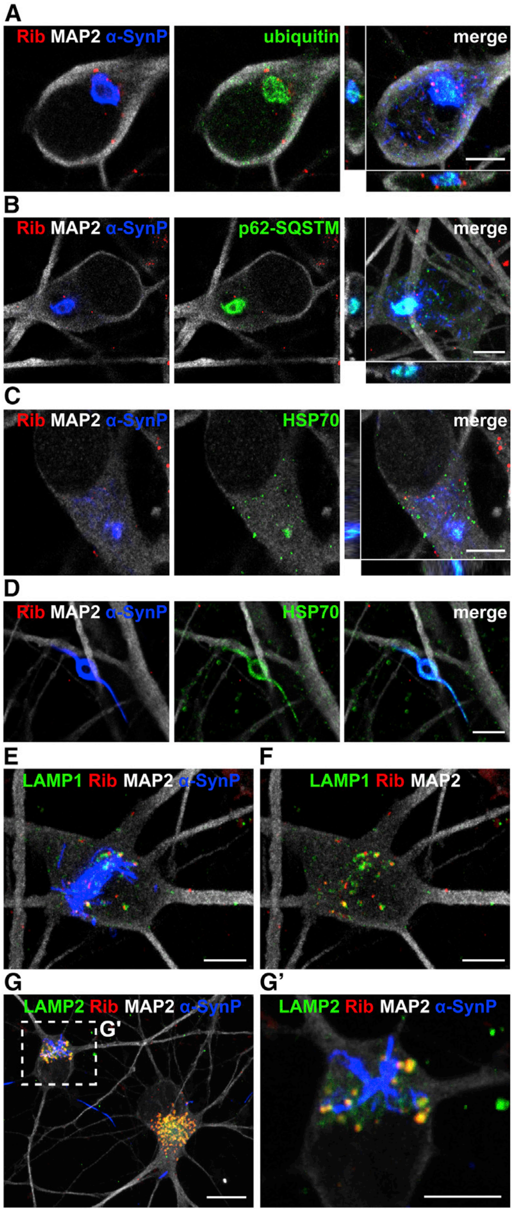

Figure 4. Characterization of α-SynP Somatodendritic Inclusions
(A–D) (A) Anti-ubiquitin, (B) anti-p62-sequestosome1, and (C and D) anti-HSP-70 immunostaining (green) and colocalization with endogenous α-SynP aggregates (blue) in neurons (MAP2, white) treated with ribbons (abbreviated here Rib, red) at 21 dpe. HSP-70 labeled the dense core of (C) somatic or (D) neuritic α-SynP inclusions. For each line the first two images represent single plane, whereas merge panels are composed of z stack projection.
(E–G) (E and F) LAMP1 and (G and G′) LAMP2 immunostaining (green) largely colocalized with exogenous assemblies (ribbons, red) but did not show appreciable association with α-SynP structures (blue).
Scale bars: 5 μm in (A–F and G′); 10 μm in (G).

to seeded endogenous cortical neurons' α-Syn, not the exogenous seeds, as they are not recognized by the α-SynP S129 antibodies. We did not observe α-SynP in neurons exposed to monomeric α-Syn.

We next labeled oligodendrocytes and oligodendrocyte progenitors with the O4 antibodies in our human cortical cultures exposed to α-Syn assemblies (21 dpe). We observed that exogenous α-Syn assemblies are readily taken up by the few $O4^{+}$ cells present in our cultures (Figure S4). In line with our *in vivo* observation in Peelaerts et al. (2015), no α-SynP inclusions could be detected in those $O4^{+}$ cells (Luk et al., 2012b; Peelaerts et al., 2015).

Characterization of α-SynP Inclusions in Human Wild-Type Neurons

The cores of the large somatodendritic α-SynP inclusions were positive for ubiquitin (Figure 4A) and strongly colocalized with SQSTM1-p62 (Figure 4B), an adaptor protein that delivers ubiquitinated cargoes to autophagy. In addition, the dense core of somatic inclusion as well as the neuritic filamentous inclusions was partially labeled by HSP-70 (Figures 4C and 4D), further indicating that endogenous aggregated α-Syn is targeted to degradation. We next assessed the association of exogenous assemblies and endogenous α-SynP inclusions with the lysosomal compartment in neurons. Exogenous α-Syn fibrils and ribbons largely colocalized (80% colocalization) with the lysosomal markers LAMP1 and LAMP2 at 21 dpe, indicating that the assemblies persist within the lysosomal compartment over several weeks. In contrast, α-SynP aggregates, including large filamentous, annular, or fragmented structures, were not found associated with LAMP1 and LAMP2 staining, which suggests that they are not within lysosomes/autophagosomes but within the cytosol. These structures resist degradation and accumulate in the cytoplasm of neurons. Altogether the variety of intra-neuronal inclusions we observed following exposure of human neurons to α-Syn fibrils and ribbons strikingly resembles, based on morphology, distribution, and colocalization with components of the protein degradation machinery, Lewy bodies and Lewy neurites found in the brain of PD patients (Goedert et al., 2013).

Differential Seeding Properties of Ribbons and Fibrils: Dose- and Time-Dependence Analyses in Human Cortical Neurons

Using the ratio between the area occupied by α-SynP and MAP2 in neurons exposed to α-Syn strains as a readout of strain nucleation capacities, we performed dose-dependence analyses treating cells with up to 4.5 μM ribbons or fibrils. The data we obtained with ribbons at 15 dpe fitted a sigmoidal curve (Figures 5A and 5B) and allowed us to estimate a half-maximal effective concentration

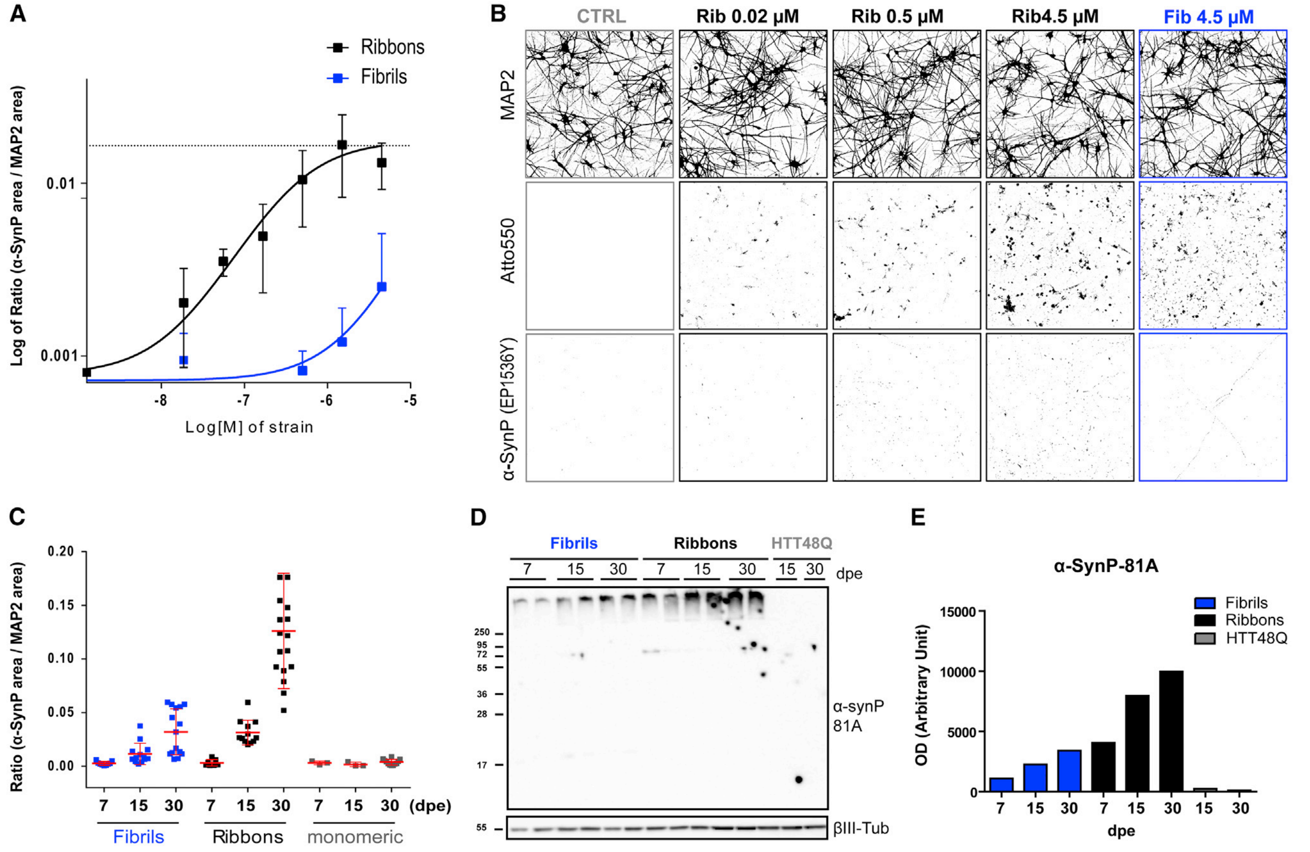

Figure 5. Dose- and Time-Dependent Effect of α-Syn Strain Exposure

(A) Ratio (logarithmic scale) between the area occupied by α-SynP and MAP2 at increasing concentration of fibrils and ribbons ($0.02 < x < 4.5$ μM, 15 dpe; mean ± SD; n = 2).

(B) Binary threshold masks of MAP2, Atto-550 assemblies, and α-SynP structures of control neurons (untreated, gray boxed) or neurons exposed at different concentrations of ribbons (black boxed) or fibrils (blue boxed).

(C) Ratio between the area occupied by α-SynP and MAP2 over time (7, 15, and 30 dpe; [strains] = 500 nM). Data are shown as mean ± SD, n = 3 (for statistical analysis see Supplemental Information).

(D and E) (D) Western blot analysis and (E) quantification showing the progressive accumulation of α-SynP in the insoluble fractions of neuron lysates at 7, 15, and 30 dpe to fibrils, ribbons, or fibrillar HTTExon1Q48 (α-SynP-81A antibody).

(EC_{50}) (355 nM). For fibrils, the data fitted only partially a sigmoid curve and indicated an EC_{50} likely 2 log larger than that of ribbons. Interestingly, no significant changes in cortical neuron viability (pyknotic/normal DAPI-positive nuclei) or overall morphology (MAP2 total area per cell) were observed at any concentration of ribbons or fibrils we used (Figure 5B).

Next, we assessed the progressive accumulation of α-SynP in human cortical neurons exposed for 24 h to exogenous α-Syn fibrils and ribbons (0.5 μM) from 7 to 30 dpe (Figure 5C). The α-SynP/MAP2 ratio significantly increased with time, while it remained constant in neurons exposed to monomeric α-Syn. A higher α-SynP signal was measured upon exposure of neurons to ribbons compared with identical amounts of fibrils, in particular at the longest time point we analyzed (30 dpe, $p < 0.001$). These results were confirmed by biochemical analysis, where the amount of sarkosyl-insoluble α-SynP increased from 7 to 30 dpe for both strains (Figure 5D) and was 3-fold higher in cells exposed to ribbons compared with fibrils (Figure 5E). Altogether these experiments show that the exposure of WT human cortical neurons to α-Syn strains triggers the progressive conversion of homotypic neuronal α-Syn into insoluble and α-SynP aggregates and that this phenomenon depends on the strain conformation, with ribbons being more efficient than fibrils.

Neuronal Dysfunctions Associated with Exposure to α-Syn Strains and α-SynP Accumulation

We next investigated whether human neurons treated with α-Syn assemblies featured any type of early signs of neuronal dysfunction weeks after exposure to the strains

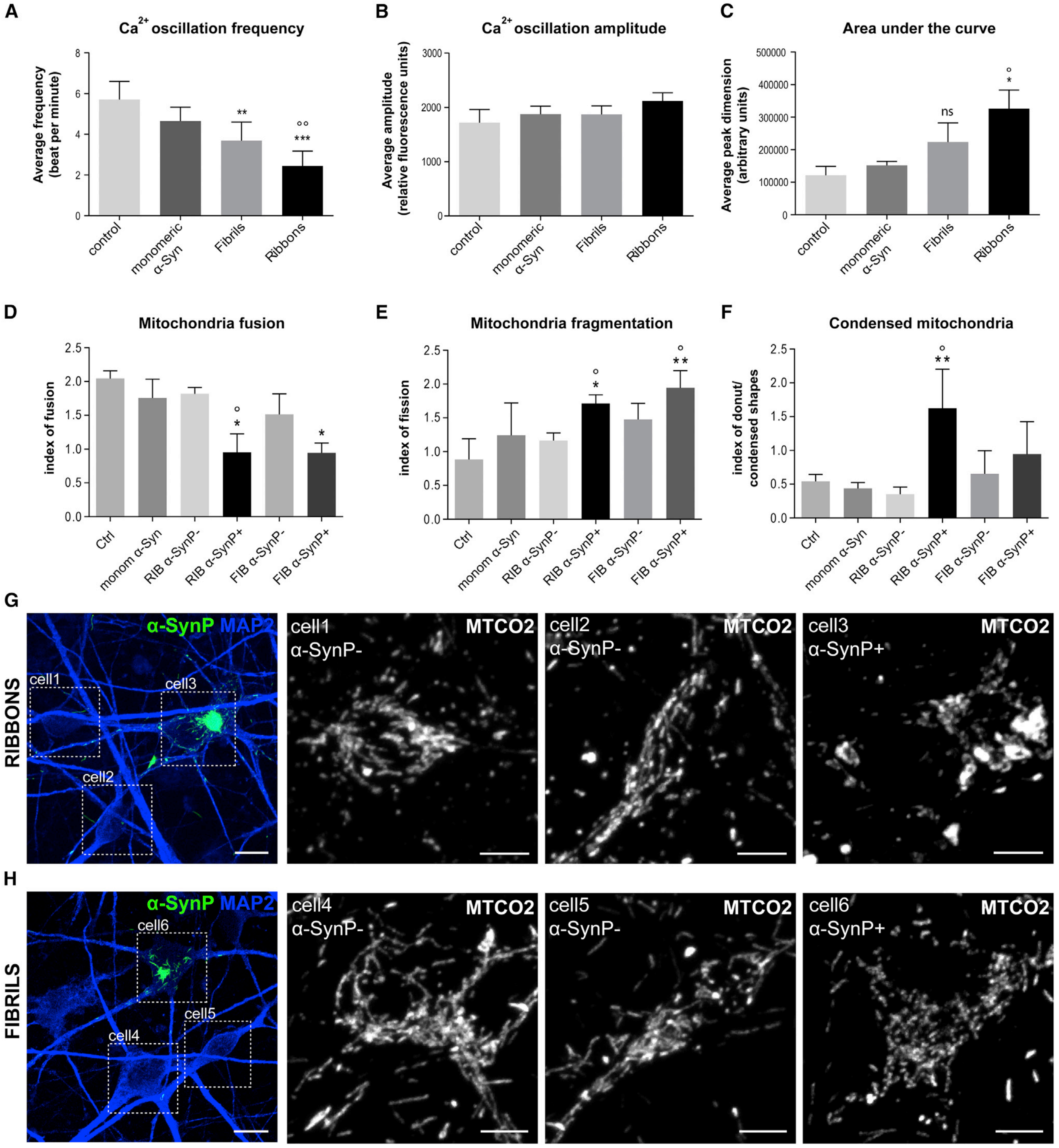

Figure 6. Alteration in Ca^{2+} Homeostasis and Morphological Impairment of Mitochondrial Networks in Human Neurons Following Exposure to α-Syn Strains and α-SynP Accumulation

(A–F) (A–C) Whole-well Ca^{2+} imaging analyses on mature human cortical neurons 19 dpe to fibrils, ribbons, monomeric α-Syn, and untreated neurons. The (A) average frequency, (B) average amplitude, and (C) average area under the curve of the Ca^{2+} waves were analyzed for each well. Asterisks indicate significant difference of α-Syn strain-treated neurons versus control; circles indicate significant difference of α-Syn strain-treated neurons versus monomeric α-Syn. (D–F) Analysis of the index of (D) fusion, (E) fragmentation, and (F) donut-shaped/condensed mitochondria in untreated neurons or neurons at 21 dpe to monomeric α-Syn, ribbons, or fibrils. Asterisks indicate

(legend continued on next page)

despite the absence of evident morphological demise. We first performed Ca^{2+} imaging analysis on mature human cortical neurons exposed to ribbons, fibrils, and monomeric α-Syn. Synchronized spontaneous Ca^{2+} oscillations were recorded at 1 and 19 dpe using whole-well Fluo-4 calcium imaging (Figures 6A–6C and S5). All the parameters of these oscillations were comparable for untreated and monomeric α-Syn-treated neuronal cultures at 1 and 19 dpe. We detected minor alterations of average frequency and dimension (area under the curve, AUC) of Ca^{2+} waves at 1 dpe in α-Syn strain-treated neuronal cultures (Figures S5B and S5C). The extent of these alterations increases with time. At 19 dpe the frequency (Figures 6A and S5A) and the AUC (Figures 6C and S5A) were significantly altered for cultures exposed to ribbons (compared with untreated or monomeric α-Syn-treated cultures at 19 dpe), while the amplitude remained unchanged (Figure 6B); 19 dpe to fibrils triggered only a significant decrease in frequency (Figures 6A and S5A). The α-SynP/MAP2 ratio measured by immunofluorescence on cultures at 19 dpe and after Ca^{2+} analysis (same well analysis) showed nucleation levels similar to those presented in Figure 5C. Our observations indicate that neuronal cultures exposed to α-Syn strains present alterations in Ca^{2+} homeostasis that worsen over time in parallel with the progression in α-SynP accumulation (Figure S5B).

Several lines of evidence link mitochondrial dysfunction and α-Syn aggregation (Di Maio et al., 2016; Grassi et al., 2018; Prots et al., 2018; Reeve et al., 2015). We thus compared the morphology of mitochondria networks in control neurons and neurons exposed to ribbons, fibrils, and monomeric α-Syn (21 dpe). Neurons bearing somatic α-SynP inclusions induced by exposure to either ribbons or fibrils presented altered mitochondria morphologies. In particular, we observed significant reduction of tubular fused networks (Figures 6D, 6G, and 6H), increase in the proportion of fragmented mitochondria (Figures 6E, 6G, and 6H), and increase in the donut-shaped/condensed mitochondria (Figures 6F–6H). Control cells (unexposed or exposed to monomeric α-Syn) or cells lacking α-SynP inclusions from cultures exposed to exogenous α-Syn strains did not exhibit such alterations (Figures 6D–6F). These results demonstrate co-occurrence in human neurons of seeding events of endogenous α-Syn triggered by ribbons or fibrils and mitochondrial impairment.

Phosphorylated α-Syn Propagation through Fluidically Isolated Human Cortical Neuronal Networks

Last we investigated whether exogenous α-Syn assemblies loaded only in the proximal chamber triggered, after spreading between neurons, the seeding of endogenous α-Syn in second-order neurons (i.e., located in the distal chamber). For this, neurons grown in the proximal chamber of corticocortical networks were challenged with α-Syn assemblies, either WT or S129A α-Syn ribbons, and the time course of α-SynP inclusions was monitored in the distal chamber. Starting from 15 dpe, α-SynP structures, mainly filiform shaped, were clearly detected in axons expanding from neurons residing in the emitting chamber (Figure 7A) but not in the unchallenged resident neurons. Three weeks after the exposure of proximal cells to ribbons made of either WT or S129A-α-Syn, α-SynP-positive clusters could be detected in the cytoplasm of a few second-order neurons in the distal chamber. The α-SynP structures ranged from small dots to more complex filiform architectures (Figures 7B and 7C), mainly detectable in the somatic compartment of second-order neurons of the distal chamber. Almost all the cells presenting α-SynP signal in the distal chamber also contained detectable exogenous aggregates, with very rare exception, thus suggesting that trans-neuronal nucleation was mainly due to initial seeds taken up by neurons in the proximal chamber that were subsequently transferred to second-order acceptor neurons.

DISCUSSION

Prion-like propagation of α-Syn aggregates (Brundin and Melki, 2017; Melki, 2015) is a scenario that could identify the sources of exogenous, non-genetic initiation of PD in patients and account for the sequential appearance of pathological hallmarks in different brain areas (Braak et al., 2003a; Jellinger, 2012). Here, we provide results that validate the prion-like propagation hypothesis of α-Syn aggregates at the human level. This is done in human neuronal cultures, made of cortical neurons and glial cells (>90% and <3% of cells, respectively), expressing genome-encoded WT α-Syn, as opposed to models where transgenic WT or mutant α-Syn is overexpressed (Luk et al., 2012b; Peelaerts et al., 2015).

significant differences of treated neurons versus control; circles indicate significant differences of α-SynP$^+$ versus α-SynP$^-$ cells in the same culture. * and $^\circ p < 0.05$, ** and $^{\circ\circ} p < 0.01$, ***$p < 0.001$. Data are shown as mean ± SEM, n = 3 (for statistical analysis see Supplemental Information).

(G and H) Representative confocal images of neurons exposed to (G) ribbons and (H) fibrils. On the left the cells are stained for MAP2 (blue) and α-SynP (green). Highlighted cells (dotted lines) are enlarged and stained with MTCO2 (white). Cells 1, 2, 4, and 5 are α-SynP$^-$. Cell 3 and cell 6 are α-SynP$^+$. Scale bars: 10 μm in left image of (G) and (H); 5 μm in cell insets.

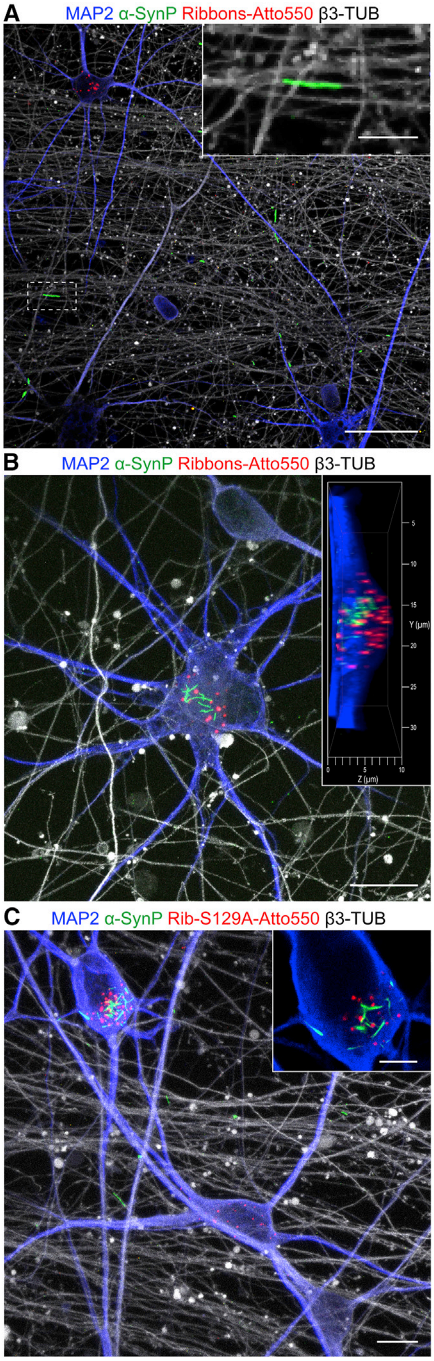

Figure 7. Induction of Trans-neuronal Nucleation

Fluorescence confocal imaging illustrating endogenous α-SynP (green) in the distal chamber upon seeding of proximal neurons with exogenous α-Syn ribbons (made of WT in A and B, S129A α-Syn in C, red). Axons deriving from proximal neurons are identified using β3-tubulin (β3-TUB, gray), while neurons residing in the distal chamber are identified by MAP2 staining (blue).

(A) α-SynP filiform structures are present in proximal neurons axons at 15 dpe.

(B and C) At 21 dpe, α-SynP-positive clusters are detected as well in the cytoplasm of sparse distal neurons. Insets in (A) and (C) are enlargements of specific regions (highlighted with dotted lines if needed). Inset in (B) represents 3D reconstruction and orthogonal view of the intra-cellular α-SynP structures (Airyscan super resolution). Inset in (C) represents a single plane of the α-SynP cell. Scale bars: 40 μm in (A), 10 μm in (B) and inset in (A), 20 μm in (C), 5 μm in inset in (C).

We demonstrate that two different strains of human α-Syn (fibrils and ribbons) are transported, traffic between human neurons, and trigger the progressive accumulation of PD-like pathological hallmarks by recruiting the endogenous soluble α-Syn. We show that these assemblies act in a dose- and structure-dependent manner. The PD hallmarks they trigger in WT human cortical neurons, which include Lewy body- and Lewy neurite-like structures consisting of insoluble deposits of α-SynP, SQSTM1-p62, and HSP-70, correlate with early signs of neuronal dysfunctions such as alterations in calcium homeostasis and mitochondrial morphology.

We aimed at modeling α-Syn prion-like propagation in human corticocortical neuronal networks because it is affected at different stages of sporadic PD, DLB, and MSA (Braak et al., 2003a; Salvesen et al., 2017; Sugiura et al., 1995; Yau et al., 2018) and the conversion of endogenous WT α-Syn into pathological α-SynP aggregates may contribute to neuronal dysfunction in excitatory glutamatergic cortical neurons. Moreover, we chose to use hiPSCs from normal subjects specifically to model pathological processes involving α-Syn protein in idiopathic PD patients, by far the most numerous PD patients.

Our observation that α-SynP aggregates accumulate progressively over 30 days in neuronal axons and somatodendritic compartments suggests that α-Syn strains trigger sustained nucleation of host-encoded neuronal α-Syn. The fact that fluorescently labeled exogenous α-Syn strains do not overlap with α-SynP foci also shows that exogenous α-Syn assemblies not only are not phosphorylated, in agreement with previous findings (Pieri et al., 2016), but trigger endogenous α-Syn aggregation, with subsequent massive phosphorylation of the latter. To further strengthen this set of converging evidence, we used not only different anti-α-SynP antibodies (Rutherford et al., 2016; Sacino et al., 2016; Uchihara and Giasson, 2016) but also strains made of variant α-SynP (α-Syn S129A) that cannot be phosphorylated on residue S129, and thus are undetectable with anti-phospho-S129 α-Syn antibodies. Our data unambiguously show that (1) exogenous α-Syn strains trigger the progressive accumulation of PD-related hallmarks in human WT neurons by progressive corruption of the endogenous α-Syn and (2) this phenomenon is not prevented by mutations that compromise

phosphorylation of exogenous seeds on this specific site (Azeredo da Silveira et al., 2009; Tenreiro et al., 2014). Furthermore, we showed that large cytosolic α-SynP somatic inclusions bear marks of cellular recognition as aggregated proteins and are targeted to autophagy as demonstrated by colocalization with ubiquitin, SQSTM1-p62, and HSP-70. Interestingly, this was observed only in neurons under our experimental conditions. Indeed, while rare astrocytes present in the cell culture chambers displayed internalized fluorescent α-Syn assemblies, we were never able to detect α-SynP inclusion in these cells. This is due to either lower seeding efficiency of exogenous α-Syn strains or higher clearance efficiency of aggregated α-Syn in astrocytes (Loria et al., 2017). Exogenous assemblies were rapidly internalized by most of the directly exposed neurons within the proximal chamber and transported along their axons to cells residing in the distal chamber. We confirmed that the direct uptake of extracellular aggregates from the medium as well as the inter-neuronal transfer from acceptor to donor neurons mainly relies on endocytic processes (Apetri et al., 2016; Flavin et al., 2017; Lee et al., 2008; Sacino et al., 2016) (Brahic et al., 2016; Freundt et al., 2012).

Both α-Syn strain assemblies were transported with similar axonal velocities. They also propagated between neurons at similar rates. This is interesting and may suggest that α-Syn strains are transported by a given vehicle/machinery as opposed to other pathogenic protein assemblies that are transported and exported at different rates (Brahic et al., 2016). We report that both α-Syn strains promoted the formation of α-SynP structures resembling Lewy bodies and Lewy neurites. Nonetheless, ribbons were consistently more potent in inducing the accumulation of pathological α-SynP in human WT neurons. This is in agreement with observations we and others made in mouse (Peelaerts et al., 2015; Peng et al., 2018) and likely reflects a higher seeding propensity. Phenotypically distinct PrP-derived prion strains (scrapie, Creutzfeldt-Jakob disease) are discriminated using several parameters, among which is the seeding propensity. While slow versus fast propagation phenotypic traits are associated with cellular PrP expression level, they are also linked to differences in biochemical properties of PrP aggregates or in their cell targeting properties (Le Dur et al., 2017). In that regard, we consider that ribbons and fibrils do harbor a distinct strain phenotype that is reminiscent of fast versus slow propagation properties.

In the absence of major morphological alteration or neuronal loss upon exposure to exogenous α-Syn strains we assessed early signs of neuronal dysfunctions. We observed alterations in Ca^{2+} homeostasis in human neuronal networks exposed to α-Syn strains reminiscent of synaptic failure. These alterations paralleled the progression in α-SynP accumulation. These results are coherent with recent observations reporting functional defect in mouse hippocampal (Froula et al., 2018) and pyramidal (Kaufmann et al., 2016) neurons exposed to the fibrillar form of α-Syn. Moreover, focusing on neurons with large somatic inclusions, we observed a strong correlation between α-Syn seeding and mitochondria morphological impairment. A relationship between mitochondrial dysfunction and α-Syn aggregation was recently and independently evidenced (Di Maio et al., 2016; Grassi et al., 2018; Prots et al., 2018; Reeve et al., 2015). Our findings, in a relevant human cellular model, further highlight a role for α-Syn seeded aggregation in the development of functional defects likely initiating neurotoxicity associated with PD.

Finally, taking advantage of our microfluidic setup, we were able to evidence seeding of endogenous α-Syn in naive acceptor neurons after transfer of assemblies from donor neurons exposed to exogenous α-Syn seeds. This shows that prion-like propagation in neuronal networks comprises at minimum two distinct components, trans-neuronal spreading and intra-cellular seeding, the latter being the rate-limiting factor for amplification. Most importantly our results indicate that pathological forms of α-Syn actively disseminate and amplify in WT human neuronal networks by recruiting soluble α-Syn, a key step in the transmission of α-Syn pathogenic assemblies between human neurons in PD and related synucleinopathies.

The "Human Brain-on-Chips" functional platform we set up is uniquely suited to further assessing the prion-like spreading of aggregated α-Syn and its functional impact on neurons in distinct synucleinopathies. Because it is based on human neurons, expressing physiological levels of WT α-Syn, and preserves some degree of anatomical relevance, our *in vitro* model of a human neuronal network greatly extends the throughput at which many, if not all, aspects of prion-like spreading can be studied. A key advantage of our model is, in addition, that it bypasses the inter-species intrinsic differences in age-related neurodegenerative phenomena between rodents and humans. We are confident that such system will prove important as a predictive preclinical model to decipher the different components of the prion-like propagation (internalization, transport, nucleation) of α-Syn and of other protein aggregates in distinct neuropathological disorders and most importantly to later assess the efficiency of disease-modifying therapeutic approaches targeting key components of such pathways.

EXPERIMENTAL PROCEDURES

Generation of Neural Progenitors from Human Induced Pluripotent Stem Cells

hiPSC (i90c17 and 16, passage 30–50; Nicoleau et al., 2013) amplification and neural progenitor cell generation, differentiation, and

immunostaining were performed as described in Supplemental Experimental Procedures.

α-Syn and HTTExon1Q48 Purification and Aggregation into Assemblies

Purification and quality control of human recombinant monomeric WT or S129A α-Syn and assembly into fibrils and ribbons and purification and assembly into fibrils of human recombinant HTTExon1Q48 were carried out as previously reported (Bousset et al., 2013) and described in Supplemental Experimental Procedures.

Biochemical Analysis

Cells cultured in classical 24-well plates were scraped into lysis buffer (20 mM Tris-HCl, pH 7.5; 0.8 M NaCl; 1 mM EGTA; 10% [w/v] sucrose; 1% sarkosyl) supplemented with protease (Roche) and phosphatase (Sigma) inhibitor cocktails and solubilized at 37°C for 30 min. Filter trap and insolubility assay were performed as previously reported (Bousset et al., 2013) and described in Supplemental Experimental Procedures.

SUPPLEMENTAL INFORMATION

Supplemental Information includes Supplemental Experimental Procedures, five figures, one table, and two videos and can be found with this article online at https://doi.org/10.1016/j.stemcr.2018.12.007.

AUTHOR CONTRIBUTIONS

S.G. completed hiPSC neuronal differentiation, microfluidic network reconstructions, α-Syn propagation assays, confocal analysis, and Ca^{2+} imaging analysis. P.T. performed microfluidic network reconstructions, α-Syn propagation assays, and biochemical analysis. L.B. produced and characterized α-Syn assemblies and performed biochemical analysis. A.F. performed quality control of assemblies. P.L. contributed to sample preparation. R.M. supervised the biochemical works. A.P. supervised the iPSC assay development. J-M.P. supervised the network reconstruction assay and α-Syn propagation experiments. S.G., P.T., R.M., J-M.P., and A.P, wrote the manuscript.

ACKNOWLEDGMENTS

We thank J.L. Viovy, C. Villard, and the MMBM's group at Institut Pierre Gilles de Gennes (IPGG) for their useful comments and help in microfluidic chips design and fabrication. This work was supported by grants from the European Commission: MicroDEG, ERA-NET Neuron JTC2012 "Novel Methods" (J-M.P. and A.L.P.), the CNRS (J-M.P.), Inserm (A.L.P.), the Laboratoire d'Excellence Revive (ANR-10-LABX-73, A.L.P.), NeurATRIS (ANR-11-INBS-0011, A.L.P.), INGESTEM (ANR-11-INBS-0009, A.L.P.), European Commission H2020 Project Joint Programme – Neurodegenerative Disease Research (JPND) ModelPolyQ grant 643417, A.L.P.). I-Stem is supported by the Association Française contre les Myopathies (AFM-Téléthon). We thank the IMAGIF facility for access to electron microscopes. L.B. and R.M. were supported by the EC Joint Program on Neurodegenerative Diseases (NeuTARGETs-ANR-14-JPCD-0002-02 and SYNACTION-ANR-15-JPWG-0012-03), a "Coup d'Elan à la Recherche Française" award from the Fondation Bettencourt Schueller, the Fondation de France (contract 2015–00060936) by Equipe FRM (Fondation pour la Recherche Médicale) 2016 (DEQ2016033489), the Innovative Medicine Initiative 2 grant agreement No 116060 (IMPRiND, www.im prind.org) supported by the European Union's Horizon 2020 research and innovation program and EFPIA, and the Fondation Simone et Cino Del Duca of the Institut de France. Dr. Peyrin is a founder and shareholder of MicrobrainBiotech.

Received: March 28, 2018
Revised: December 11, 2018
Accepted: December 11, 2018
Published: January 10, 2019

REFERENCES

Abounit, S., Bousset, L., Loria, F., Zhu, S., de Chaumont, F., Pieri, L., Olivo-Marin, J.C., Melki, R., and Zurzolo, C. (2016). Tunneling nanotubes spread fibrillar alpha-synuclein by intercellular trafficking of lysosomes. EMBO J. *35*, 2120–2138.

Apetri, M.M., Harkes, R., Subramaniam, V., Canters, G.W., Schmidt, T., and Aartsma, T.J. (2016). Direct observation of alpha-synuclein amyloid aggregates in endocytic vesicles of neuroblastoma cells. PLoS One *11*, e0153020.

Azeredo da Silveira, S., Schneider, B.L., Cifuentes-Diaz, C., Sage, D., Abbas-Terki, T., Iwatsubo, T., Unser, M., and Aebischer, P. (2009). Phosphorylation does not prompt, nor prevent, the formation of alpha-synuclein toxic species in a rat model of Parkinson's disease. Hum. Mol. Genet. *18*, 872–887.

Betemps, D., Verchere, J., Brot, S., Morignat, E., Bousset, L., Gaillard, D., Lakhdar, L., Melki, R., and Baron, T. (2014). Alpha-synuclein spreading in M83 mice brain revealed by detection of pathological alpha-synuclein by enhanced ELISA. Acta Neuropathol. Commun. *2*, 29.

Bousset, L., Pieri, L., Ruiz-Arlandis, G., Gath, J., Jensen, P.H., Habenstein, B., Madiona, K., Olieric, V., Bockmann, A., Meier, B.H., et al. (2013). Structural and functional characterization of two alpha-synuclein strains. Nat. Commun. *4*, 2575.

Braak, H., Alafuzoff, I., Arzberger, T., Kretzschmar, H., and Del Tredici, K. (2006). Staging of Alzheimer disease-associated neurofibrillary pathology using paraffin sections and immunocytochemistry. Acta Neuropathol. *112*, 389–404.

Braak, H., Del Tredici, K., Rub, U., de Vos, R.A., Jansen Steur, E.N., and Braak, E. (2003a). Staging of brain pathology related to sporadic Parkinson's disease. Neurobiol. Aging *24*, 197–211.

Braak, H., Rub, U., Gai, W.P., and Del Tredici, K. (2003b). Idiopathic Parkinson's disease: possible routes by which vulnerable neuronal types may be subject to neuroinvasion by an unknown pathogen. J. Neural Transm. (Vienna) *110*, 517–536.

Brahic, M., Bousset, L., Bieri, G., Melki, R., and Gitler, A.D. (2016). Axonal transport and secretion of fibrillar forms of alpha-synuclein, Abeta42 peptide and HTTExon 1. Acta Neuropathol. *131*, 539–548.

Brundin, P., and Melki, R. (2017). Prying into the prion hypothesis for Parkinson's disease. J. Neurosci. *37*, 9808–9818.

Deleglise, B., Lassus, B., Soubeyre, V., Alleaume-Butaux, A., Hjorth, J.J., Vignes, M., Schneider, B., Brugg, B., Viovy, J.L., and Peyrin, J.M. (2013). Synapto-protective drugs evaluation in reconstructed neuronal network. PLoS One *8*, e71103.

Deleglise, B., Magnifico, S., Duplus, E., Vaur, P., Soubeyre, V., Belle, M., Vignes, M., Viovy, J.L., Jacotot, E., Peyrin, J.M., et al. (2014). beta-Amyloid induces a dying-back process and remote trans-synaptic alterations in a microfluidic-based reconstructed neuronal network. Acta Neuropathol. Commun. *2*, 145.

Desplats, P., Lee, H.J., Bae, E.J., Patrick, C., Rockenstein, E., Crews, L., Spencer, B., Masliah, E., and Lee, S.J. (2009). Inclusion formation and neuronal cell death through neuron-to-neuron transmission of alpha-synuclein. Proc. Natl. Acad. Sci. U S A *106*, 13010–13015.

Di Maio, R., Barrett, P.J., Hoffman, E.K., Barrett, C.W., Zharikov, A., Borah, A., Hu, X., McCoy, J., Chu, C.T., Burton, E.A., et al. (2016). α-Synuclein binds to TOM20 and inhibits mitochondrial protein import in Parkinson's disease. Sci. Transl. Med. *8*, 342ra378.

Dinh, N.D., Chiang, Y.Y., Hardelauf, H., Baumann, J., Jackson, E., Waide, S., Sisnaiske, J., Frimat, J.P., van Thriel, C., Janasek, D., et al. (2013). Microfluidic construction of minimalistic neuronal co-cultures. Lab Chip *13*, 1402–1412.

Febbraro, F., Sahin, G., Farran, A., Soares, S., Jensen, P.H., Kirik, D., and Romero-Ramos, M. (2013). Ser129D mutant alpha-synuclein induces earlier motor dysfunction while S129A results in distinctive pathology in a rat model of Parkinson's disease. Neurobiol. Dis. *56*, 47–58.

Feyeux, M., Bourgois-Rocha, F., Redfern, A., Giles, P., Lefort, N., Aubert, S., Bonnefond, C., Bugi, A., Ruiz, M., Deglon, N., et al. (2012). Early transcriptional changes linked to naturally occurring Huntington's disease mutations in neural derivatives of human embryonic stem cells. Hum. Mol. Genet. *21*, 3883–3895.

Flavin, W.P., Bousset, L., Green, Z.C., Chu, Y., Skarpathiotis, S., Chaney, M.J., Kordower, J.H., Melki, R., and Campbell, E.M. (2017). Endocytic vesicle rupture is a conserved mechanism of cellular invasion by amyloid proteins. Acta Neuropathol. *134*, 629–653.

Freundt, E.C., Maynard, N., Clancy, E.K., Roy, S., Bousset, L., Sourigues, Y., Covert, M., Melki, R., Kirkegaard, K., and Brahic, M. (2012). Neuron-to-neuron transmission of alpha-synuclein fibrils through axonal transport. Ann. Neurol. *72*, 517–524.

Froula, J.M., Henderson, B.W., Gonzalez, J.C., Vaden, J.H., McLean, J.W., Wu, Y., Banumurthy, G., Overstreet-Wadiche, L., Herskowitz, J.H., and Volpicelli-Daley, L.A. (2018). alpha-Synuclein fibril-induced paradoxical structural and functional defects in hippocampal neurons. Acta Neuropathol. Commun. *6*, 35.

Gaspard, N., Bouschet, T., Herpoel, A., Naeije, G., van den Ameele, J., and Vanderhaeghen, P. (2009). Generation of cortical neurons from mouse embryonic stem cells. Nat. Protoc. *4*, 1454–1463.

George, S., Rey, N.L., Reichenbach, N., Steiner, J.A., and Brundin, P. (2013). alpha-Synuclein: the long distance runner. Brain Pathol. *23*, 350–357.

Goedert, M., Spillantini, M.G., Del Tredici, K., and Braak, H. (2013). 100 years of Lewy pathology. Nat. Rev. Neurol. *9*, 13–24.

Gorbatyuk, O.S., Li, S., Sullivan, L.F., Chen, W., Kondrikova, G., Manfredsson, F.P., Mandel, R.J., and Muzyczka, N. (2008). The phosphorylation state of Ser-129 in human alpha-synuclein determines neurodegeneration in a rat model of Parkinson disease. Proc. Natl. Acad. Sci. U S A *105*, 763–768.

Grassi, D., Howard, S., Zhou, M., Diaz-Perez, N., Urban, N.T., Guerrero-Given, D., Kamasawa, N., Volpicelli-Daley, L.A., LoGrasso, P., and Lasmezas, C.I. (2018). Identification of a highly neurotoxic alpha-synuclein species inducing mitochondrial damage and mitophagy in Parkinson's disease. Proc. Natl. Acad. Sci. U S A *115*, E2634–E2643.

Grskovic, M., Javaherian, A., Strulovici, B., and Daley, G.Q. (2011). Induced pluripotent stem cells—opportunities for disease modelling and drug discovery. Nat. Rev. Drug Discov. *10*, 915–929.

Guo, J.L., Covell, D.J., Daniels, J.P., Iba, M., Stieber, A., Zhang, B., Riddle, D.M., Kwong, L.K., Xu, Y., Trojanowski, J.Q., et al. (2013). Distinct alpha-synuclein strains differentially promote tau inclusions in neurons. Cell *154*, 103–117.

Hansen, C., Angot, E., Bergstrom, A.L., Steiner, J.A., Pieri, L., Paul, G., Outeiro, T.F., Melki, R., Kallunki, P., Fog, K., et al. (2011). alpha-Synuclein propagates from mouse brain to grafted dopaminergic neurons and seeds aggregation in cultured human cells. J. Clin. Invest. *121*, 715–725.

Jellinger, K.A. (2012). Neuropathology of sporadic Parkinson's disease: evaluation and changes of concepts. Mov. Disord. *27*, 8–30.

Kaufmann, T.J., Harrison, P.M., Richardson, M.J., Pinheiro, T.J., and Wall, M.J. (2016). Intracellular soluble alpha-synuclein oligomers reduce pyramidal cell excitability. J. Physiol. *594*, 2751–2772.

Kordower, J.H., Chu, Y., Hauser, R.A., Freeman, T.B., and Olanow, C.W. (2008). Lewy body-like pathology in long-term embryonic nigral transplants in Parkinson's disease. Nat. Med. *14*, 504–506.

Le Dur, A., Lai, T.L., Stinnakre, M.G., Laisne, A., Chenais, N., Rakotobe, S., Passet, B., Reine, F., Soulier, S., Herzog, L., et al. (2017). Divergent prion strain evolution driven by PrP(C) expression level in transgenic mice. Nat. Commun. *8*, 14170.

Lee, H.J., Suk, J.E., Bae, E.J., Lee, J.H., Paik, S.R., and Lee, S.J. (2008). Assembly-dependent endocytosis and clearance of extracellular alpha-synuclein. Int. J. Biochem. Cell Biol. *40*, 1835–1849.

Li, J.Y., Englund, E., Holton, J.L., Soulet, D., Hagell, P., Lees, A.J., Lashley, T., Quinn, N.P., Rehncrona, S., Bjorklund, A., et al. (2008). Lewy bodies in grafted neurons in subjects with Parkinson's disease suggest host-to-graft disease propagation. Nat. Med. *14*, 501–503.

Loria, F., Vargas, J.Y., Bousset, L., Syan, S., Salles, A., Melki, R., and Zurzolo, C. (2017). alpha-Synuclein transfer between neurons and astrocytes indicates that astrocytes play a role in degradation rather than in spreading. Acta Neuropathol. *134*, 789–808.

Luk, K.C., Kehm, V., Carroll, J., Zhang, B., O'Brien, P., Trojanowski, J.Q., and Lee, V.M. (2012a). Pathological alpha-synuclein transmission initiates Parkinson-like neurodegeneration in nontransgenic mice. Science *338*, 949–953.

Luk, K.C., Kehm, V.M., Zhang, B., O'Brien, P., Trojanowski, J.Q., and Lee, V.M. (2012b). Intracerebral inoculation of pathological alpha-synuclein initiates a rapidly progressive neurodegenerative alpha-synucleinopathy in mice. J. Exp. Med. *209*, 975–986.

Masuda-Suzukake, M., Nonaka, T., Hosokawa, M., Oikawa, T., Arai, T., Akiyama, H., Mann, D.M., and Hasegawa, M. (2013). Prion-like

spreading of pathological alpha-synuclein in brain. Brain *136*, 1128–1138.

Melki, R. (2015). Role of different alpha-synuclein strains in synucleinopathies, similarities with other neurodegenerative diseases. J. Parkinsons Dis. *5*, 217–227.

Nicoleau, C., Varela, C., Bonnefond, C., Maury, Y., Bugi, A., Aubry, L., Viegas, P., Bourgois-Rocha, F., Peschanski, M., and Perrier, A.L. (2013). Embryonic stem cells neural differentiation qualifies the role of Wnt/beta-catenin signals in human telencephalic specification and regionalization. Stem Cells *31*, 1763–1774.

Peelaerts, W., Bousset, L., Van der Perren, A., Moskalyuk, A., Pulizzi, R., Giugliano, M., Van den Haute, C., Melki, R., and Baekelandt, V. (2015). alpha-Synuclein strains cause distinct synucleinopathies after local and systemic administration. Nature *522*, 340–344.

Peng, C., Gathagan, R.J., Covell, D.J., Medellin, C., Stieber, A., Robinson, J.L., Zhang, B., Pitkin, R.M., Olufemi, M.F., Luk, K.C., et al. (2018). Cellular milieu imparts distinct pathological alpha-synuclein strains in alpha-synucleinopathies. Nature *557*, 558–563.

Peyrin, J.M., Deleglise, B., Saias, L., Vignes, M., Gougis, P., Magnifico, S., Betuing, S., Pietri, M., Caboche, J., Vanhoutte, P., et al. (2011). Axon diodes for the reconstruction of oriented neuronal networks in microfluidic chambers. Lab Chip *11*, 3663–3673.

Pieri, L., Chafey, P., Le Gall, M., Clary, G., Melki, R., and Redeker, V. (2016). Cellular response of human neuroblastoma cells to alpha-synuclein fibrils, the main constituent of Lewy bodies. Biochim. Biophys. Acta *1860*, 8–19.

Prots, I., Grosch, J., Brazdis, R.M., Simmnacher, K., Veber, V., Havlicek, S., Hannappel, C., Krach, F., Krumbiegel, M., Schutz, O., et al. (2018). alpha-Synuclein oligomers induce early axonal dysfunction in human iPSC-based models of synucleinopathies. Proc. Natl. Acad. Sci. U S A *115*, 7813–7818.

Reeve, A.K., Ludtmann, M.H., Angelova, P.R., Simcox, E.M., Horrocks, M.H., Klenerman, D., Gandhi, S., Turnbull, D.M., and Abramov, A.Y. (2015). Aggregated alpha-synuclein and complex I deficiency: exploration of their relationship in differentiated neurons. Cell Death Dis. *6*, e1820.

Rutherford, N.J., Brooks, M., and Giasson, B.I. (2016). Novel antibodies to phosphorylated alpha-synuclein serine 129 and NFL serine 473 demonstrate the close molecular homology of these epitopes. Acta Neuropathol. Commun. *4*, 80.

Sacino, A.N., Brooks, M., Thomas, M.A., McKinney, A.B., Lee, S., Regenhardt, R.W., McGarvey, N.H., Ayers, J.I., Notterpek, L., Borchelt, D.R., et al. (2014a). Intramuscular injection of alpha-synuclein induces CNS alpha-synuclein pathology and a rapid-onset motor phenotype in transgenic mice. Proc. Natl. Acad. Sci. U S A *111*, 10732–10737.

Sacino, A.N., Brooks, M., Thomas, M.A., McKinney, A.B., McGarvey, N.H., Rutherford, N.J., Ceballos-Diaz, C., Robertson, J., Golde, T.E., and Giasson, B.I. (2014b). Amyloidogenic alpha-synuclein seeds do not invariably induce rapid, widespread pathology in mice. Acta Neuropathol. *127*, 645–665.

Sacino, A.N., Brooks, M.M., Chakrabarty, P., Saha, K., Khoshbouei, H., Golde, T.E., and Giasson, B.I. (2016). Proteolysis of alpha-synuclein fibrils in the lysosomal pathway limits induction of inclusion pathology. J. Neurochem. *140*, 662–678.

Salvesen, L., Winge, K., Brudek, T., Agander, T.K., Lokkegaard, A., and Pakkenberg, B. (2017). Neocortical neuronal loss in patients with multiple system atrophy: a stereological study. Cereb. Cortex *27*, 400–410.

Spillantini, M.G., Schmidt, M.L., Lee, V.M., Trojanowski, J.Q., Jakes, R., and Goedert, M. (1997). Alpha-synuclein in Lewy bodies. Nature *388*, 839–840.

Sugiura, K., Hashizume, Y., Kume, A., and Takahashi, A. (1995). Distribution of neuronal cytoplasmic inclusions in multiple system atrophy. Nagoya J. Med. Sci. *58*, 117–126.

Tenreiro, S., Eckermann, K., and Outeiro, T.F. (2014). Protein phosphorylation in neurodegeneration: friend or foe? Front. Mol. Neurosci. *7*, 42.

Uchihara, T., and Giasson, B.I. (2016). Propagation of alpha-synuclein pathology: hypotheses, discoveries, and yet unresolved questions from experimental and human brain studies. Acta Neuropathol. *131*, 49–73.

Volpicelli-Daley, L.A., Luk, K.C., and Lee, V.M. (2016). Addition of exogenous alpha-synuclein preformed fibrils to primary neuronal cultures to seed recruitment of endogenous alpha-synuclein to Lewy body and Lewy neurite-like aggregates. Nat. Protoc. *9*, 2135–2146.

Volpicelli-Daley, L.A., Luk, K.C., Patel, T.P., Tanik, S.A., Riddle, D.M., Stieber, A., Meaney, D.F., Trojanowski, J.Q., and Lee, V.M. (2011). Exogenous alpha-synuclein fibrils induce Lewy body pathology leading to synaptic dysfunction and neuron death. Neuron *72*, 57–71.

Yau, Y., Zeighami, Y., Baker, T.E., Larcher, K., Vainik, U., Dadar, M., Fonov, V.S., Hagmann, P., Griffa, A., Misic, B., et al. (2018). Network connectivity determines cortical thinning in early Parkinson's disease progression. Nat. Commun. *9*, 12.

Stem Cell Reports, Volume *12*

Supplemental Information

Propagation of α-Synuclein Strains within Human Reconstructed Neuronal Network

Simona Gribaudo, Philippe Tixador, Luc Bousset, Alexis Fenyi, Patricia Lino, Ronald Melki, Jean-Michel Peyrin, and Anselme L. Perrier

Supplemental information

SUPPLEMENTAL ITEMS

Supplemental video 1 and 2. *Axonal Transport of α-Syn ribbons (video1) or fibrils (video2) visualized at the exit of the microgrooves in the distal chamber*, Related to Figure 2. Thirty days in vitro reconstructed human cortical neuronal networks were loaded in the proximal chamber with 500 nM Atto-550 labelled ribbons or fibrils. The movements of the fluorescent assemblies were then imaged in the distal chamber, at the exit of the microgrooves, 24h after exposure. Fluorescent images were acquired every 600 ms for 5 min and were superimposed on a phase-contrast image acquired at the beginning of the acquisition (scale bar: 50 µm; timestamp: sec).

SUPPLEMENTAL FIGURES

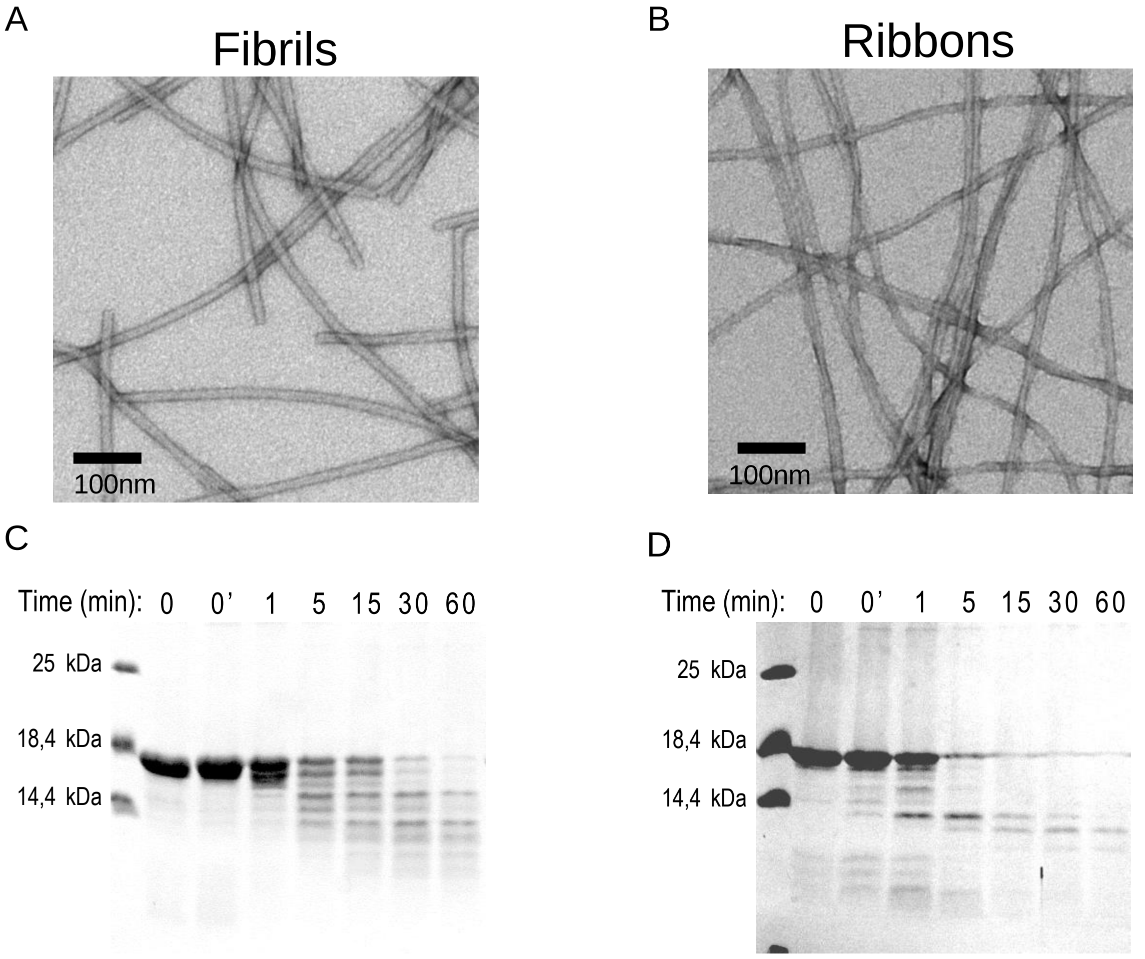

Figure S1. *Quality control and characterization of fibrils and ribbons*, Related to Figure 2-7. Fibrils and ribbons were generated exactly as described in Bousset et al., 2013 and as described in the materials and methods section. The resulting assemblies were adsorbed on 200 mesh carbon coated electron microscopy grids and imaged by transmission electron microscopy after negative staining (A and B). Besides differing through their shapes, the polymorphs/strains fibrils (cylindrical) and ribbons (flat) differ through their limited proteolytic patterns. Thus, the resulting fibrils (C) and ribbons (D) (mg/ml) were subjected to degradation by proteinase K. Aliquots were removed before (0), immediately (0') or at the indicated time (in minutes) after addition of proteinase K, denatured in boiling Laemmli buffer for 5 minutes at 90°C and subjected to SDS-PAGE on 12% polyacrylamide gels. The Coomassie stains of the SDS-PAGE are shown (C and D). The patterns in C and D are

characteristic of the polymorphs/strains fibrils and ribbons, respectively (Bousset et al., 2013). The pre-stained molecular weight markers are shown on the left.

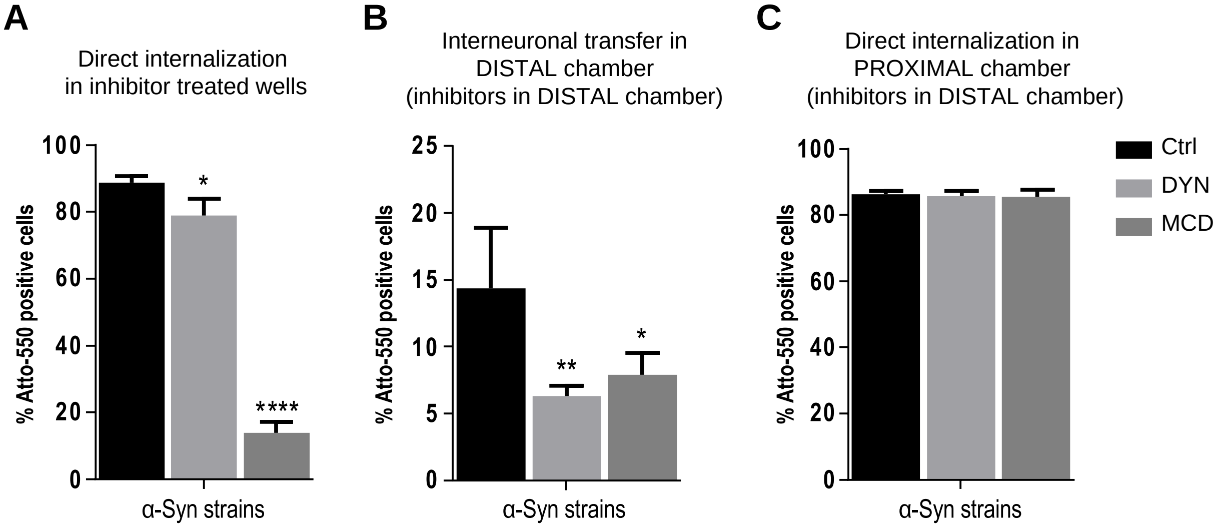

Figure S2. *Inhibition of receptor/clathrin-dependent endocytosis or fission of endocytic vesicles reduces the direct and indirect internalization of α-Syn strains,* Related to Figure 2. A-C: Before strains addition, neurons were pre-treated for 1 hour with Monocadaverin (MCD, 70 µM) or Dynasore (DYN, 80 µM) and analysis were performed 24 hours later. A: The direct uptake of Atto-550 α-Syn strains (fibrils and ribbons) from the medium was strongly reduced by application of MCD while application of DYN only partially reduced the uptake of the assemblies. B-C: taking advantage of the microfluidic system, the effect of MCD and DYN was tested on the capacity of secondary neurons located in the distal chamber to uptake Atto-550 strains from axon of “donor” neurons. B: Both MCD and DYN application reduced the proportion of neurons in the distal chamber with internalized exogenous assemblies. C: the proportion of Atto-positive neurons in the proximal chamber did not change during inhibitors application in the distal chamber (F=473.6 in A, F=8.10 in B, ANOVA with Dunnett’s post-hoc tests). * represents $p < 0.05$, ** and °° $p<0.01$, **** $p < 0.0001$. Data are mean ± SEM, n=3 independent experiments.

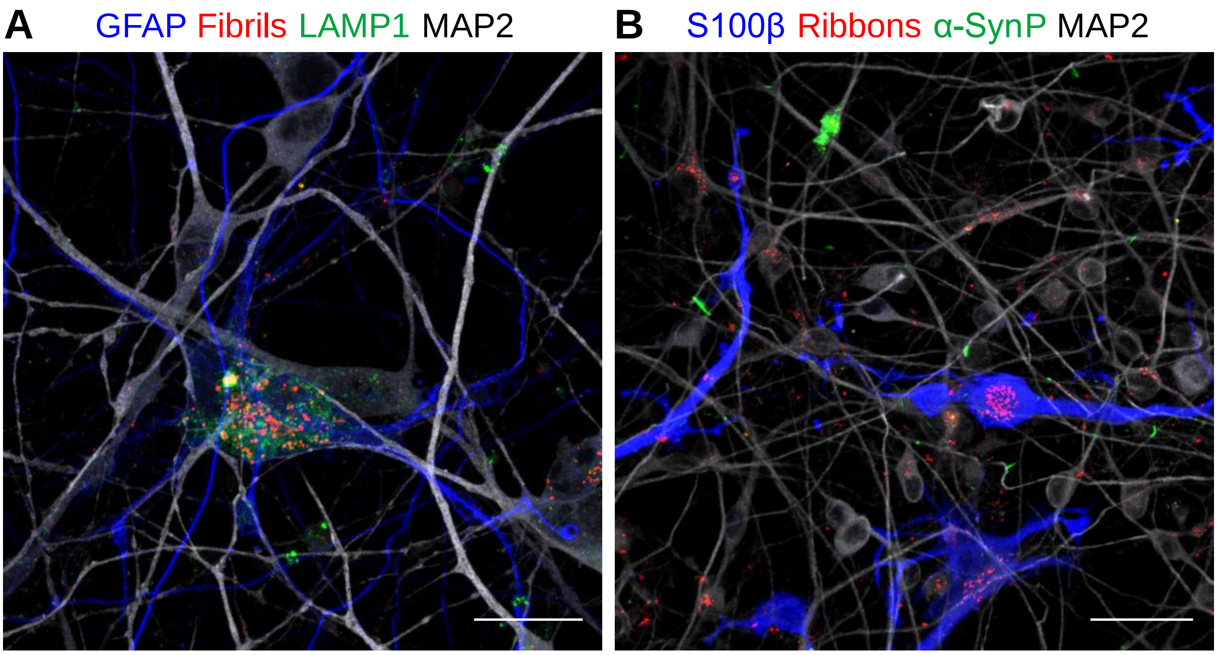

Figure S3. *Immunofluorescence analysis of human glial cells exposed to α-Syn assemblies*, Related to Figure 3. A: Fluorescence confocal imaging of LAMP1 (green) in human cortical culture (MAP2, grey) 30 days after exposure to 500 nM Atto-550 labelled (red) α-Syn strains. Representative images showing that Atto-550 assemblies in lysosome (LAMP1+) of GFAP+ (blue) glial cells. B: Fluorescence confocal imaging of endogenous α-SynP (green) in human cortical culture (MAP2, grey) 30 days after exposure to 500 nM Atto-550 labelled (red) α-Syn ribbons. Representative images showing that α-SynP inclusions are not found in S100β-positive (blue) glial cells. Scale bar 20 µm in A, 40 µm in B.

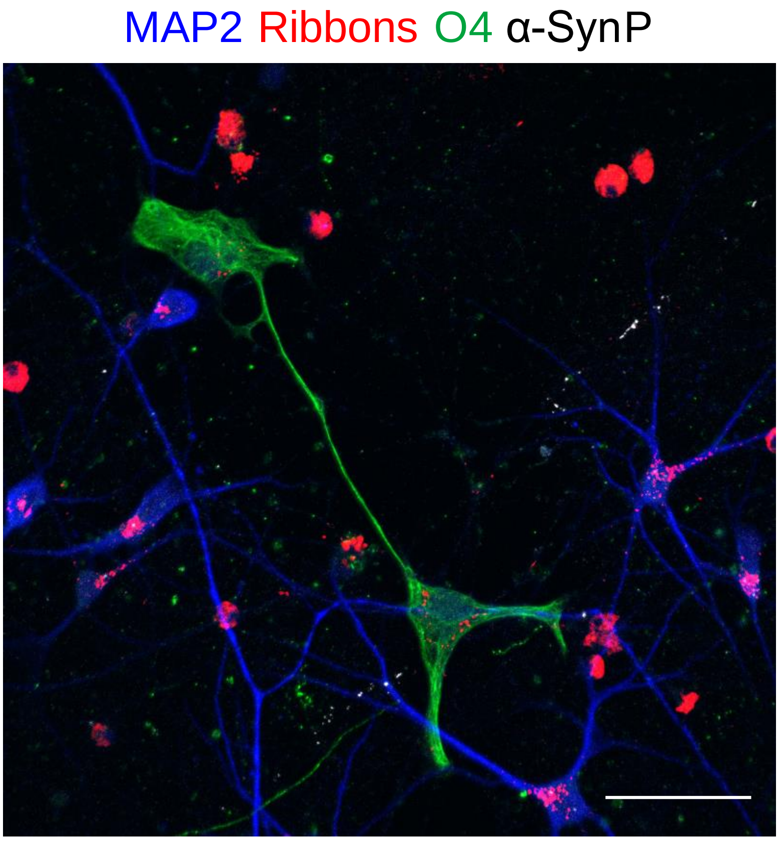

Figure S4. *Immunofluorescence analysis of human neuronal cultures containing O4-positive oligodendrocytes precursor cells exposed to α-Syn assemblies,* Related to Figure 3. Fluorescence confocal imaging of O4 (green) in human cortical culture (MAP2, blue) 21 days after exposure to 500 nM Atto-550 labelled (red) α-Syn fibrillar assemblies. Representative images showing the presence of Atto-550 assemblies in O4 positive cells; α-SynP (grey) inclusions are never found in O4-positive (green) cells. Scale bar 40 µm

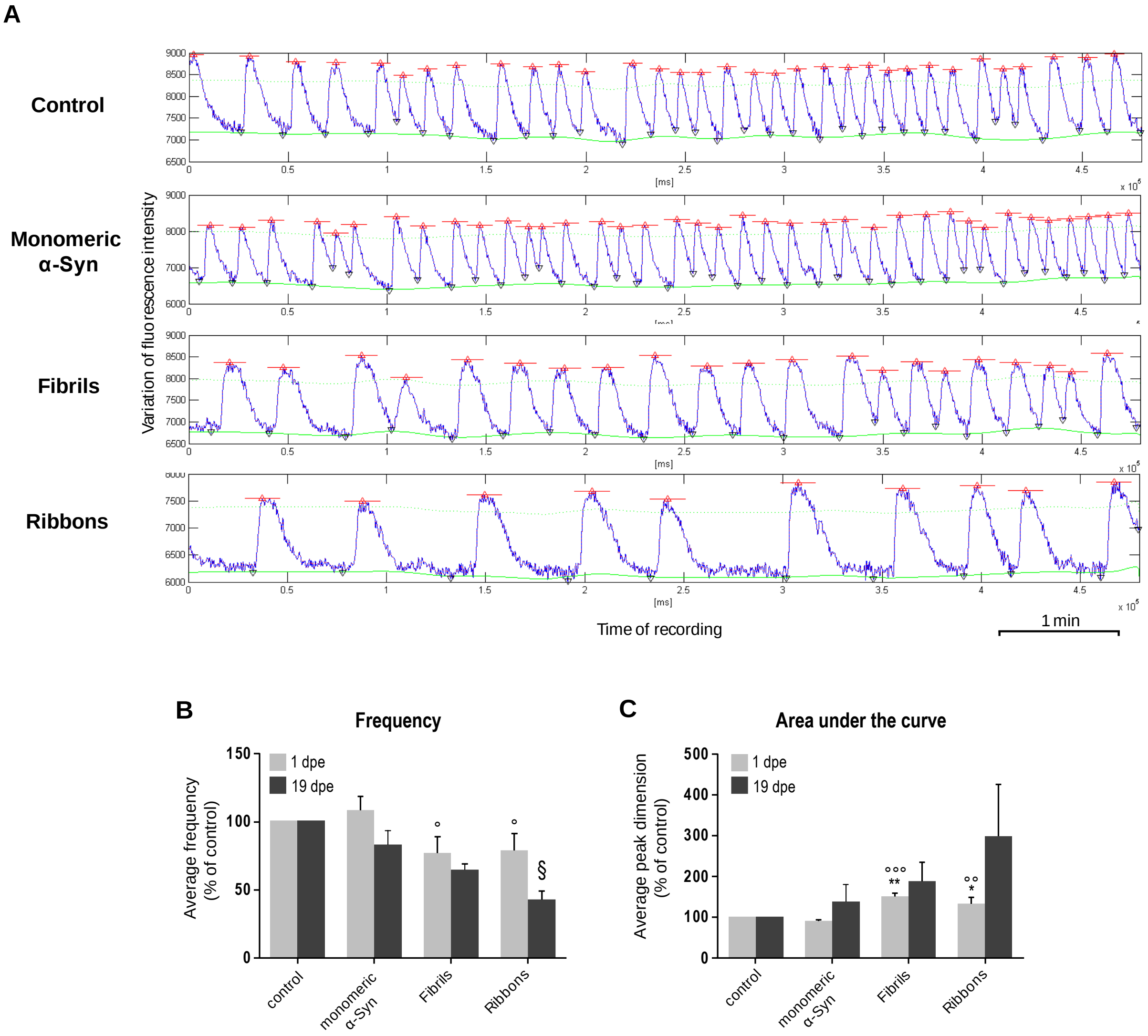

Figure S5. *Alterations of Ca^{2+} oscillations in human neuronal cultures treated with exogenous α-Syn assemblies*, Related to Figure 6. A: Representatives traces of spontaneous Ca^{2+} oscillations obtained by whole well recording followed by FDSS waveform analysis of Fluo4 loaded cortical human neuronal cultures. Cells were treated at 5 weeks and Ca^{2+} recording was performed 19 days post exposure (dpe) to monomeric α-Syn, fibrils, and ribbons. Control cells were unexposed. B-C: Average frequency (B) and average peak dimension (C, area under the curve) in mature neuronal networks (recording performed at 8 weeks) at 1 or 19 dpe. Already at 1 dpe, ribbons and fibrils exposure triggered decrease in frequency (B) and increase in the AUC (C) of Ca^{2+} oscillation in human neuronal cultures (F=8.36 in B, F=28.65 in C, ANOVA with Tukey's post-hoc tests). Stars indicate significant difference of 1 dpe treated neurons vs control; empty dots indicate significant difference of 1 dpe treated neurons vs monomeric α-Syn. * and ° represent p< 0.05, ** and °° p<0.01, °°° p < 0.001. These variations tend to amplify with time. In particular decrease in peak frequency between 1 and 19 dpe (B) is significant in ribbons-treated neuronal cultures (F=8.113, p=0.0015, ANOVA with Sidak's post-hoc test, indicated by the paragraph sign). Data are presented as mean percentage compared to control ± SEM, n=3 independent experiments for each exposure time.

SUPPLEMENTAL EXPERIMENTAL PROCEDURES

Microfluidic chips fabrication

Three compartmented microfluidic chambers were designed and produced as previously described (Deleglise et al., 2013). After bonding PDMS chips to glass coverslip with a plasma generator, the chips were coated with a solution of poly-Ornithine overnight (0.002% final concentration, P4957 Sigma; St. Louis, MO, USA). Chips were then washed in PBS and further coated with Laminin (4µg/ml final concentration, 23017015, Invitrogen) for at least 4 hours.

Neural induction and differentiation of hiPSCs

The i90c17 and 16 human iPS cells line (passage 30-50 (Nicoleau et al., 2013)) were maintained on L7 (Lonza) matrix and grown in STEMPRO medium (Invitrogen) in presence of FGF2 (10 ng/ml, Peprotech). Cultures were fed daily and manually passaged every 4–5 days. For neural differentiation, hiPSC colonies were treated as previously described (Nicoleau et al., 2013). N2B27 medium was supplemented with 0.1 µM LDN-193189 (Sigma), 20 µM SB-431542 (Tocris), 1 µM XAV-939 (Tocris) for the first 10 days and with 1 µM XAV-939 only starting from day 10. At day 18, cells were enzymatically dissociated using Accutase (Invitrogen) and further expanded in N2B27 supplemented with FGF2 (20ng/ml), EGF (10 ng/ml, R&D systems) and BDNF (10 ng/ml Peprotech). Cells were passaged up to 6 times every 3-4 days. For banking, neuronal precursor (NSC) were resuspended at $8x10^6$ cells/ml in “freezing medium” (90% fetal calf serum 10% DMSO), frozen and stored in liquid nitrogen vapor at -150°C.

Neuronal differentiation of hiPSC-derived NSCs

NSC were suspended in N2B27 medium to a final density of $80x10^6$ cells/mL for the proximal compartment and $20x10^6$ cells/ml for the distal compartment into poly-L-ornithine/laminin coated microfluidic chips or at 10^6 cells/ml for culture in regular culture plate. For the final differentiation cells were cultivated in presence of BDNF (20 ng/ml), cAMP (100 µM, Sigma) and valproic acid (0.5 mM, Sigma). DAPT (5 µM, Tocris) was added to the culture for the first 2 weeks. Medium was change every 5 days.

Immunofluorescence

Cells were fixed with 4% PFA/4% sucrose and further permeabilized with 0.1% Triton X-100 and 2% BSA in PBS. To assess α-Syn detergent insolubility by immunofluorescence, samples were alternatively fixed with 4% PFA in presence of 1% Triton X-100 as previously described (Volpicelli-Daley et al., 2016). Primary antibodies (listed in Table S1) were then added and the samples incubated at 4°C overnight in PBS. Species specific secondary antibodies coupled to Alexa 350, 488, 555 and 647 (1/1000, Invitrogen) and DAPI counterstain were applied for 1 hour at room temperature.

Image acquisition and analysis

Confocal analyses were performed using a ZEISS LSM 880 Confocal Laser Scanning Microscope with Airyscan module (Zen software). Quantitative and live imaging analyses were acquired with an Axio-observer Z1 (Zeiss) fitted with a cooled CCD camera (ORCA-Flash4, Hamamatsu, Coolsnap HQ2, Roper Scientific, Metamorph software (Molecular Imaging)) for widefield fluorescence acquisition or with an Evolve EMCCD camera (Zeiss), coupled to a spinning disk system (Nipkow, CSU-X1M 5000, Zeiss). Quantitative immunofluorescence analysis was performed on randomly selected visual fields from three independent microfluidic devices using ImageJ. On average, 3 visual fields were acquired per each microfluidic chamber and a total of 50 cells were counted per field. Data are presented as mean ± SEM. The quantification of α-SynP in neurons was estimated by the ratio between the area occupied by α-SynP and MAP2 in the somatic and proximal dendrites compartments of neurons exposed to either fibrils or ribbons. Data are presented as mean ± SD.

α-Syn and HTTExon 1Q48 purification and aggregation into assemblies

A variant human α-Syn where Serine 129 residue was changed to Alanine (S129A α-Syn) was generated by site directed mutagenesis. This variant cannot be phosphorylated in neurons on S129, the main phosphorylation site for α-Syn. Purification and quality control of human recombinant monomeric WT or S129A α-Syn and assembly into fibrils and ribbons were carried out as previously described (Bousset et al., 2013). The purification and

assembly into fibrils of human recombinant HTTExon1Q48 was performed as described (Monsellier et al., 2015). WT and S129A α-Syn fibrils, ribbons and HTTExon1Q48, preformed fibrils were pelleted and washed with PBS buffer, pH 7.4 and further labelled with a. two molar excess of NHS-ester ATTO 550 (AttoTec) according to manufacturer's recommendations. Preformed assemblies were fragmented by sonication for 20 min in 2-ml Eppendorf tubes in a Vial Tweeter powered by an ultrasonic processor UIS250v (250 W, 2.4 kHz; Hielscher Ultrasonic, Teltow, Germany). Following labelling, the unreacted dye was removed using a desalting column (PD10, GE Healthcare) equilibrated in PBS buffer, pH 7,4.
For transmission electron microscopy, the assemblies were adsorbed on 200 mesh carbon coated electron microscopy grids and imaged after negative staining. For degradation by proteinase K, aliquots of fibrils and ribbons (mg/ml) were removed before (0), immediately (0') or at the indicated time (in minutes) after addition of proteinase K, denatured in boiling Laemmli buffer for 5 minutes at 90°C, subjected to SDS-PAGE on 12% polyacrylamide gels and stained by Comassie coloration.

Exposure of human neuronal cells to monomeric α-Syn, α-Syn strains and fibrillar HTTExon1Q48

Thirty days after seeding, neuronal cultures in plates or microfluidics were exposed to 500 nM of Atto550 labelled α-Syn fibrils, ribbons and monomers and to fibrillar HTTExon1Q48 with a 24 hours pulse, and then washed. The occurrence of de novo formation of pathological α-Syn was then assessed by biochemical and immunofluorescence approaches at 7, 15 and 30 days post-exposure to the different samples. For longitudinal analysis of exogenous assemblies transport/transfer, proximal chambers were loaded with 500 nM of Atto550 labelled α-Syn fibrils, ribbons and monomers with a 24 hours pulse. To prevent passive diffusion of assemblies, hydrostatic pressure was applied by adding medium excess in the reservoirs of recipient chambers. Loaded networks were imaged 1, 7, 15 and 21 days after addition of assemblies. To analyze axonal transport, time lapse imaging (5 minutes films, with time interval of 600 ms) was performed 24 hours post loading with an inverted Zeiss spinning disk system. Enhanced projections of the video sequences to identify and track axonal/fluorescent punctae paths were performed. For each path/axon segment, a kymograph representation was extracted with ImageJ software plug-In "Multiple Kymograph" and average velocity pattern as well as average path lengths of fluorescent punctae were then determined.

Analysis of endocytosis inhibitors

The contribution of receptor/clathrin-dependent endocytosis (Schlegel et al., 1982) or fission of endocytic vesicles (Macia et al., 2006) to the direct uptake of α-Syn strains by human cortical neurons was assessed. To this end, cells in classic p24 vessel were pretreated for 1 hour with 70 µM monodansylcadaverine (Sigma-Aldrich) or 80 µM dynasore (Tocris Bioscience Bristol, UK) and imaged 24 h after their exposure to Atto-550 Ribbons or Fibrils, at 500 nM, after washing. To analyze the effect of those two pharmacological inhibitors on the interneuronal transfer of fibrils and ribbons, our microfluidic setup was used. The neurons resident in the distal chamber (acceptor neurons) were pretreated with monodansylcadaverine or dynasore for 1 hour before exposing the cells in the proximal chamber (donor neurons) to 0.8 µM of fibrils or ribbon, in order to maximize the % of spreading between the neuronal networks. The cells in the 2 chamber were imaged 24 hours later.

Biochemical Analysis

Cells cultured in classic 24 well plates were scraped into lysis buffer (LB; 20mM Tris-HCl pH 7.5; 0,8M NaCl; 1mM EGTA; 10% (w/v) sucrose; 1% sarkosyl) supplemented with protease (Roche) and phosphatase (Sigma) inhibitor cocktails and solubilized at 37°C for 30 min. Lysates were then precleared by centrifugation at 1000 g for 10 min and the supernatants were retained for further analyses. Filter trap and insolubility assay were performed as previously described (Bousset et al., 2013). The primary antibodies used are listed in Table 1. After 4 washes with TBS-T, the membranes were further incubated for 1h with appropriate HRP conjugated secondary antibody diluted (1: 2000) in blocking buffer and then washed 4 times with TBS-T. Immunoblots were revealed with West Femto ECL (Pierce). Chemiluminescence was recorded on LAS-3000 imaging system (FUJIFILM) or Chemidoc (Bio-Rad) and quantified with image J.

Ca2+ imaging analysis

Human cortical neurons were cultured in 384 well plates at a density of 80.000 cells per cm^2. For Ca^{2+} imaging experiments, cells were loaded for 15 minutes with Fluo4 probe (1 µM, Invitrogen) in HBSS physiological solution then washed 3 times. For both 1 and 19 dpe studies, Ca^{2+} imaging recording were performed on human neuronal cultures at 8 weeks of final differentiation (5 weeks + 19 days, or 8 weeks + 1 day). Spontaneous Ca^{2+} oscillations were recorded using the FDSS 600 Hamamatsu imaging-based plate reader, for duration of 8

minutes at a time interval of 0.4 seconds. Analyses of the frequency, amplitude and area under the curve of the Ca^{2+} oscillations for each well were performed using the FDSS Waveform analysis software.

Analysis of mitochondria morphologies

For mitochondria morphological analysis, neuronal culture in µfluidic devices were treated at 4 weeks of differentiation and analyzed 21 days after exposure to fibrils, ribbons or monomeric α-Syn. Images were acquired by confocal microscope. For ribbons and fibrils treated neurons, cells were selected based on the presence of α-SynP somatic inclusion (α-SynP+ cells). The α-SynP– cells belonging to the same culture and surrounding the α-SynP+ neurons (e.g. not presenting α-SynP somatic inclusion but loaded with Atto-550 fibrils or ribbons) were analyzed separately. For unexposed neurons or neurons exposed to monomeric α-Syn, cells were acquired randomly. For the morphological analysis, for each cell (96 for RIB, 84 for FIB, 90 for monomeric and 65 for ctrl, from n=3 independent experiments) a score was assigned based on the MTCO2 staining, MAP2 used to identify the neurons. The score range from 0 to 3 for each category that include i) fusion, ii) fragmented/fission and iii) donut-shaped/condensed mitochondria. Classified cells were subsequently evaluated on α-SynP staining, and further distinguished in α-SynP+ or α-SynP-.

Statistical analyses

Figure 2A and 2B, *Interneuronal spreading of α-Syn assemblies*:

Two-way ANOVA: for proximal chamber (Fig 2A), F=40.50 (interaction), F=489.7 (treatment); for distal chamber (Fig 2B), F=6.237 (interaction), F=97.74 (treatment). Data are presented as mean ± SD, n=4 independent experiments. Multiple pairs of conditions show significantly different % of Atto-550 positive cells in proximal and distal chamber (Tukey's post-hoc tests):

Distal chamber (2B):

- Time effect: fibrils 1 vs 15 and 21 dpe (p<0.0001), 7 vs 15 and 21 (p<0.001); ribbons 1 vs 7 (p<0.01), 1 vs 15 and 21 (p<0.0001), 7 vs 15 and 21 (p<0.0001).
- Strains effect: fibrils and ribbons vs monomeric α-Syn at 1 dpe (p<0.01), 7, 15 and 21 dpe (p<0.0001); fibrils vs ribbons never significant.

Proximal chamber (2A):

- Time effect: monomeric α-Syn 1 vs 7, 15, 21 (p<0.0001), 7 vs 15 (p<0.5) and 21 (p<0.01); fibrils never significant; ribbons 1 vs 15 and 21 (p<0.01), 7 vs 15 and 21 (p<0.01).
- Strains effect: fibrils and ribbons vs monomeric α-Syn at 1, 7, 15, 21 dpe (p<0.001); fibrils vs ribbons never significant.

Figure 5C, *Time dependent effect of α-Syn strains exposure*:

Two-way ANOVA, F=16.55 (interaction), F=24.98 (treatment). Data are presented as mean ± SD, n=3 independent experiments. Multiple pairs of conditions show significant increase of the ratio between α-SynP area/MAP2 area over time for the 2 strains (Tukey's post-hoc tests):

- Time effect: fibrils 7 dpe vs 30 dpe (p<0.05); ribbons 7 or 15 dpe vs 30 dpe (p<0.001).
- Strain's effect (at 30 dpe): fibrils vs control (p<0.05), ribbons vs control (p<0.001), ribbons vs fibrils (p<0.001).

Figure 6A-C, *Alteration in calcium homeostasis:*

ANOVA, F=23.83 in A, F=7.14 in C, with Tukey's post-hoc tests. Stars indicate significant difference of α-Syn strains treated neurons vs control; empty dots indicate significant difference of α-Syn strains treated neurons vs monomeric α-Syn. Data are presented as mean ± SEM, 4 wells per experiment, n=3 independent experiments.

Figure 6D-E, *Alteration in mitochondria morphologies:*

For each cell (96 for RIB, 84 for FIB, 90 for monomeric and 65 for ctrl, from n=3 independent experiments, data are presented as mean ± SEM), a score was assigned (from 0 to 3) to each category and the cells were subsequently classified as α-SynP+ or α-SynP-.

- Comparison of treated neurons vs control: ANOVA, F=4.47 in D, F= 5.64 in E, F=5.60 in F, with Dunnett's post-hoc tests; differences are indicated by stars.
- Comparison of α-SynP+ vs α-SynP- cells present in the same culture: ANOVA, F=3.80 in D, F= 8.86 in E, F=5.11 in F, with Sidak's post-hoc tests; differences are indicated by empty dots.

Table S1. *List of primary antibodies*, Related to Figure 1-7.

Primary antibodies	Dilution IF	Dilution WB	Species	Provider	Reference number
**BIII-TUB**	1/1000		ms monoclonal	BioLegend	MMS-435P
**BIII-TUB**	1/1000		chick polyclonal	Aves Labs	---
**BRN2**	1/800		goat monoclonal	Santa Cruz Biotechnology	sc-6029
**CTIP2**	1/500		rat monoclonal	abcam	ab18465
**CUX2**	1/2000		rb polyclonal	abcam	ab130395
**GABA**	1/1000		rb polyclonal	Sigma	A2052
**GFAP**	1/5000		rb polyclonal	DakoCytomatic	Z0334
**HSP70**	1/200		ms monoclonal	ThermoFisher Scientific	Clone 4G4, MA3-009
**LAMP1**	1/300		ms monoclonal	Abcam	ab25630
**LAMP2**	1/300		ms monoclonal	Abcam	ab25631
**MAP2**	1/500		ms monoclonal	Sigma	M1406
**MAP2**	1/500		chick polyclonal	BioLegend	822501
**MTCO2**	1/400		ms monoclonal	Abcam	ab3298
**phospho S129 α-Syn**	1/500		rb polyclonal	Abcam	ab59264
**phospho S129 α-Syn**	1/500	1/5000	rb monoclonal	Abcam	EP1536Y, ab51253
**phospho S129 α-Syn**		1/5000	ms monoclonal	Abcam	Clone 81A, ab184674
**PSD95**	1/500		ms monoclonal	Abcam	ab2723
**S100-β**	1/500		ms monoclonal	Abcam	ab 11178
**SQSTM1-p62**	1/400		ms monoclonal	Abcam	ab56416
**Synaptophysin**	1/1000		rb polyclonal	Millipore	AB16659
**TBR1**	1/500		rb polyclonal	abcam	ab31940
**α-Synuclein**	1/500		ms monoclonal	ThermoFisher Scientific	Clone syn211, MS-1572
**α-Synuclein**	1/500	1/5000	ms monoclonal	BD Bioscience	Clone 42, 610787
**Ubiquitin**	1/500		ms monoclonal	Abcam	ab7254
**VGLUT1**	1/1000		rb polyclonal	Synaptic System	135 303

SUPPLEMENTAL REFERENCES

Bousset, L., Pieri, L., Ruiz-Arlandis, G., Gath, J., Jensen, P.H., Habenstein, B., Madiona, K., Olieric, V., Bockmann, A., Meier, B.H., *et al.* (2013). Structural and functional characterization of two alpha-synuclein strains. Nat Commun *4*, 2575.

Deleglise, B., Lassus, B., Soubeyre, V., Alleaume-Butaux, A., Hjorth, J.J., Vignes, M., Schneider, B., Brugg, B., Viovy, J.L., and Peyrin, J.M. (2013). Synapto-protective drugs evaluation in reconstructed neuronal network. PLoS One *8*, e71103.

Macia, E., Ehrlich, M., Massol, R., Boucrot, E., Brunner, C., and Kirchhausen, T. (2006). Dynasore, a cell-permeable inhibitor of dynamin. Dev Cell *10*, 839-850.

Monsellier, E., Redeker, V., Ruiz-Arlandis, G., Bousset, L., and Melki, R. (2015). Molecular interaction between the chaperone Hsc70 and the N-terminal flank of huntingtin exon 1 modulates aggregation. J Biol Chem *290*, 2560-2576.

Nicoleau, C., Varela, C., Bonnefond, C., Maury, Y., Bugi, A., Aubry, L., Viegas, P., Bourgois-Rocha, F., Peschanski, M., and Perrier, A.L. (2013). Embryonic Stem Cells Neural Differentiation Qualifies the Role of Wnt/beta-Catenin Signals in Human Telencephalic Specification and Regionalization. Stem Cells *31*, 1763-1774.

Schlegel, R., Dickson, R.B., Willingham, M.C., and Pastan, I.H. (1982). Amantadine and dansylcadaverine inhibit vesicular stomatitis virus uptake and receptor-mediated endocytosis of alpha 2-macroglobulin. Proc Natl Acad Sci U S A *79*, 2291-2295.
Volpicelli-Daley, L.A., Luk, K.C., and Lee, V.M. (2016). Addition of exogenous alpha-synuclein preformed fibrils to primary neuronal cultures to seed recruitment of endogenous alpha-synuclein to Lewy body and Lewy neurite-like aggregates. Nat Protoc *9*, 2135-2146.
